# Novel 1,2,4‐Triazole–Thiopyrimidine Hybrids as COX‐2 Inhibitors: Synthesis, ADME Profiling, Antioxidant Activity, and Molecular Docking

**DOI:** 10.1155/bmri/4477854

**Published:** 2026-03-31

**Authors:** Yuriy Karpenko, Volodymyr Parchenko, Olexandr Panasenko, Oleksii Bihdan, Iryna Pukhalska, Oleg Nikiforov, Nataliia Nahorna, Volodymyr Nahornyi, Olena Roik

**Affiliations:** ^1^ Department of Toxicological and Inorganic Chemistry, Faculty of Pharmacy, Zaporizhzhia State Medical and Pharmaceutical University, Zaporizhzhia, Ukraine; ^2^ Department of Clinical Pharmacy, Pharmacotherapy, Pharmacognosy and Pharmaceutical Chemistry, Faculty of Pharmacy, Zaporizhzhia State Medical and Pharmaceutical University, Zaporizhzhia, Ukraine; ^3^ Department of Drug Technology, Faculty of Pharmacy, Zaporizhzhia State Medical and Pharmaceutical University, Zaporizhzhia, Ukraine; ^4^ Department of Obstetrics, Gynecology and Reproductive Medicine, Faculty of Medicine, Zaporizhzhia State Medical and Pharmaceutical University, Zaporizhzhia, Ukraine; ^5^ Department of Physicocolloid and Analytical Chemistry, Faculty of Pharmacy, Zaporizhzhia State Medical and Pharmaceutical University, Zaporizhzhia, Ukraine; ^6^ Department of Industrial Pharmacy, Kyiv National University of Technology and Design, Kyiv, Ukraine

**Keywords:** 124-triazole derivatives, antioxidant activity, COX-2 selective inhibitors, density functional theory (DFT), DPPH assay, molecular docking, pyrimidine-24-dione, SPLET mechanism

## Abstract

This work reports the design, synthesis, and multilevel evaluation of 20 new S‐alkyl derivatives of 6‐(5‐mercapto‐4‐ethyl‐4H‐1,2,4‐triazol‐3‐yl)pyrimidine‐2,4(1H,3H)‐dione as potential selective cyclooxygenase‐2 (COX‐2) inhibitors with antioxidant properties. The aim was to establish structure–property relationships between the S‐alkyl fragment, antioxidant mechanisms, and COX‐2 inhibition and to identify lead structures for further optimization. Compounds were obtained by stepwise construction of the 1,2,4‐triazole fragment from orotic acid, followed by selective S‐alkylation of the mercaptotriazole, and their structures were confirmed by ^1^H/^13^C NMR, LC‐MS, and elemental analysis. Antiradical activity in vitro was assessed by the DPPH assay with determination of percent inhibition and IC_50_, whereas COX‐1/COX‐2 inhibition was evaluated using a fluorescent enzyme assay to obtain IC_50_ and selectivity index (SI). Density functional theory (DFT) calculations were employed to compare the thermodynamic feasibility of HAT, SET–PT, and SPLET pathways, while molecular docking and molecular dynamics were used to study the affinity and stability of binding to COX‐1 and COX‐2. A clear increase in antiradical activity was observed in the series **17** < ascorbic acid < **14** < **20** < **13** < **19**, reaching 72.34% DPPH inhibition for Compound **19** with IC_50_(DPPH) = 12.15 ± 3.98 *μ*M, considerably surpassing ascorbic acid. Compound **19** also exhibited the lowest IC_50_(COX − 2) = 24.5 ± 0.9 *μ*M with SI ≈ 8.2; Compounds **13** and **20** showed moderate COX‐2 selectivity (SI ≈ 1.2–1.3), whereas **14** and **17** had a COX‐1‐shifted profile reminiscent of classical NSAIDs. DFT analysis indicated predominance of the SPLET mechanism for most systems, with the lowest *Δ* *G* values for **19** and **20**, consistent with their high antioxidant activity. The combined experimental and in silico data identify Compound **19** as a primary lead candidate that combines potent antioxidant effects with pronounced, selective COX‐2 inhibition and highlight **13** and **20** as promising backup structures for further preclinical studies.

## 1. Introduction

Chronic inflammation and oxidative stress are closely interrelated pathophysiological processes that underpin a wide spectrum of cardiovascular, metabolic, neurodegenerative, and neoplastic diseases [1], as sustained overproduction of reactive oxygen and nitrogen species damages biomacromolecules and activates redox‐sensitive transcription factors (e.g., NF‐*κ*B and AP‐1), thereby upregulating proinflammatory cytokines, adhesion molecules, and enzymes such as cyclooxygenase‐2 (COX‐2) [2, 3]. In this context, small molecules capable of simultaneously dampening inflammatory signaling and attenuating oxidative stress are of particular interest as multitarget chemopreventive and therapeutic agents.

Among them, heterocyclic scaffolds—especially 1,2,4‐triazoles and pyrimidines—have emerged as “privileged structures” in medicinal chemistry [4–6] owing to their ability to engage in diverse noncovalent interactions with biological targets and to fine‐tune key physicochemical parameters such as lipophilicity, hydrogen bonding capacity, and metabolic stability [7, 8]. Numerous 1,2,4‐triazole derivatives [9] have been described as antimicrobial [10, 11], anticancer, anticonvulsant, analgesic, and anti‐inflammatory agents [12, 13], including fused systems such as triazolo–thiadiazoles and triazolo–pyridazinones that exhibit potent COX‐2 inhibition and favorable in vivo anti‐inflammatory profiles [14, 15].

**Figure 1:**
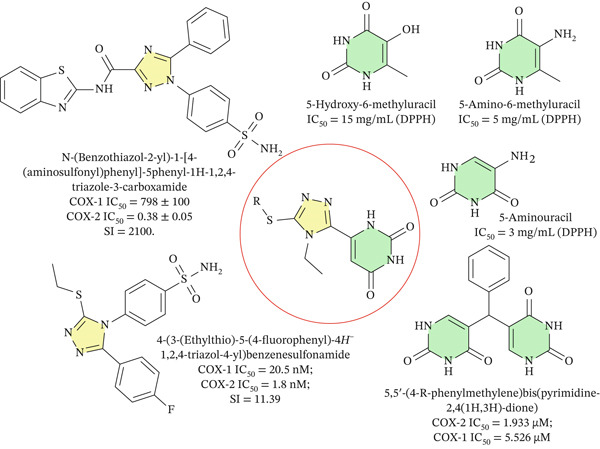
Known 1,2,4‐triazole/pyrimidine‐2,4‐dione hybrids used as reference compounds for scaffold design and S‐functionalization.

Pyrimidine‐containing motifs are equally prominent in bioactive molecules [2]; in particular, the pyrimidine‐2,4‐dione core, structurally related to uracil and orotic acid, can mimic nucleic acid fragments, participate in specific hydrogen bonding, and provide versatile positions for functionalization [16]. Several series of pyrimidine derivatives have been reported as selective COX‐2 inhibitors [17] with concurrent antioxidant properties, often outperforming classical nonsteroidal anti‐inflammatory drugs (NSAIDs) such as piroxicam and approaching or exceeding meloxicam in cellular models, while reducing ROS levels and suppressing proliferation of inflammation‐associated cell lines, which underscores the potential of pyrimidine scaffolds as dual anti‐inflammatory/antioxidant pharmacophores [2, 3]. Hybridization of pharmacophoric heterocycles in a single molecular framework, as exemplified by diaryl 1,2,4‐triazolo [3,4‐a]pyrimidines and other 1,2,4‐triazole‐based hybrids [18] that display selective COX‐2 or dual COX‐2/soluble epoxide hydrolase inhibition together with cardioprotective and anti‐inflammatory effects, further supports the concept that combining 1,2,4‐triazole and pyrimidine‐2,4‐dione motifs in one molecule [19, 20] is a promising strategy for the design of new multifunctional COX‐2‐oriented agents with intrinsic antioxidant activity (Figure 1).

In light of the above considerations, the present study was designed to create and comprehensively characterize a focused library of S‐alkyl derivatives of 6‐(5‐mercapto‐4‐ethyl‐4*H*‐1,2,4‐triazol‐3‐yl)pyrimidine‐2,4(1*H*,3*H*)‐dione as prospective COX‐2 selective anti‐inflammatory agents with intrinsic antioxidant properties. In contrast to earlier triazole–pyrimidine COX inhibitor series that predominantly rely on diaryl/annulated triazolopyrimidines or N‐functionalized motifs, our study introduces an orotic acid–derived pyrimidine‐2,4‐dione scaffold fused to a mercaptotriazole fragment and systematically diversified via selective S‐alkylation, enabling a targeted evaluation of the S‐substituent contribution to COX isoform bias and antiradical behavior.

## 2. Materials and Methods

### 2.1. Chemistry

The structural characterization of the synthesized products was performed by ^1^H and ^13^C NMR spectroscopy on a Bruker spectrometer in DMSO‐d6; tetramethylsilane served as the internal reference. LC‐MS measurements were conducted on an Agilent 1200 LC/MSD SL chromatographic system equipped with DAD (215/241 nm), ELSD, and MSD1‐Pos quadrupole MS detectors. Elemental analysis for C, H, N, and S was carried out with an ELEMENTAR vario EL cube using sulfanilamide as the reference material. The melting points were established by the capillary procedure on a Stanford Research Systems MPA 100 instrument (United States). Commercially available reagents and solvents from Sigma‐Aldrich (Merck) were used in the synthesis. Compounds **a–c** were obtained according to earlier published protocols [9, 10, 12], while their physical constants matched those previously described in the literature [19, 21]. The complete ^1^H and ^13^C NMR spectra, LC‐MS data, and elemental analysis data for all synthesized compounds are provided in the Supporting Information (available here) section.

#### 2.1.1. Synthesis of 6‐(5‐Mercapto‐4‐Ethyl‐4H‐1,2,4‐Triazol‐3‐yl)Pyrimidine‐2,4(1H,3H)‐Dione **1–2** (General Methods)

To a suspension of orotic acid hydrazide c (10 mmol) in propan‐2‐ol (20 mL), a solution of ethyl isothiocyanate (10 mmol) in propan‐2‐ol (5 mL) was added dropwise with heating. The reaction mixture was maintained under heating for 2 h. After completion of the reaction, the formed solid was isolated by filtration and dried to afford the intermediate carbothioamide 1, which was used in the following step without additional purification.

The obtained 2‐(2,6‐dioxo‐1,2,3,6‐tetrahydropyrimidine‐4‐carbonyl)‐*N*‐ethylhydrazine‐1‐carbothioamide (10 mmol) was then treated with sodium hydroxide (10 mmol) in purified water (20 mL), and the reaction mixture was heated under reflux for 2 h. Upon completion, the mixture was allowed to cool completely, after which concentrated acetic acid (2 mL) was added to the filtrate. The precipitated product was collected by filtration, washed thoroughly with purified water, and recrystallized from DMF to obtain analytically pure material. The final product was isolated as a light yellow powder; it was found to be soluble in aqueous alkaline media, DMF, and 1,4‐dioxane.

2‐(2,6‐Dioxo‐1,2,3,6‐tetrahydropyrimidine‐4‐carbonyl)‐N‐ethylhydrazine‐1‐carbothioamide (**1**). Yield 1.96 g (82%), light yellow powder, mp 278°С (DMF). ^1^H NMR(DMSO‐d_6_, 500 MHz), *δ*, ppm (J, Hz): 1.05 (t, 3H, J = 7.0, –CH_3_), 3.39–3.48 (m, 2H, –CH_2_–CH_3_), 5.91 (s, 2H, H‐3 pyrimidine, –NH–CH_2_–CH_3_), 7.74 (s, 2H, H‐1,5 pyrimidine), 8.37 (s, 1H, –NH–NH–), 8.84 (s, 1H, –NH–NH–). ^13^C NMR, *δ*, ppm: 14.85, 97.62, 99.46, 146.11, 152.12, 160.06, 164.82, 180.99. LC‐MS (ESI+, positive mode): *t*_R_ = 0.550 min; *m*/*z* 211.0/212.0 (major ion). Anal. calcd. for C_8_H_11_N_5_O_3_S: C: 37.35%; H: 4.31%; N: 27.22%; S: 12.46%. Found: C: 37.47%; H: 4.11%; N: 27.74%; S: 12.53%.

6‐(5‐Mercapto‐4‐ethyl‐4H‐1,2,4‐triazol‐3‐yl)pyrimidine‐2,4(1H,3H)‐dione (**2**). Yield 1.72 g (72%), white powder, mp 266°С (DMF). ^1^H NMR(DMSO‐d_6_, 500 MHz), *δ*, ppm (J, Hz): 1.20 (t, 3H, J = 7.4, CH_3_), 4.04 (q, 2H, J = 7.3, –N–CH_2_–CH_3_), 5.94 (d, 1H, J = 5.8, H‐3 pyrimidine), 11.36 (s, 2H, H‐1,5 pyrimidine), 14.12 (s, 1H, SH). LC‐MS (ESI+, positive mode): *t*_R_ = 0.731 min; *m*/*z* 207.0/208.0 (major ions). Anal. calcd. for C_8_H_9_N_5_O_2_S: C: 40.16%; H: 3.79%; N: 29.27%; S: 13.40%. Found: C: 40.22%; H: 3.64%; N: 29.21%; S: 13.44%.

#### 2.1.2. Preparation of S‐Alkyl Derivatives of 6‐(5‐Mercapto‐4‐Ethyl‐4H‐1,2,4‐Triazol‐3‐yl)Pyrimidine‐2,4(1H,3H)‐Dione **3–20** (General Methods)

6‐(5‐Mercapto‐4‐ethyl‐4H‐1,2,4‐triazol‐3‐yl)pyrimidine‐2,4(1*H*,3*H*)‐dione (5 mmol) was treated with NaOH (5 mmol) in propan‐2‐ol (10 mL), followed by the addition of the appropriate halogen derivative (5 mmol). The reaction mixture was heated for 2 h, then allowed to cool. The precipitated product was filtered off, washed with purified water, and recrystallized from methanol. In this way, Compounds **3–20** were obtained as yellow or brown solids, or in some cases as oily products, showing poor solubility in water and good solubility in organic media.

6‐(4‐Ethyl‐5‐(methylthio)‐4*H*‐1,2,4‐triazol‐3‐yl)pyrimidine‐2,4(1*H*,3*H*)‐dione (**3**). Yield 0.96 g (76%), brown powder, mp 135°С (MeOH). ^1^H NMR(DMSO‐d_6_, 500 MHz), *δ*, ppm (J, Hz): 1.39–1.46 (m, 3H, –N–CH_2_–CH_3_), 2.69 (s, 2H, –S–CH_2_–), 4.29 (q, 2H, J = 6.1, –N–CH_2_–CH_3_), 6.14 (s, 1H, H‐3 pyrimidine), 11.16 (s, 1H, H‐1 pyrimidine), 11.62 (s, 1H, H‐5 pyrimidine). LC‐MS (ESI+, positive mode): *t*_R_ = 0.93 min; *m*/*z* 254.2/256.2 (major ions). Mass spectrum, *m*/*z* (Irel, %) 254 [M + Н]^+^ (100). Anal. calcd. for C_9_H_11_N_5_O_2_S: C: 42.68%; H: 4.38%; N: 27.65%; S: 12.66%. Found: C: 42.38%; H: 4.14%; N: 27.73%; S: 12.58%.

6‐(4‐Ethyl‐5‐(ethylthio)‐4*H*‐1,2,4‐triazol‐3‐yl)pyrimidine‐2,4(1*H*,3*H*)‐dione (**4**). Yield 0.97 g (73%), brown powder, mp 145°С (MeOH). ^1^H NMR(DMSO‐d_6_, 500 MHz), *δ*, ppm (J, Hz): 1.36 (t, 3H, J = 6.2, –S–CH_2_–CH_3_), 1.39–1.46 (m, 3H, –N–CH_2_–CH_3_), 3.07 (q, 2H, J = 6.2, –S–CH_2_–CH_3_), 4.30 (q, 2H, J = 6.2, –N–CH_2_–CH_3_), 6.14 (s, 1H, H‐3 pyrimidine), 11.16 (s, 1H, H‐1 pyrimidine), 11.62 (s, 1H, H‐5 pyrimidine). ^13^C NMR(DMSO‐d_6_, 125 MHz), *δ*, ppm: 14.45, 14.91, 15.36, 24.15, 26.98, 38.87, 94.75, 159.45, 166.60, 174.47. LC‐MS (ESI+, positive mode): *t*_R_ = 1.706 min; *m*/*z* 313.2/315.2 (major ions). Anal. calcd. for C_10_H_13_N_5_O_2_S: C: 44.93%; H: 4.90%; N: 26.20%; S: 11.99%. Found: C: 44.85%; H: 4.78%; N: 26.35%; S: 11.79%.

6‐(4‐Ethyl‐5‐(propylthio)‐4*H*‐1,2,4‐triazol‐3‐yl)pyrimidine‐2,4(1*H*,3*H*)‐dione (**5**). Yield 1.05 g (75%), brown powder, mp 148°С (MeOH). ^1^H NMR(DMSO‐d_6_, 400 MHz), *δ*, ppm (J, Hz): 1.05 (t, 3H, J = 7.1, –S–CH_2_–CH_2_–CH_3_), 1.39–1.46 (m, 3H, –N–CH_2_–CH_3_), 1.76 (qt, 2H, J = 7.0, 5.3, –S–CH_2_–CH_2_–CH_3_), 3.10 (t, 2H, J = 5.3, –S–CH_2_–CH_2_–CH_3_), 4.30 (q, 2H, J = 6.2, –N–CH_2_–CH_3_), 6.14 (s, 1H, H‐3 pyrimidine), 11.16 (s, 1H, H‐1 pyrimidine), 11.62 (s, 1H, H‐5 pyrimidine). ^13^C NMR(DMSO‐d_6_, 125 MHz), *δ*, ppm: 12.95, 14.13, 22.64, 34.04 (2C), 98.23, 137.74, 150.51, 150.60, 156.15, 164.98 ppm. LC‐MS (ESI+, positive mode): *t*_R_ = 0.80 min; *m*/*z* 310.2, 332.2 (major ions). Anal. calcd. for C_11_H_15_N_5_O_2_S: C: 46.96%; H: 5.37%; N: 24.89%; S: 11.40%. Found: C: 46.91%; H: 5.45%; N: 24.81%; S: 11.49%.

6‐(5‐(Butylthio)‐4‐ethyl‐4*H*‐1,2,4‐triazol‐3‐yl)pyrimidine‐2,4(1*H*,3*H*)‐dione (**6**). Yield 1.00 g (68%), brown powder, mp 151°С (MeOH). ^1^H NMR(DMSO‐d_6_, 500 MHz), *δ*, ppm (J, Hz): 0.92 (t, 3H, J = 7.1, –CH_3_), 1.34–1.46 (m, 3H, –N–CH_2_–CH_3_), 1.67 (ddd, 4H, J = 12.9, 6.8, 6.1, –CH_2_–), 3.15 (t, 2H, J = 6.7, S–CH_2_–), 4.30 (q, 2H, J = 6.2, –N–CH_2_–CH_3_), 6.14 (s, 1H, H‐3 pyrimidine), 11.16 (s, 1H, H‐1 pyrimidine), 11.62 (s, 1H, H‐5 pyrimidine). LC‐MS (ESI+, positive mode): *t*_R_ = 0.79 min; *m*/*z* 229.2/231.2, 290.2 (major ions). Anal. calcd. for C_12_H_17_N_5_O_2_S: C: 48.80%; H: 5.80%; N: 23.71%; S: 10.85%. Found: C: 48.82%; H: 5.91%; N: 23.63%; S: 10.92%.

6‐(4‐Ethyl‐5‐(pentylthio)‐4*H*‐1,2,4‐triazol‐3‐yl)pyrimidine‐2,4(1*H*,3*H*)‐dione (**7**). Yield 1.05 g (68%), brown powder, mp 156°С (MeOH). ^1^H NMR(DMSO‐d_6_, 400 MHz), *δ*, ppm (J, Hz): 0.84–0.93 (m, 3H, –CH_3_), 1.33–1.46 (m, 3H, –N–CH_2_–CH_3_), 1.68–1.77 (m, 6H, –CH_2_–), 3.10 (t, 2H, J = 6.0, S–CH_2_–), 4.30 (q, 2H, J = 6.2, –N–CH_2_–CH_3_), 6.14 (s, 1H, H‐3 pyrimidine), 11.16 (s, 1H, H‐1 pyrimidine), 11.62 (s, 1H, H‐5 pyrimidine). LC‐MS (ESI+, positive mode): *t*_R_ = 1.61 min; *m*/*z* 313.4/315.4 (major ions). Anal. calcd. for C_13_H_19_N_5_O_2_S: C: 50.47%; H: 6.19%; N: 22.64%; S: 10.36%. Found: C: 50.52%; H: 6.11%; N: 22.68%; S: 10.25%.

6‐(4‐Ethyl‐5‐(hexylthio)‐4*H*‐1,2,4‐triazol‐3‐yl)pyrimidine‐2,4(1*H*,3*H*)‐dione (**8**). Yield 1.16 g (72%), brown powder, mp 158°С (MeOH). ^1^H NMR(DMSO‐d_6_, 500 MHz), *δ*, ppm (J, Hz): 0.84–0.93 (m, 3H, –CH_3_), 1.25–1.46 (m, 3H, –N–CH_2_–CH_3_), 1.68 (p, 8H, J = 6.2, –CH_2_–), 3.12 (t, 2H, J = 6.4, S–CH_2_–), 4.30 (q, 2H, J = 6.2, –N–CH_2_–CH_3_), 6.14 (s, 1H, H‐3 pyrimidine), 11.16 (s, 1H, H‐1 pyrimidine), 11.62 (s, 1H, H‐5 pyrimidine). LC‐MS (ESI+, positive mode): *t*_R_ = 0.91 min; *m*/*z* 338.2/339.2 (major ions). Anal. calcd. for C_14_H_21_N_5_O_2_S: C: 51.99%; H: 6.55%; N: 21.65%; S: 9.91%. Found: C: 51.86%; H: 6.34%; N: 21.88%; S: 9.98%.

6‐(4‐Ethyl‐5‐(heptylthio)‐4*H*‐1,2,4‐triazol‐3‐yl)pyrimidine‐2,4(1*H*,3*H*)‐dione (**9**). Yield 0.88 g (52%), brown powder, mp 163°С–165°С (MeOH). ^1^H NMR(DMSO‐d_6_, 400 MHz), *δ*, ppm (J, Hz): 0.40–0.50 (m, 3H, –CH_3_), 0.78–0.91 (m, 6H, –CH_2_–), 0.91–0.99 (m, 2H, –CH_2_–), 1.00 (t, 3H, J = 6.1 Hz, –N–CH_2_–CH_3_), 1.25 (p, 2H, J = 6.5, –CH_2_–), 2.69 (t, 2H, J = 6.4, S–CH_2_–), 3.87 (q, 2H, J = 6.2, –N–CH_2_–CH_3_), 5.71 (s, 1H, H‐3 pyrimidine), 10.73 (s, 1H, H‐1 pyrimidine), 11.19 (s, 1H, H‐5 pyrimidine). LC‐MS (ESI+, positive mode): *t*_R_ = 0.94 min; *m*/*z* 338.2 (major ions). Mass spectrum, *m*/*z* (Irel, %) 338 [M + Н]^+^ (100). Anal. calcd. for C_15_H_23_N_5_O_2_S: C: 53.39%; H: 6.87%; N: 20.75%; S: 9.50%. Found: C: 53.45%; H: 6.54%; N: 20.87%; S: 9.42%.

6‐(4‐Ethyl‐5‐(octylthio)‐4*H*‐1,2,4‐triazol‐3‐yl)pyrimidine‐2,4(1*H*,3*H*)‐dione (**10**). Yield 1.46 g (83%), brown powder, mp 172°С (MeOH). ^1^H NMR(DMSO‐d_6_, 500 MHz), *δ*, ppm (J, Hz): 0.83–0.93 (m, 3H, –CH_3_), 1.25 (s, 2H, –CH_2_–), 1.21–1.40 (m, 8H, –CH_2_–), 1.40–1.46 (m, 3H, –N–CH_2_–CH_3_), 1.68 (p, 2H, J = 6.5, –CH_2_–), 3.12 (t, 2H, J = 6.4, S–CH_2_–), 4.30 (q, 2H, J = 6.2, –N–CH_2_–CH_3_), 6.14 (s, 1H, H‐3 pyrimidine), 11.16 (s, 1H, H‐1 pyrimidine), 11.62 (s, 1H, H‐5 pyrimidine). LC‐MS (ESI+, positive mode): *t*_R_ = 0.96 min; *m*/*z* 352.2, 374.2 (major ions, peak). Anal. calcd. for C_16_H_25_N_5_O_2_S: C: 54.68%; H: 7.17%; N: 19.93%; S: 9.12%. Found: C: 54.77%; H: 7.11%; N: 19.88%; S: 9.54%.

6‐(4‐Ethyl‐5‐(nonylthio)‐4*H*‐1,2,4‐triazol‐3‐yl)pyrimidine‐2,4(1*H*,3*H*)‐dione (**11**). Yield 1.35 g (74%), brown powder, mp 184°С (MeOH). ^1^H NMR(DMSO‐d_6_, 400 MHz), *δ*, ppm (J, Hz): 0.84–0.93 (m, 3H, –CH_3_), 1.21–1.32 (m, 12H, –CH_2_–), 1.36 (ttd, 2H, J = 7.1, 6.3, 0.8, –CH_2_–), 1.42 (t, 3H, J = 6.1, –N–CH_2_–CH_3_), 1.68 (p, 2H, J = 6.5, –CH_2_–), 3.12 (t, 2H, J = 6.4, S–CH_2_–), 4.30 (q, 2H, J = 6.2, –N–CH_2_–CH_3_), 6.14 (s, 1H, H‐3 pyrimidine), 11.16 (s, 1H, H‐1 pyrimidine), 11.62 (s, 1H, H‐5 pyrimidine). LC‐MS (ESI−, negative mode): *t*_R_ = 1.04 min; *m*/*z* 364.2/366.2 (major ions). Anal. calcd. for C_17_H_27_N_5_O_2_S: C: 55.87%; H: 7.45%; N: 19.16%; S: 8.77%. Found: C: 55.92%; H: 7.31%; N: 19.11%; S: 8.92%.

6‐(5‐(Decylthio)‐4‐ethyl‐4*H*‐1,2,4‐triazol‐3‐yl)pyrimidine‐2,4(1*H*,3*H*)‐dione (**12**). Yield 1.57 g (83%), yellow powder, mp 198°С (MeOH). ^1^H NMR(DMSO‐d_6_, 500 MHz), *δ*, ppm (J, Hz): 0.84–0.93 (m, 3H, –CH_3_), 1.20–1.31 (m, 12H, –CH_2_–), 1.36 (ttd, 2H, J = 7.1, 6.2, 0.8, –CH_2_–), 1.42 (t, 3H, J = 6.1, –N–CH_2_–CH_3_), 1.68 (p, 2H, J = 6.5, –CH_2_–), 3.12 (t, 2H, J = 6.4, –S–CH_2_–), 4.30 (q, 2H, J = 6.2, –N–CH_2_–CH_3_), 6.14 (s, 1H, H‐3 pyrimidine), 11.16 (s, 1H, H‐1 pyrimidine), 11.62 (s, 1H, H‐5 pyrimidine). LC‐MS (ESI+, positive mode): *t*_R_ = 1.69 min; *m*/*z* 313.2/315.2 (major ions). Anal. calcd. for C_18_H_29_N_5_O_2_S: C: 56.97%; H: 7.70%; N: 18.45%; S: 8.45%. Found: C: 56.91%; H: 7.78%; N: 18.52%; S: 8.41%.

2‐((5‐(2,6‐Dioxo‐1,2,3,6‐tetrahydropyrimidin‐4‐yl)‐4‐ethyl‐4H‐1,2,4‐triazol‐3‐yl)thio) acetic acid (**13**). Yield 1.20 g (81%), white powder, mp 214°С (MeOH). ^1^H NMR(DMSO‐d_6_, 500 MHz), *δ*, ppm (J, Hz): 1.39–1.46 (m, 3H, –N–CH_2_–CH_3_), 4.07 (s, 2H, –S–CH_2_–), 4.31 (q, 2H, J = 6.2, –N–CH_2_–CH_3_), 6.14 (s, 1H, H‐3 pyrimidine), 11.16 (s, 1H, H‐1 pyrimidine), 11.36 (s, 1H, –COOH), 11.62 (s, 1H, H‐5 pyrimidine). LC‐MS (ESI+, positive mode): *t*_R_ = 1.15 min; *m*/*z* 298.2 (major ions). Anal. calcd. for C_10_H_11_N_5_O_4_S: C: 40.40%; H: 3.73%; N: 23.56%; S: 10.78%. Found: C: 40.45%; H: 3.62%; N: 23.82%; S: 10.54%.

Morpholin‐4‐ium 2‐((5‐(2,6‐dioxo‐1,2,3,6‐tetrahydropyrimidin‐4‐yl)‐4‐ethyl‐4H‐1,2,4‐triazol‐3‐yl)thio) acetate (**14**). Yield 1.32 g (69%), brown powder, mp 195°С (MeOH). LC‐MS (ESI+, positive mode): *t*_R_ = 1.08 min; *m*/*z* 385.2 (major ions). Anal. calcd. for C_14_H_20_N_6_O_5_S: C: 43.74%; H: 5.24%; N: 21.86%; S: 8.34%. Found: C: 43.61%; H: 5.43%; N: 21.92%; S: 8.27%.

Methyl 2‐((5‐(2,6‐dioxo‐1,2,3,6‐tetrahydropyrimidin‐4‐yl)‐4‐ethyl‐4H‐1,2,4‐triazol‐3‐yl)thio) acetate (**15**). Yield 1.36 g (88%), light yellow powder, mp 218°С–220°С (MeOH). ^1^H NMR(DMSO‐d_6_, 400 MHz), *δ*, ppm (J, Hz): 1.39–1.46 (m, 3H, –N–CH_2_–CH_3_), 3.72 (s, 3H, –CH_3_), 4.07 (s, 2H, –S–CH_2_–), 4.31 (q, 2H, J = 6.2 Hz, –N–CH_2_–CH_3_), 6.14 (s, 1H, H‐3 pyrimidine), 11.16 (s, 1H, H‐1 pyrimidine), 11.62 (s, 1H, H‐5 pyrimidine). LC‐MS (ESI+, positive mode): *t*_R_ = 0.61 min; *m*/*z* 302.2, 312.2, 364.2 (major ions). Anal. calcd. for C_11_H_13_N_5_O_4_S: C: 42.44%; H: 4.21%; N: 22.50%; S: 10.30%. Found: C: 42.64%; H: 4.15%; N: 22.58%; S: 10.23%.

Ethyl 2‐((5‐(2,6‐dioxo‐1,2,3,6‐tetrahydropyrimidin‐4‐yl)‐4‐ethyl‐4*H*‐1,2,4‐triazol‐3‐yl)thio) acetate (**16**). Yield 1.20 g (72%), light yellow powder, mp 224°С (MeOH). ^1^H NMR(DMSO‐d_6_, 500 MHz), *δ*, ppm (J, Hz): 1.20–1.27 (m, 3H, –CH_2_–CH_3_), 1.39–1.46 (m, 3H, –N–CH_2_–CH_3_), 4.07 (s, 2H, –S–CH_2_–), 4.16 (q, 2H, J = 6.6, –CH_2_–CH_3_), 4.31 (q, 2H, J = 6.2, –N–CH_2_–CH_3_), 6.14 (s, 1H, H‐3 pyrimidine), 11.16 (s, 1H, H‐1 pyrimidine), 11.62 (s, 1H, H‐5 pyrimidine). LC‐MS (ESI+, positive mode): *t*_R_ = 0.63 min; *m*/*z* 326.4 (major ion). Anal. calcd. for C_12_H_15_N_5_O_4_S: C: 44.30%; H: 4.65%; N: 21.53%; S: 9.85%. Found: C: 44.35%; H: 4.59%; N: 21.68%; S: 9.72%.

Isopropyl 2‐((5‐(2,6‐dioxo‐1,2,3,6‐tetrahydropyrimidin‐4‐yl)‐4‐ethyl‐4*H*‐1,2,4‐triazol‐3‐yl)thio) acetate (**17**). Yield 1.49 g (88%), yellow powder, mp 212°С (MeOH). ^1^H NMR(DMSO‐d_6_, 500 MHz), *δ*, ppm (J, Hz): 1.21 (d, 6H, J = 6.0, –CH_3_), 1.39–1.46 (m, 3H, –N–CH_2_–CH_3_), 4.09 (s, 2H, –S–CH_2_–), 4.31 (q, 2H, J = 6.2, –N–CH_2_–CH_3_), 4.95 (p, 1H, J = 5.9, –CH–), 6.14 (s, 1H, H‐3 pyrimidine), 11.16 (s, 1H, H‐1 pyrimidine), 11.62 (s, 1H, H‐5 pyrimidine). LC‐MS (ESI+, positive mode): *t*_R_ = 0.69 min; *m*/*z* 340.2, 362.0/363.2 (major ions). Anal. calcd. for C_13_H_17_N_5_O_4_S: C: 46.01%; H: 5.05%; N: 20.64%; S: 9.45%. Found: C: 46.11%; H: 5.09%; N: 20.56%; S: 9.33%.

Butyl 2‐((5‐(2,6‐dioxo‐1,2,3,6‐tetrahydropyrimidin‐4‐yl)‐4‐ethyl‐4*H*‐1,2,4‐triazol‐3‐yl)thio) acetate (**18**). Yield 1.52 g (86%), yellow powder, mp 244°С (MeOH). ^1^H NMR(DMSO‐d_6_, 500 MHz), *δ*, ppm (J, Hz): 0.92 (t, 3H, J = 7.0, –CH_3_), 1.30–1.39 (m, 2H, –CH_2_–), 1.37–1.46 (m, 3H, –N–CH_2_–CH_3_), 1.56–1.65 (m, 2H, –CH_2_–), 4.05–4.11 (m, 4H, CH_2_–), 4.31 (q, 2H, J = 6.2, –N–CH_2_–CH_3_), 6.14 (s, 1H, H‐3 pyrimidine), 11.16 (s, 1H, H‐1 pyrimidine), 11.62 (s, 1H, H‐5 pyrimidine). LC‐MS (ESI+, positive mode): *t*_R_ = 0.82 min; *m*/*z* 354.2 (major ion). Anal. calcd. for C_14_H_19_N_5_O_4_S: C: 47.58%; H: 5.42%; N: 19.82%; S: 9.07%. Found: C: 47.82%; H: 5.31%; N: 19.74%; S: 9.12%.

2‐((5‐(2,6‐Dioxo‐1,2,3,6‐tetrahydropyrimidin‐4‐yl)‐4‐ethyl‐4*H*‐1,2,4‐triazol‐3‐yl)thio) acetamide (**19**). Yield 1.36 g (92%), white powder, mp 194°С (MeOH). ^1^H NMR(DMSO‐d_6_, 500 MHz), *δ*, ppm (J, Hz): 1.39–1.46 (m, 3H, –N–CH_2_–CH_3_), 4.01 (s, 2H, –S–CH_2_–), 4.30 (q, 2H, J = 6.1 Hz, –N–CH_2_–CH_3_), 6.14 (s, 1H, H‐3 pyrimidine), 7.05 (s, 2H, –NH_2_), 11.16 (s, 1H, H‐1 pyrimidine), 11.62 (s, 1H, H‐5 pyrimidine). ^13^C NMR(DMSO‐d_6_, 151 MHz), *δ*, ppm: 14.69, 20.57, 24.34, 36.83, 61.97, 150.06, 168.68, 169.93, 174.63, 174.70. LC‐MS (ESI+, positive mode): *t*_R_ = 0.80 min; *m*/*z* 338.2 (major ion). Anal. calcd. for C_10_H_12_N_6_O_3_S: C: 40.54%; H: 4.08%; N: 28.36%; S: 10.82%. Found: C: 40.43%; H: 4.14%; N: 28.32%; S: 10.87%.

2‐((5‐(2,6‐Dioxo‐1,2,3,6‐tetrahydropyrimidin‐4‐yl)‐4‐ethyl‐4*H*‐1,2,4‐triazol‐3‐yl)thio) acetohydrazide (**20**). Yield 1.11 g (71%), white powder, mp 252°С (MeOH). ^1^H NMR(DMSO‐d_6_, 400 MHz), *δ*, ppm (J, Hz): 1.39–1.46 (m, 3H, –N–CH_2_–CH_3_), 3.88 (s, 2H, –S–CH_2_–), 4.16 (d, 2H, J = 3.8 Hz, –NH–NH_2_), 4.30 (q, 2H, J = 6.1 Hz, –N–CH_2_–CH_3_), 6.14 (s, 1H, H‐3 pyrimidine), 9.16 (t, 1H, J = 3.8 Hz, –NH–NH_2_), 11.16 (s, 1H, H‐1 pyrimidine), 11.62 (s, 1H, H‐5 pyrimidine). LC‐MS (ESI+, positive mode): *t*_R_ = 0.596 min; *m*/*z* 231.0, 245.0, 256.8, 303.0/305.8, 361.8 (major ions). Anal. calcd. for C_10_H_13_N_7_O_3_S: C: 38.58%; H: 4.21%; N: 31.49%; S: 10.30%. Found: C: 38.61%; H: 4.83%; N: 31.52%; S: 10.68%.

### 2.2. Biological Activity

#### 2.2.1. Antiradical Activity

The free radical scavenging properties of the synthesized compounds were assessed using the 1,1‐diphenyl‐2‐picrylhydrazyl (DPPH) assay [22]. A 0.001 M solution of each test substance was first prepared in DMSO using a 25.00‐mL volumetric flask. This stock solution was then diluted by transferring 1.00 mL into a 10.00‐mL volumetric flask and making up to volume with DMSO, yielding a 0.0001 M solution [23].

An aliquot (2.00 mL) of the diluted sample solution was mixed with 2.00 mL of 0.1 mM DPPH in methanol. The reaction mixtures were shaken thoroughly, protected from light, and maintained at room temperature for 30 min. The absorbance of each solution was then measured at 516 nm. A mixture of 2.00 mL of DPPH solution and 2.00 mL of methanol was used as the control, whereas ascorbic acid served as the positive reference. The antioxidant effect was expressed as percent inhibition of the DPPH radical and determined according to the following equation:

%antiradical activity=A0−A1A0×100,

where *A*_0_ is the absorbance of the control sample and *A*_1_ is the absorbance of the test sample. The absorbance of the studied solutions was measured in aqueous–organic solutions, with the absorption maximum at 516 nm recorded using a SPECORD 250 spectrophotometer. IC_50_ values were derived from concentration–response curves by nonlinear regression (four‐parameter logistic [4PL]). Each concentration was measured in at least triplicate (independent preparations), and results are reported as mean ± standard deviation (SD); goodness of fit was inspected visually and by residual distribution.

#### 2.2.2. COX Inhibitor Screening

The inhibitory activity of the compounds against COX‐1 and COX‐2 isoforms was evaluated using an enzymatic microplate‐based assay and a commercial COX Fluorescent Inhibitor Screening Assay Kit (Cayman Chemical, Ann Arbor, Michigan, United States; Cat. No. 700100) [24], which relies on fluorometric detection of the product formed during the conversion of arachidonic acid to PGG_2_/PGH_2_ [25].

Tested compounds were incubated with recombinant COX‐1 and COX‐2 in 96‐well microplates in the presence of the assay buffer supplied by the manufacturer. The reaction was initiated by the addition of arachidonic acid. The rate of product formation was monitored by measuring fluorescence intensity at the appropriate excitation/emission wavelengths, according to the manufacturer’s instructions. For each compound, several concentrations were tested over a range spanning below and above the expected IC_50_ value; each concentration was assayed in at least duplicate or triplicate [26, 27].

Wells without an inhibitor (100% enzyme activity) served as negative controls, whereas ibuprofen at a known concentration was used as a positive control. In parallel, control measurements with DMSO at a concentration matching its content in the test wells were performed to exclude any solvent effects.

Fluorescence signals were corrected for blank values, and the percentage of enzyme inhibition at each compound concentration was calculated relative to the inhibitor‐free control using the following equation:

%inhibition=100×Acontrol−AsampleAcontrol,

where *A*_control_ is the signal in the absence of an inhibitor and *A*_sample_ is the signal in the presence of the tested compound.

Concentration–response curves (% inhibition vs. log *C*, where *C* is the compound concentration) were fitted to a 4PL model using GraphPad Prism software to determine IC_50_ values, defined as the compound concentration producing 50% inhibition of enzyme activity. The selectivity index (SI) was calculated as the ratio IC_50_(COX‐1)/IC_50_(COX‐2). All results are expressed as mean ± SD of at least three independent experiments. Statistical significance of differences between groups was assessed using appropriate tests (e.g., one‐way ANOVA followed by post hoc multiple‐comparison analysis), with *p* < 0.05 considered statistically significant.

### 2.3. Quantum Chemical Calculations [28, 29]

Thermodynamic parameters of the potential antioxidant mechanisms hydrogen atom transfer (HAT), single‐electron transfer–proton transfer (SET–PT), and sequential proton loss–electron transfer (SPLET) for the series of hybrid Derivatives **1**–**20** were calculated using density functional theory (DFT) [30–33] as implemented in the ORCA 6.1.0 program package [34, 35], employing the standard “fast” level of theory. Full geometry optimizations followed by harmonic vibrational frequency calculations were performed for the neutral species, radicals, radical cations, and anions in order to confirm the nature of the stationary points (no imaginary frequencies) and to obtain thermochemical corrections to the Gibbs free energy at 298 K.

Solvent effects (water and methanol) were taken into account using the conductor‐like polarizable continuum model (CPCM) [36]. Based on the resulting *G*_298_ values, standard Gibbs free energy changes *Δ* *G*_298_ (kJ/mol) were computed for the first step of the HAT_NH_ mechanism, the initial single‐electron transfer step of the SET–PT pathway, and the SPLET_NH_ mechanism (sequential proton dissociation followed by electron transfer) [37]. For methanol, additional alternative solvated configurations (MeOH(A)/MeOH(B), corresponding to different orientations of solvent molecules near the reactive site) were considered, and the lowest *Δ* *G* value for each compound and mechanism was used in the subsequent analysis. N–H bond dissociation energies (BDE_NH_) for the radical forms R_rad_NH were calculated from the total energies of the AH, A•, and H• species [38].

### 2.4. Molecular Docking

Molecular docking of the **1**–**20** derivatives was performed using smina (a fork of AutoDock Vina) [39], which enables prediction of the optimal binding pose and binding energy of ligands within the active site of target proteins. Protein structures (PDB IDs: 3N8Z, 3LN1, 5KIR, 5F19, and 1EQG) were retrieved from the Protein Data Bank [40] and preprocessed by removing crystallographic water molecules and nonprotein heteroatoms. Missing hydrogen atoms were then added, and protonation states were assigned according to physiological pH 7.4. The prepared receptors were converted to PDBQT format for subsequent docking. Protocol validation was performed by redocking the cocrystallized ligand; pose reproduction was assessed by RMSD relative to the crystal pose.

Ligand structures were generated from their SMILES representations. For each compound, a three‐dimensional (3D) conformation was built, subjected to geometry optimization, and protonated at pH 7.4. The ligands were then converted to PDBQT format required by the docking engine.

The docking procedure [41] involved automatic definition of the grid box center based on the position of the cocrystallized ligand (when available); otherwise, the center was placed at the geometric center of the active site. Grid box dimensions were chosen to fully encompass the COX catalytic pocket and to allow accommodation of the flexible S‐alkyl chain (typically 22–26 Å per side, depending on the receptor). Docking was performed using the standard smina/Vina scoring function with default search settings (exhaustiveness = 8, num_modes = 9, energy_range = 3 kcal/mol) to ensure comparable sampling across all receptor–ligand pairs. For each receptor–ligand pair, multiple binding poses were calculated, and the conformation with the lowest (most favorable) binding energy was selected for further analysis. The resulting protein–ligand complexes were exported as merged receptor–ligand PDB structures (best‐ranked pose) and used for interaction annotation (PLIP) and visual inspection.

The interaction patterns in the selected top‐scoring complexes were examined using PLIP [42], which automatically identifies hydrogen bonds, hydrophobic contacts, *π*–*π* and *π*–cation interactions, salt bridges, and other types of noncovalent contacts. Final visualization of the 3D structures, inspection of ligand orientation within the active site, and preparation of graphical materials for the manuscript were performed in Maestro 14.0 [43].

This integrated workflow provided a reproducible, standardized, and largely automated docking protocol [44], enabling a meaningful comparison of binding affinities and binding modes of all **1–20** derivatives across the different biological targets.

### 2.5. In Silico Absorption, Distribution, Metabolism, and Excretion (ADME) Profiling

Predicted ADME [45] properties of the synthesized S‐alkyl 6‐(5‐mercapto‐4‐ethyl‐4*H*‐1,2,4‐triazol‐3‐yl)pyrimidine‐2,4(1*H*,3*H*)‐dione derivatives were evaluated using the SwissADME web tool (http://www.swissadme.ch) [46]. Canonical SMILES of each compound were submitted to SwissADME, and the following descriptors were analyzed: basic physicochemical parameters (molecular weight, lipophilicity, topological polar surface area, number of H‐bond donors/acceptors, and rotatable bonds), water solubility (ESOL and related models), pharmacokinetic predictors (gastrointestinal [GI] absorption, blood–brain barrier penetration, P‐glycoprotein substrate status, and CYP450 inhibition), drug‐likeness filters (Lipinski, Veber, Egan, Ghose, and Muegge), and medicinal chemistry alerts (PAINS and Brenk) [47–49]. In addition, the “bioavailability radar” provided a graphical overview of oral drug‐likeness and passive absorption/brain penetration for each compound.

## 3. Results and Discussion

### 3.1. Synthesis

**Figure 2:**
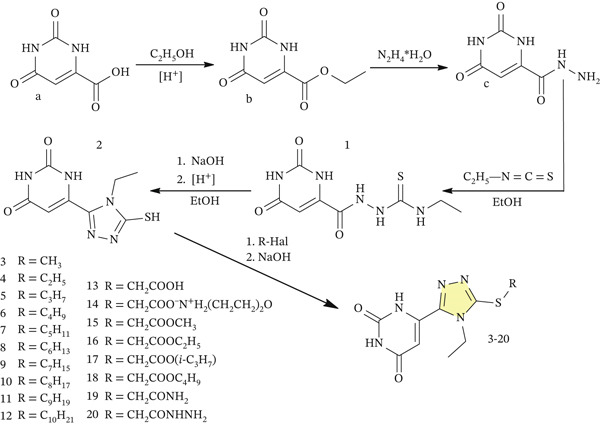
Synthesis of hybrid derivatives combining an orotic acid (pyrimidine‐2,4‐dione) core and a 1,2,4‐triazole‐3(2*H*)‐thione fragment.

The synthesis of 6‐(5‐mercapto‐4‐ethyl‐4H‐1,2,4‐triazol‐3‐yl)pyrimidine‐2,4(1*H*,3*H*)‐dione was accomplished by sequential formation of the 1,2,4‐triazole ring system from orotic acid hydrazide (Figure 2). In the first synthetic step, the terminal –NH_2_ group of the hydrazide selectively attacked ethyl isothiocyanate, furnishing the corresponding carbothioamide intermediate. The reaction was performed in a protic solvent with a relatively low boiling point under moderate thermal conditions. Since the product separated from the reaction mixture as a precipitate, it was readily isolated by simple filtration, avoiding any additional chromatographic treatment.

Base‐induced ring closure of the carbothioamide intermediate afforded the corresponding 1,2,4‐triazole‐3(2*H*)‐thione system attached to the pyrimidine‐2,4‐dione core. Following completion of the cyclization step, cooling and subsequent treatment with acetic acid caused precipitation of the desired mercaptotriazole products, which were isolated as light yellow solids. Owing to thione–thiol tautomerism [50], these compounds readily form thiolate anions in alkaline medium, accounting for their pronounced solubility in aqueous alkali and in polar aprotic media such as DMF and 1,4‐dioxane.

Modification of the sulfur atom was performed via S‐alkylation. In the presence of sodium hydroxide, the mercapto group was converted into the corresponding nucleophilic thiolate, which efficiently reacted with alkyl halides in propan‐2‐ol. The reaction conditions favored alkylation at sulfur, while alternative *N*‐alkylation pathways were minimized. The obtained derivatives were isolated as yellow to brown crystalline compounds or oily products. They were generally water‐insoluble but dissolved well in organic solvents and were purified by recrystallization from methanol; DMF was used for the preparation of analytical samples.

### 3.2. Biological Activity

Most of the remaining analogs demonstrated only marginal effects in the COX inhibition and DPPH tests under the applied conditions; hence, the discussion was focused on the most informative representatives (**13**, **14**, **17**, **19**, and **20**).

#### 3.2.1. Antiradical Activity

Among the various groups of antioxidants with different mechanisms of action, the most significant role is played by antiradical antioxidants—substances that interact with free radicals to form products that cannot continue oxidation chain reactions or reduce the reaction rate [51, 52]. The antiradical activity of the synthesized compounds, assessed using the DPPH free radical test, demonstrates high levels of activity (Table 1).

**Table 1:** Antiradical activity and absorption coefficients of 1,2,4‐triazole derivatives.

Compounds	Absorption coefficient, *A*	% antiradical activity
Control	0.4305	—
Ascorbic acid	0.2959	31.26
14	0.2334	39.62
20	0.1797	51.47
13	0.1574	58.75
19	0.1278	72.34
17	0.3728	24.81

According to the obtained data, ascorbic acid affords 31.26% inhibition of DPPH• under the experimental conditions, which is consistent with the expected activity of a classical low‐molecular‐weight antioxidant and may be considered an internal standard of the assay system. Against this background, Compound **17** exhibited the lowest antiradical activity among the tested derivatives (24.81%), which is slightly lower than that of the reference and indicates a limited ability of this structure to scavenge free radicals. In contrast, Compound **14** provides 39.62% inhibition of DPPH•, clearly exceeding the value observed for ascorbic acid and suggesting an enhancement of electron‐donating properties due to specific structural fragments.

More pronounced antiradical activity was observed for Compounds **20** and **13**, which showed 51.47% and 58.75% inhibition of DPPH•, respectively. These values can be classified as indicative of a moderately high antioxidant potential, reflecting efficient involvement of these molecules in proton/electron transfer processes in the applied model system. The most active member of the series was Compound **19**, which achieved 72.34% scavenging of DPPH• and thus substantially outperformed both ascorbic acid and the other derivatives. This allows Compound **19** to be regarded as the most promising candidate in terms of antioxidant action, capable of effectively quenching free radicals under in vitro conditions.

Overall, a clear gradient of increasing antiradical activity can be distinguished in the order **17** < ascorbic acid < **14** < **20** < **13** < **19**, which likely reflects the influence of the electron‐donating/electron‐withdrawing properties of the substituents and the presence of potential proton‐donating sites within the structures of the studied compounds. A comparative analysis with the COX‐2 inhibition data further supports the consideration of Compound **19** as a dual‐profile system, combining high antiradical potential with pronounced COX‐2 selectivity, making it an attractive target for more detailed biological investigations.

**Figure 3:**
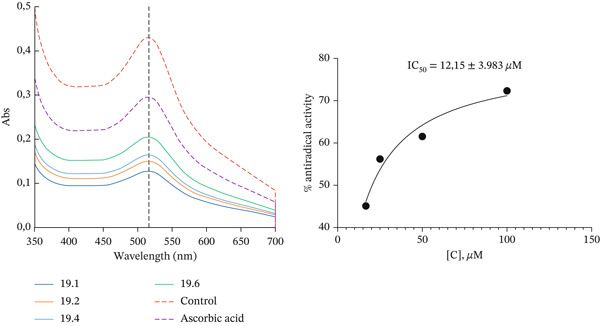
Study of the kinetic parameters of the reaction with 1,1‐diphenyl‐2‐picrylhydrazyl (DPPH) and calculation of the IC_50_ value.

Figure 3 shows the UV spectra of DPPH• radical solution (*λ*_max_ = 516 nm) in the presence of increasing concentrations of the tested compound (Curves **19.1**–**19.6**), as well as the control sample and ascorbic acid. An increase in compound concentration leads to a gradual decrease in absorbance at 516 nm, indicating a dose‐dependent reduction of DPPH• and a corresponding rise in antiradical activity. Based on the change in *A*_516_, the percentage of inhibition was calculated, and the concentration–response curve for antiradical activity was constructed (Figure 3).

Nonlinear regression of the concentration–response curve for radical scavenging activity yielded an IC_50_ value for the most active compound of 12.15 ± 3.98 *μ*M, which corresponds to approximately 3.60 *μ*g/mL, taking into account its molecular weight of 296 g/mol. According to the literature data [20], the IC_50_ of ascorbic acid is 41.25 *μ*g/mL; thus, the investigated compound exhibits approximately 11.5‐fold higher radical scavenging activity (lower IC_50_ value) than this reference antioxidant.

#### 3.2.2. In Vitro COX‐1/COX‐2 Inhibitory Profile and COX‐2 Selectivity

In vitro evaluation of cyclooxygenase inhibition revealed that Compounds **13**, **14**, **17**, **19**, and **20** display clearly distinct activity profiles toward COX‐2 and COX‐1 (Table 2). The highest COX‐2 inhibitory potency was observed for Compound **19**, with an IC_50_(COX‐2) value of 24.5 ± 0.9 *μ*M, falling into the subhundred micromolar range. Compound **14** also exhibited appreciable activity against COX‐2 (IC_50_ = 42.6 ± 0.01 *μ*M), whereas Compounds **20** (IC_50_ = 50.7 ± 0.03 *μ*M) and **13** (IC_50_ = 68.5 ± 0.06 *μ*M) showed somewhat lower, yet still satisfactory, levels of inhibition. The weakest affinity for COX‐2 within the series was found for Compound **17** (IC_50_ = 281.5 ± 0.37 *μ*M), indicating a relatively modest contribution of this molecule to selective COX‐2 blockade.

**Table 2:** In vitro inhibitory activity of synthesized compounds against COX‐1 and COX‐2 in comparison with ibuprofen.

Compound	IC_50_(COX‐2), *μ*М	IC_50_(COX‐1), *μ*М	*S* *I* = *I* *C*_50_(*C* *O* *X* − 1)/*I* *C*_50_(*C* *O* *X* − 2)	Inhibition profile
13	68.5 ± 0.06	92.0 ± 1.5	1.34	Modest COX‐2 preference
14	42.6 ± 0.01	1.48 ± 2.1	0.03	Pronounced COX‐1‐shifted
17	281.5 ± 0.37	83.4 ± 0.9	0.30	COX‐1‐shifted
19	24.5 ± 0.9	201.2 ± 0.15	8.21	Strong COX‐2 selectivity
20	50.7 ± 0.03	59.5 ± 1.0	1.17	Almost nonselective/weak COX‐2 bias
Ibuprofen	223	4.85	0.02	Classical NSAID, COX‐1‐shifted

Analysis of the COX‐1 data revealed even more pronounced differences between the compounds. SI values around 1–1.5 indicate only a modest isoform preference, whereas SI ≈ 8 reflects pronounced COX‐2 selectivity. Compound **14** proved to be a potent COX‐1 inhibitor (IC_50_ = 1.48 ± 2.1 *μ*M), which is almost an order of magnitude stronger than its effect on COX‐2 and indicates a clearly COX‐1‐biased profile. For Compounds **20** (IC_50_(COX − 1) = 59.5 ± 1.0 *μ*M) and **13** (IC_50_(COX − 1) = 92.0 ± 1.5 *μ*M), the IC_50_ values for COX‐1 are similar to or slightly higher than those for COX‐2, consistent with an almost nonselective or moderately COX‐2‐oriented pattern. In contrast, Compound **19** exhibits markedly weaker inhibition of COX‐1 (IC_50_ = 201.2 ± 0.15 *μ*M) while retaining high activity toward COX‐2, whereas Compound **17** shows moderate COX‐1 inhibition (IC_50_ = 83.4 ± 0.9 *μ*M) combined with very weak effects on COX‐2.

Calculation of the SI (SI = IC_50_(COX − 1)/IC_50_(COX − 2)) allowed a quantitative assessment of the inhibitory bias. For Compounds **20** and **13**, SI values of approximately 1.17 and 1.34, respectively, are in line with a modest preference for COX‐2 inhibition. For Compound **19**, SI reaches ~8.2, indicative of pronounced COX‐2 selectivity combined with high absolute potency toward this isoform. In contrast, Compounds **14** (SI ≈ 0.03) and **17** (SI ≈ 0.30) preferentially inhibit COX‐1, displaying a profile typical of classical NSAIDs with COX‐1 bias.

Comparison with the reference drug ibuprofen (IC_50_(COX − 2) = 223 *μ*M, IC_50_(COX − 1) = 4.85 *μ*M, SI ≈ 0.02) demonstrates that Compounds **14**, **20**, **13**, and, most notably, **19** are substantially more potent COX‐2 inhibitors (approximately 3–9‐fold lower IC_50_ values), whereas for COX‐1, only Compound **14** approaches the reference NSAID in terms of inhibitory strength. From the perspective of selective COX‐2 inhibition, Compound **19** appears to be the most promising candidate, combining the lowest IC_50_ for COX‐2 with a high SI and markedly weaker action on COX‐1 compared to ibuprofen. Thus, within the investigated series, Compound **19** may be regarded as a lead candidate for further preclinical evaluation of selective COX‐2‐modulating activity, while Compounds **20** and **13** form a group of moderately COX‐2 selective inhibitors, and Compounds **14** and **17** exhibit a COX‐1‐shifted profile reminiscent of classical NSAIDs.

### 3.3. Quantum Chemical Analysis of the Antioxidant Mechanism

Within the quantum chemical part of the study, 20 hybrid S‐heterocyclic derivatives (Compounds **1**–**20**) were analyzed. Inspection of the calculated *Δ* *G*_298_ values (Table 3) shows that for most of the systems examined (Compounds **1–4**, **6–9**, **13**, **16**, and **18**–**20**), the SPLET_NH_ pathway is predicted to be the dominant mechanism in both water and methanol.

**Table 3:** Calculated *Δ* *G*_298_ values (kJ/mol) for the first step of the dominant antioxidant mechanism of hybrid Compounds **1–20** in water and methanol.

No.	Preferred mechanism in H_2_O	*Δ* *G*_298_(H_2_O), kJ/mol	Preferred mechanism in MeOH	*Δ* *G*_298_(MeOH), kJ/mol	BDE_NH_ (H_2_O), kJ/mol	BDE_NH_ (MeOH), kJ/mol
1	SPLET_NH_	95.8	SPLET_NH_	145.4	447.49	454.15
2	SPLET_NH_	93.5	SPLET_NH_	136.7	453.08	457.39
3	SPLET_NH_	94.8	SPLET_NH_	138.2	452.98	457.16
4	SPLET_NH_	95.0	SPLET_NH_	139.2	452.47	456.85
5	SPLET_NH_	−400.4	SPLET_NH_	139.4	451.88	456.28
6	SPLET_NH_	94.2	SPLET_NH_	153.5	451.85	448.39
7	SPLET_NH_	95.0	SPLET_NH_	138.1	452.43	—
8	SPLET_NH_	101.7	SPLET_NH_	154.0	441.21	448.41
9	SPLET_NH_	100.7	SPLET_NH_	139.4	441.47	456.34
10	SPLET_NH_	101.8	SET–PT	−463.8	444.52	450.49
11	HAT_NH_	−544.8	HAT_NH_	−534.0	440.87	448.26
13	SPLET_NH_	95.1	SPLET_NH_	136.9	453.17	457.50
15	SPLET_NH_	92.1	—	—	452.85	—
16	SPLET_NH_	95.8	SPLET_NH_	136.7	451.67	456.70
17	SPLET_NH_	92.7	HAT_NH_	−201.6	452.89	457.24
18	SPLET_NH_	101.9	SPLET_NH_	153.2	448.51	455.42
19	SPLET_NH_	91.4	SPLET_NH_	134.6	451.61	458.61
20	SPLET_NH_	92.5	SPLET_NH_	135.4	452.01	458.06

The calculated *Δ* *G*_298_ values for the first step of the prevailing antioxidant mechanism of Compounds **1–20** in water and methanol are summarized in Table 3. Note that negative *Δ* *G*_298_ values indicate an extremely favorable (strongly exergonic) course of the corresponding elementary process under the given conditions.

In aqueous solution, the first step of the SPLET_NH_ mechanism is characterized by moderately endergonic *Δ* *G*_298_ values of approximately 91–102 kJ/mol, which is consistent with the high proton‐donating ability of water and efficient stabilization of the anionic forms. In methanol, the same compounds display somewhat higher *Δ* *G*_298_ values (≈135–154 kJ/mol), yet SPLET_NH_ remains the most thermodynamically favorable pathway. The lowest *Δ* *G* for the first SPLET_NH_ step in water was obtained for Compounds **19** (*Δ* *G*_298_ = 91.4 kJ/mol) and **15** (*Δ* *G*_298_ = 92.1 kJ/mol), whereas in methanolic solution, Compounds **19** and **20** stand out with *Δ* *G*_298_ values of 134.6 and 135.4 kJ/mol, respectively, allowing them to be regarded as the most promising proton/electron‐donor systems in the series.

A separate group is formed by compounds for which the calculations indicate an extremely favorable (*Δ* *G*_298_ < 0) course of alternative pathways. Thus, for Compound **5**, the first SPLET_NH_ step in water is spontaneous (*Δ* *G*_298_ = −400.4 kJ/mol), whereas for Compound **10** in methanol, the SET–PT mechanism dominates with *Δ* *G*_298_ = −463.8 kJ/mol. For Compound **11**, the HAT_NH_ mechanism is predicted to be the most favorable in both solvents (*Δ* *G*_298_ = −544.8 kJ/mol in H_2_O and −534.0 kJ/mol in MeOH), indicating an exceptionally high propensity of this system for direct HAT. An interesting situation is observed for Compound **17**: in water, SPLET_NH_ predominates (*Δ* *G*_298_ = 92.7 kJ/mol), whereas in methanol, HAT_NH_ becomes more favorable (*Δ* *G*_298_ = −201.6 kJ/mol), highlighting the pronounced influence of solvent nature on the shift of the dominant mechanism.

Overall, the calculations demonstrate that for the majority of the hybrid S‐heterocyclic derivatives considered in this work, the SPLET pathway represents the most probable route of antioxidant action, whereas HAT and SET–PT act as alternative or dominant mechanisms only for a few structurally specific compounds (**5**, **10**, **11**, and **17**). This sensitivity of the operative mechanism to the structure of the radical‐forming fragment and to the solvent environment correlates well with the presence of acidic sites in the molecules and the degree of charge delocalization and will be discussed in more detail in the context of the experimental antioxidant data.

In addition, N–H bond dissociation enthalpies BDE_NH_ were computed for the radical forms of all systems studied, as these parameters are traditionally regarded as descriptors of the propensity to follow an HAT mechanism. The calculated BDE_NH_ values in water lie in the range 440.9–453.2 kJ/mol, whereas in methanol, they fall between 448.3 and 458.6 kJ/mol. The lowest BDE_NH_ value is observed for Compound **11** in water (440.9 kJ/mol), which correlates well with the strongly exergonic first HAT_NH_ step in this system (*Δ* *G*_298_ < 0) and underscores its pronounced ability to undergo direct HAT. In contrast, for Compounds **19** and **20** in methanol, the BDE_NH_ values are among the highest in the series (458.6 and 458.1 kJ/mol, respectively), further indicating that HAT is energetically disfavored for these structures and is consistent with the dominance of the SPLET_NH_ pathway as their main antioxidant mechanism. Taken together, the BDE_NH_ data support the conclusion that the HAT mechanism can play a leading role only for selected compounds with the weakest N–H bonds (primarily **11** and to a lesser extent **8** and **9**), whereas for most members of the series, the SPLET mechanism prevails, as inferred above from the analysis of *Δ* *G*_298_.

Among Compounds **1–20**, particular attention is paid to Derivatives **13**, **14**, **17**, **19**, and **20**, for which the quantum chemical analysis allows us to delineate characteristic mechanistic features of their antioxidant behavior. Compounds **19** and **20** showed the lowest *Δ* *G*_298_ values for the first SPLET_NH_ step in both water and methanol and can therefore be regarded as “benchmark” representatives of a SPLET‐controlled pathway with highly favorable sequential proton dissociation and electron transfer. Compound **13** also belongs to the group of SPLET‐dominated systems, although its *Δ* *G*_298_ values are somewhat higher than those for **19** and **20**, indicating a more moderate yet clearly expressed tendency to follow the SPLET mechanism. Compound **17** proved to be mechanistically “switchable”: in water, it behaves similarly to most derivatives in the series, with SPLET_NH_ as the preferred pathway, whereas in methanol, HAT_NH_ dominates and is strongly exergonic, pointing to the decisive role of solvent nature in governing the balance between proton‐dissociative and radical‐type routes.

### 3.4. Molecular Docking

For molecular docking, we selected the crystal structures of the enzymes 1EQG, 3LN1, 3N8Z, 5F19, and 5KIR, as they represent the classical targets of NSAIDs, namely, the COX‐1 and COX‐2 isoforms of cyclooxygenase, which are directly involved in prostaglandin biosynthesis and the development of inflammation and pain. Structures 1EQG and 3N8Z correspond to COX‐1 cocrystallized with ibuprofen and flurbiprofen, respectively, whereas 3LN1, 5F19, and 5KIR describe COX‐2 in complex with selective inhibitors (celecoxib, aspirin‐acetylated COX‐2, and rofecoxib). These models provide reliable reference binding modes for docking validation and comparison with reference NSAIDs. Owing to their high resolution and cocrystallized ligands, these PDB structures are widely used in the structure‐based design of COX inhibitors and allow a meaningful interpretation of predicted binding energies and interaction patterns.

The molecular docking results (Table 4) indicate that the series of Derivatives **1–20** displays a broad range of affinities toward the target proteins 1EQG, 3LN1, 3N8Z, 5F19, and 5KIR, which makes it possible to assess both overall activity and potential selectivity. In general, all members of the series exhibit energetically favorable binding (approximately −5.8 to −8.9 kcal/mol), although the strength of the interactions varies markedly between individual ligands.

**Table 4:** Molecular docking interactions and binding affinities for selected compounds with target proteins.

Ligand	1EQG	3LN1	3N8Z	5F19	5KIR
Ibuprofen	−7.11238	−7.1921	−7.13912	−7.76598	−7.8315
1	−7.2665	−7.1788	−7.50632	−8.06171	−7.1879
2	−6.46964	−6.27539	−6.57306	−6.22146	−6.97758
3	−6.35389	−6.67755	−6.58479	−5.90934	−5.85554
4	−6.45158	−5.24346	−5.5816	−6.9477	−6.6368
5	−6.7572	−7.27137	−7.10346	−7.59146	−7.78267
6	−6.744	−7.50258	−7.34848	−7.76391	−6.80459
7	−7.19972	−6.96776	−6.47617	−8.0278	−6.90655
8	−7.26595	−7.18385	−6.73295	−7.86492	−7.21876
9	−7.12384	−7.46131	−6.83524	−7.53975	−6.98799
10	−7.55107	−7.53147	−6.78421	−7.39814	−7.35592
11	−7.12	−6.4531	−6.67298	−7.26371	−6.86263
12	−7.31403	−6.23207	−6.72379	−6.39513	−6.23189
13	−7.29439	−7.74872	−7.32243	−8.89253	−7.9325
14	−8.51364	−8.74469	−7.71973	−8.54717	−7.58845
15	−6.72383	−7.60346	−6.79348	−6.97489	−7.78384
16	−7.05032	−7.70613	−7.54225	−7.83638	−7.62551
17	−7.5231	−7.99897	−7.66114	−8.39703	−7.67018
18	−7.16896	−6.78025	−6.89417	−7.56482	−7.84844
19	−7.15069	−7.61797	−7.54279	−8.87408	−8.40951
20	−8.01948	−8.64651	−8.22294	−8.39954	−8.39451

The highest affinity in most cases was observed for Compound **14**, which consistently ranks among the top binders in terms of docking score. In particular, it is one of the best ligands for 1EQG (−8.51 kcal/mol), 3LN1 (−8.74 kcal/mol), and 5F19 (−8.55 kcal/mol). Likewise, Compounds **20**, **17**, and **19** also showed high affinity; among them, **20** and **19** are distinguished by especially low binding energies for 3LN1, 3N8Z, 5F19, and 5KIR, indicating a broadly compatible interaction pattern with several active sites.

Interestingly, target 5F19 provides some of the deepest (most favorable) binding energies in the series (down to −8.89 kcal/mol for Compound **13** and −8.87 kcal/mol for Compound **19**), which may reflect an increased sensitivity of this model to the structural motifs present in the studied derivatives. In contrast, one of the COX‐2 models is characterized by somewhat higher (less negative) docking scores for most ligands, although certain compounds (**17**, **10**, and **20**) still display appreciably better affinity toward this receptor.

Comparison of the weaker binders revealed that Compounds **3** and **4** show the lowest affinity for most targets, with docking scores typically in the range −5.2 to −6.9 kcal/mol. This suggests a less favorable orientation in the active site and a possible mismatch with key pharmacophoric requirements.

In contrast, the profiles of Compounds **11**, **5**, and **6** can be regarded as moderately active: they provide intermediate binding energies (approximately −6.4 to −7.8 kcal/mol) and exhibit a relatively balanced activity across different protein targets without pronounced selectivity. This makes them attractive scaffold candidates for further structural optimization.

Taken together, two main groups can be distinguished within the **1–20** series:

1. *Potent “universal” inhibitors*—Compounds **14**, **20**, **17**, and **19**, which consistently show the most favorable binding energies for most proteins and can be considered primary candidates for more detailed investigation.
2. *Low-affinity compounds*—Compounds **3** and **4**, which likely require substantial structural modification or appear poorly matched to the geometry of the selected active sites.

Overall, the interaction patterns indicate that proteins 1EQG, 3LN1, 5F19, and 5KIR are the most sensitive to structural variation within the **1–20** series and best discriminate between strong and weak ligands. At the same time, Compound **12** exhibits a somewhat distinct binding profile, which may point to an alternative recognition mode of the active site for certain members of the series.

Based on comparative molecular docking of the **1–20** derivative series against a panel of enzyme targets (3N8Z, 3LN1, 5KIR, 5F19, and 1EQG), pronounced differences in the affinity of individual compounds for the active sites were observed. The compiled matrix of best docking scores (*best score*) showed that Compounds **15**–**17** and **19** consistently exhibit the lowest (i.e., most favorable) binding energies across most models, indicating high overall affinity and a potential pan‐inhibitor profile.

Analysis of the mean rank values (*Mean_rank*) revealed Compound **16** as the consensus leader within the series: it consistently ranks among the top three ligands for most enzymes, including 3N8Z and 5KIR. Compounds **15** and **17** occupy the second and third positions, respectively, and are characterized by low average ranks and minimal SDs of the docking scores across targets. This pattern suggests highly robust interactions and the absence of narrow target selectivity.

In contrast, Compounds **3**–**5** and **12** display increased variability in binding energies (*S* *e* *l* *e* *c* *t* *i* *v* *i* *t* *y*_*r* *a* *n* *g* *e* > 2.5–3.0 kcal/mol), pointing to a more selective interaction profile. These ligands show the highest affinity toward 5F19 and 1EQG, whereas their activity against the remaining proteins is noticeably weaker. Such behavior is likely associated with a specific orientation in the catalytic pocket and/or differences in the distribution of donor–acceptor centers.

Correlation analysis between the targets demonstrated that the pairs (5KIR–3LN1) exhibit high positive correlation coefficients (*r* ≈ 0.82–0.90), consistent with related active‐site architectures and similar requirements for the spatial and electronic properties of ligands. In contrast, 1EQG shows weak or even negative correlations with the other proteins (*r* ≈ −0.2 to 0.3), which supports its unique pharmacophoric profile and explains the pronounced selectivity of certain derivatives toward this particular target.

Overall, the mean and median rank values indicate that Compounds **15–17** are the most promising universal inhibitors, combining high affinity with a balanced selectivity profile. At the same time, Compounds **3**, **4**, and **12** may serve as selective prototypes for further optimization toward specific targets, especially 5F19 and 1EQG.

Thus, two main trends can be distinguished within the **1**–**20** series:

1. *Consensus-type activity*, characteristic of Compounds **15–17**, which display consistently low binding energies across the majority of enzymes.
2. *Target selectivity*, characteristic of Compounds **3**, **4**, and **12**, which show clear advantages in binding to particular proteins but markedly weaker interactions with others.

The strong positive correlation between activities at 3N8Z and 6N2W suggests that these enzymes can be considered interchangeable markers in virtual screening campaigns. Conversely, the low or negative correlation coefficients between 1EQG and the remaining targets confirm its specific mode of ligand recognition, which warrants separate, more detailed investigation.

The energy‐ and rank‐based characteristics provide an integrated view of the affinity and selectivity of Derivatives **1–20** toward the panel of enzyme targets but do not fully describe the spatial arrangement of ligands within the active sites. Therefore, as a next step, we performed a structural analysis of noncovalent contacts in representative complexes formed by the lead Compounds **12**–**14**, **19**, and **20** using PLIP. This enabled quantitative characterization of the contributions of hydrogen bonds, hydrophobic contacts, potential *π*‐interactions, and other types of noncovalent forces to complex stabilization with 1EQG, 3LN1, 3N8Z, 5F19, 5TZ1, and 5KIR. The summarized results of this interaction analysis (Table 5) allowed us to relate docking energies to specific pharmacophoric binding patterns and to identify key amino acid residues responsible for the high affinity of the best‐performing ligands.

**Table 5:** Molecular docking interactions and binding affinity of selected compounds with target proteins.

Protein ID	Ligand	Affinity (kcal/mol)	Hydrogen bonds, *n* (key residues)	Hydrophobic contacts, *n* (key residues)
5F19	13	−8.89	7 (ALA199(A), HIS207(A), ASN382(A), TYR385(A), TRP387(A), HIS388(A))	1 (TYR385(A))
1EQG	14	−8.51	6 (CYS47(B), ASP135(B), GLN327(A))	2 (TYR39(B), PRO156(B))
3LN1	14	−8.74	10 (ASN19(D), CYS21(D), ASN24(D), CYS26(D), PRO140(D), ALA142(D), GLN447(D), GLU451(D))	1 (PRO139(D))
5KIR	19	−8.41	11 (HIS39(B), CYS41(B), ARG44(B), GLY45(B), CYS47(B), TYR130(B), GLN461(B), ARG469(B))	0
3N8Z	20	−8.22	7 (SER126(A), GLN370(A), GLN372(A), GLU543(B))	0

According to the PLIP analysis (Figure 4), Compound **14** forms a dense network of six hydrogen bonds in its complex with protein 1EQG, involving polar and charged residues CYS47(B), ASP135(B), and GLN327(A). These hydrogen bonds are established predominantly through the heteroatoms of the triazole and pyrimidine fragments, which provide an “anchoring” of the ligand in the central region of the active site. Additional stabilization of the complex is ensured by two hydrophobic contacts with the aromatic residues TYR39(B) and PRO156(B), creating a hydrophobic environment around the aromatic core of Compound 14. This combination of multiple hydrogen bonds with a localized hydrophobic pocket correlates well with the high affinity of the compound for 1EQG, as evident from both the 2D interaction map and the 3D visualization of the complex (Figure 4).

Figure 4Visualization of molecular docking results showing the interactions between Compound **14** and prostaglandin H(2) synthase‐1 (PDB ID: 1EQG) in both (a) 3D and (b) 2D views.

**Figure:**
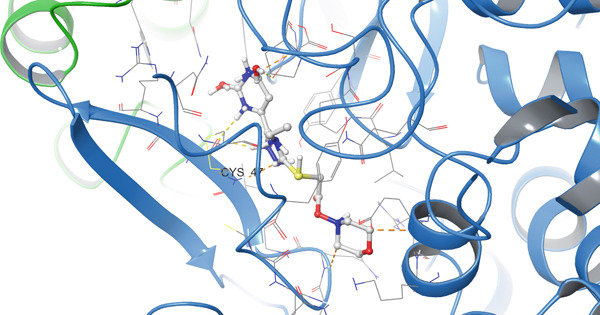
(a)

**Figure:**
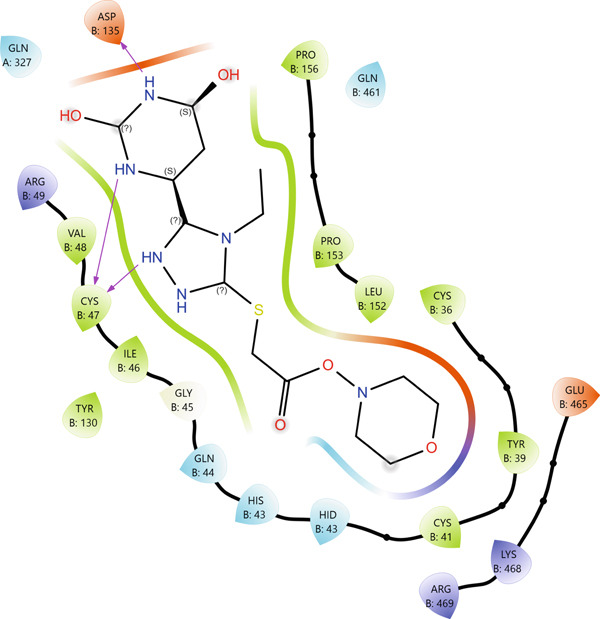
(b)

In the case of COX‐2 (PDB ID: 3LN1), Compound **14** exhibits an even more extensive interaction network. PLIP analysis revealed 10 hydrogen bonds involving residues ASN19(D), CYS21(D), ASN24(D), CYS26(D), PRO140(D), ALA142(D), GLN447(D), and GLU451(D). Thus, the ligand simultaneously engages both the *N*‐terminal region of the active site (ASN/CYS at Positions 19–26) and deeper residues (GLN447 and GLU451), forming an extended array of donor–acceptor interactions along the binding channel. A single hydrophobic contact with PRO139(D) further stabilizes the position of the aromatic fragment, reducing the rotational freedom of the ligand. In the 2D/3D interaction maps (Figure 5), the 3LN1–**14** complex appears “wrapped” by polar and weakly acidic residues, which is fully consistent with its favorable docking scores and accounts for the high rank of this compound at this target.

Figure 5Visualization of molecular docking results showing the interactions between Compound **14** and cyclooxygenase‐2 (PDB ID: 3LN1) in both (a) 3D and (b) 2D views.

**Figure:**
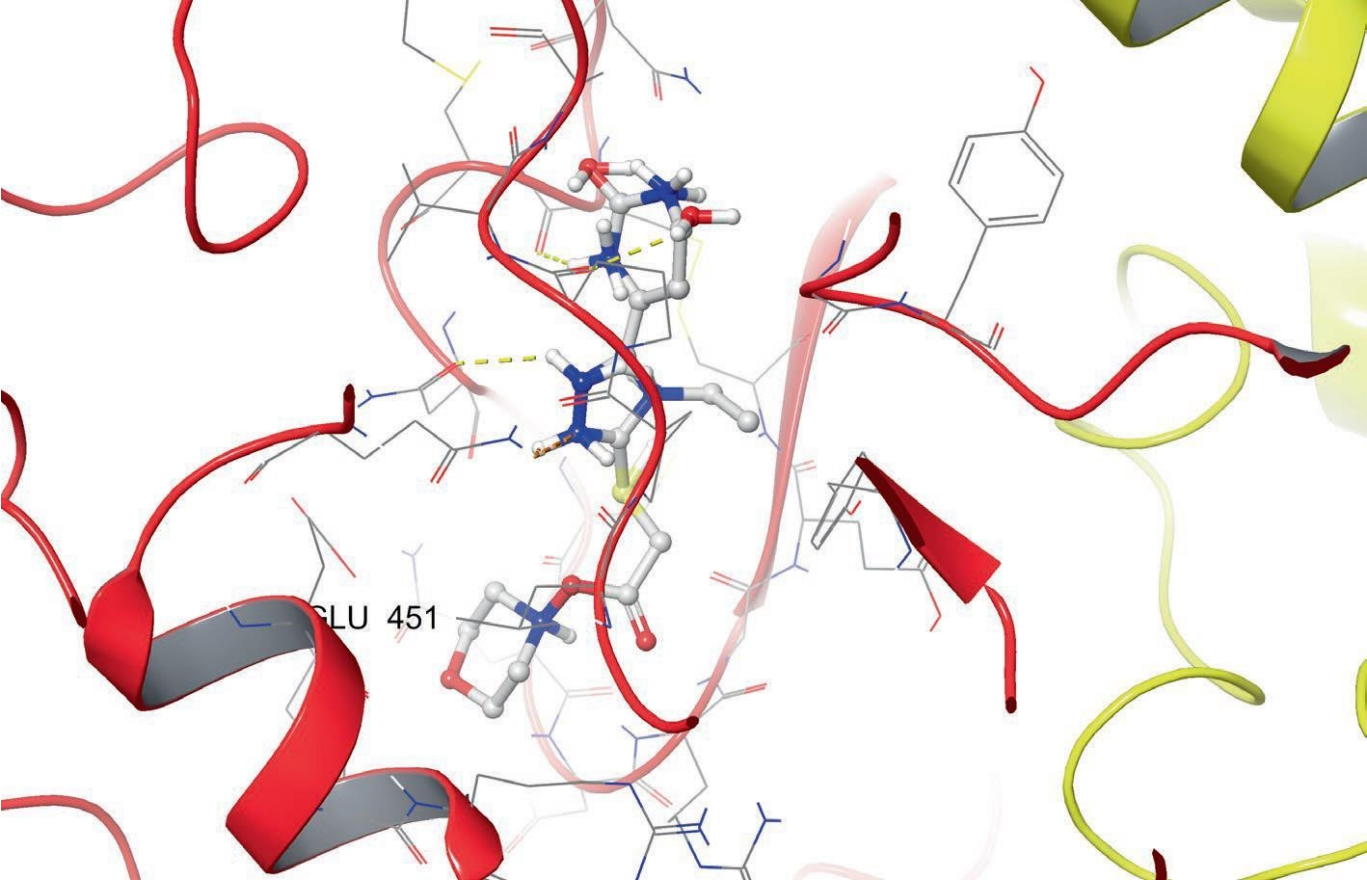
(a)

**Figure:**
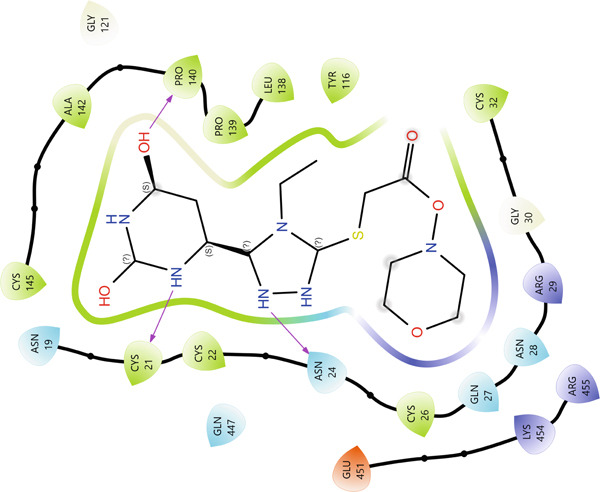
(b)

In the active site of enzyme 3N8Z, Compound **20** forms seven hydrogen bonds, with SER126(A), GLN370(A), GLN372(A), and GLU543(B) acting as the main donors/acceptors. These residues build a polar “belt” around the pharmacophoric portion of Compound **20**, thereby locking the ligand in a narrow pocket through multiple contacts with its oxygen‐ and nitrogen‐containing fragments. According to PLIP, hydrophobic interactions in this complex are virtually absent, indicating a predominantly electrostatic binding mode. In the 2D interaction map (Figure 6), this is visualized as a dense “framing” of the ligand by polar residues, whereas the 3D view clearly shows the orientation of Compound **20** arranged to maximize the hydrogen bonding potential of its heterocyclic system.

Figure 6Visualization of molecular docking results showing the interactions between Compound **20** and cyclooxygenase‐1 (PDB ID: 3N8Z) in both (a) 3D and (b) 2D views.

**Figure:**
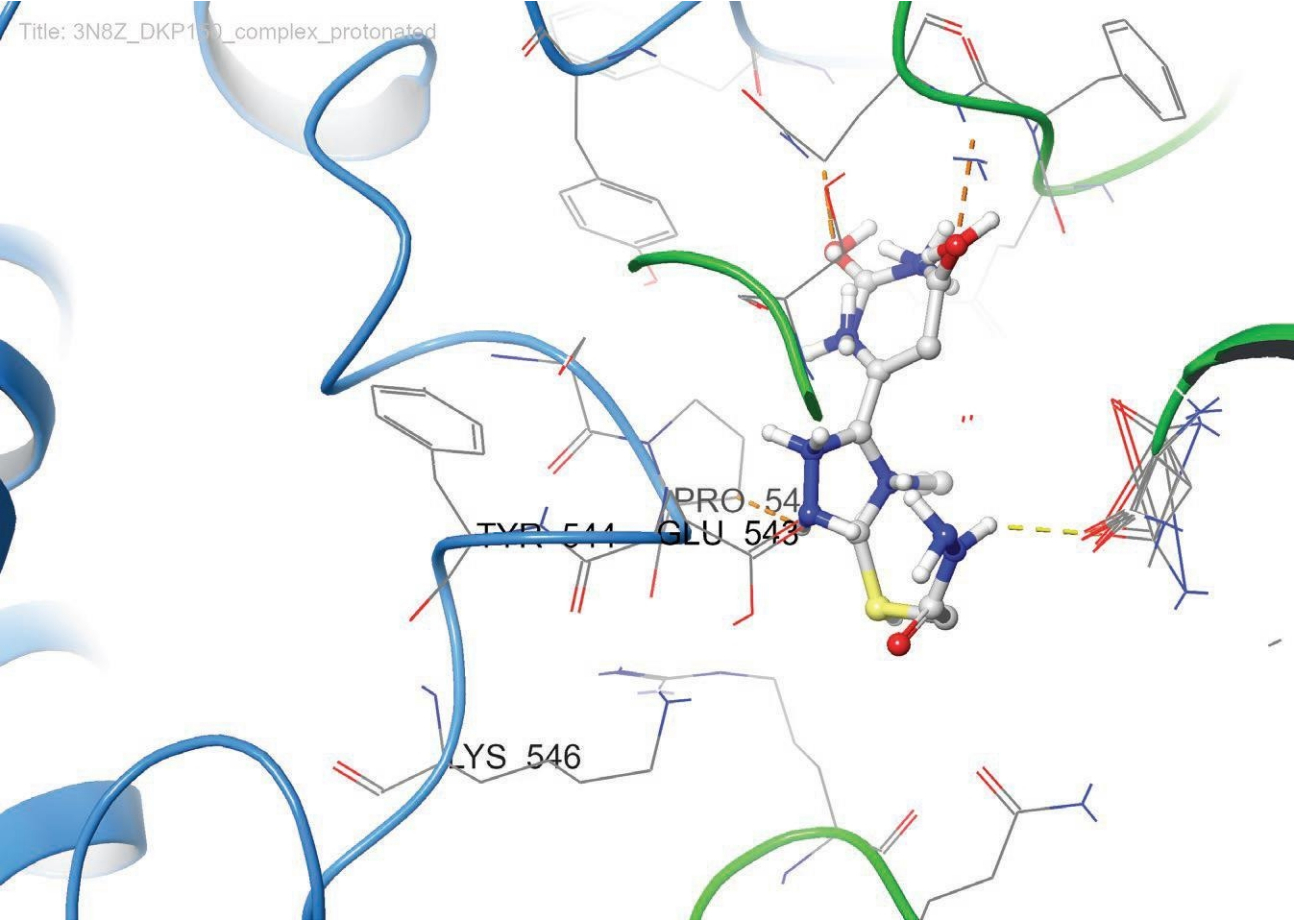
(a)

**Figure:**
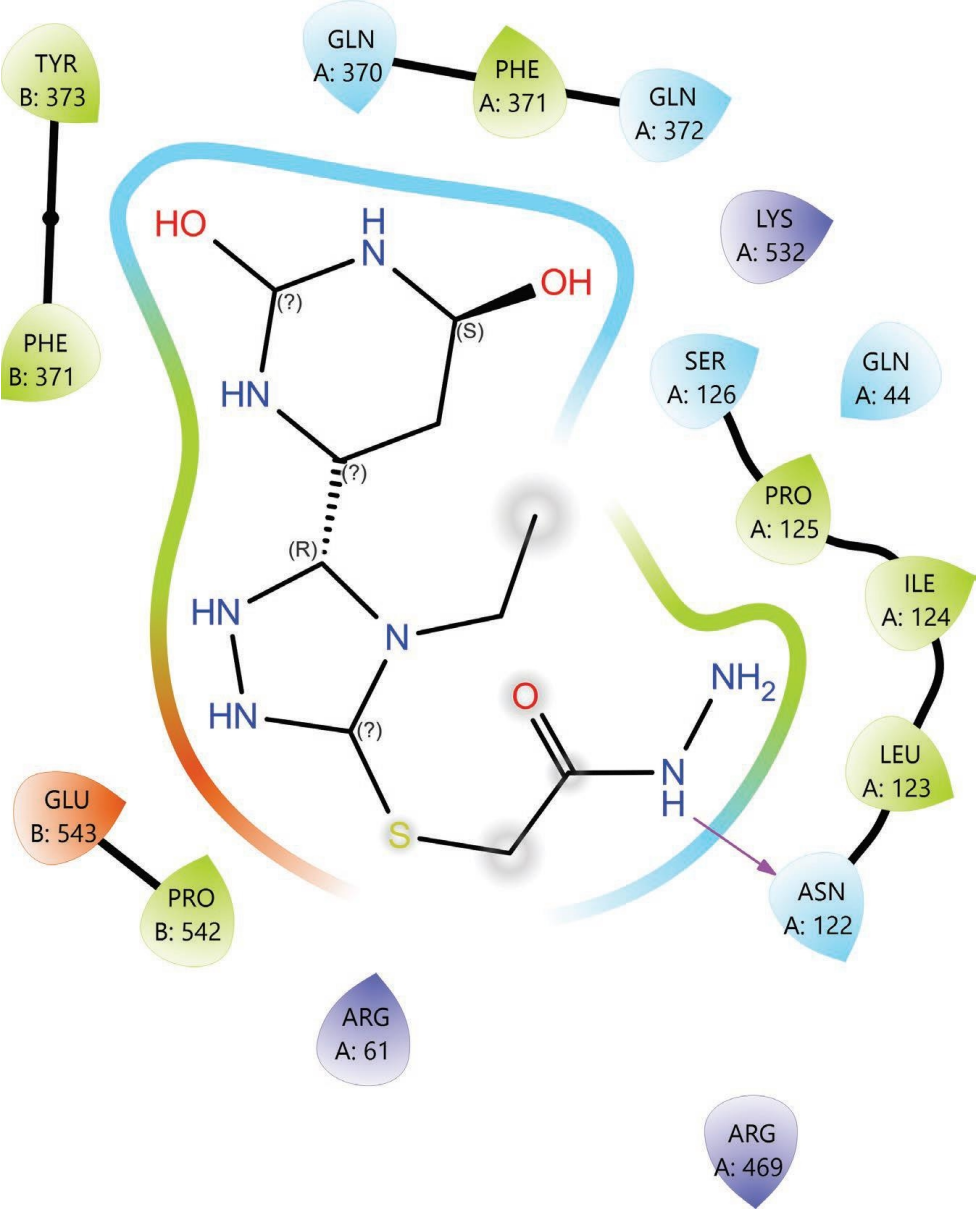
(b)

For Compound **13** in complex with protein 5F19, seven hydrogen bonds were identified with residues ALA199(A), HIS207(A), ASN382(A), TYR385(A), TRP387(A), and HIS388(A), all located within the catalytic site region. Particularly important are the contacts with the aromatic residues TYR385(A) and TRP387(A), as well as HIS388(A), which are frequently implicated in catalytic activity and ligand recognition in this class of enzymes. In addition, Compound **13** forms a single hydrophobic contact with TYR385(A), indicating a combined hydrogen bonding and hydrophobic anchoring of the ligand within the active site.

In the 2D interaction map (Figure 7), Compound **13** appears to be “sandwiched” between several aromatic residues, while the 3D model reveals a favorable *π*‐oriented arrangement with TYR/TRP. Although this interaction is not classified by PLIP as a distinct *π*–*π* stacking event, it clearly contributes to additional stabilization of the complex.

Figure 7Visualization of molecular docking results showing the interactions between Compound **13** and human cyclooxygenase‐2 (PDB ID: 5F19) in both (a) 3D and (b) 2D views.

**Figure:**
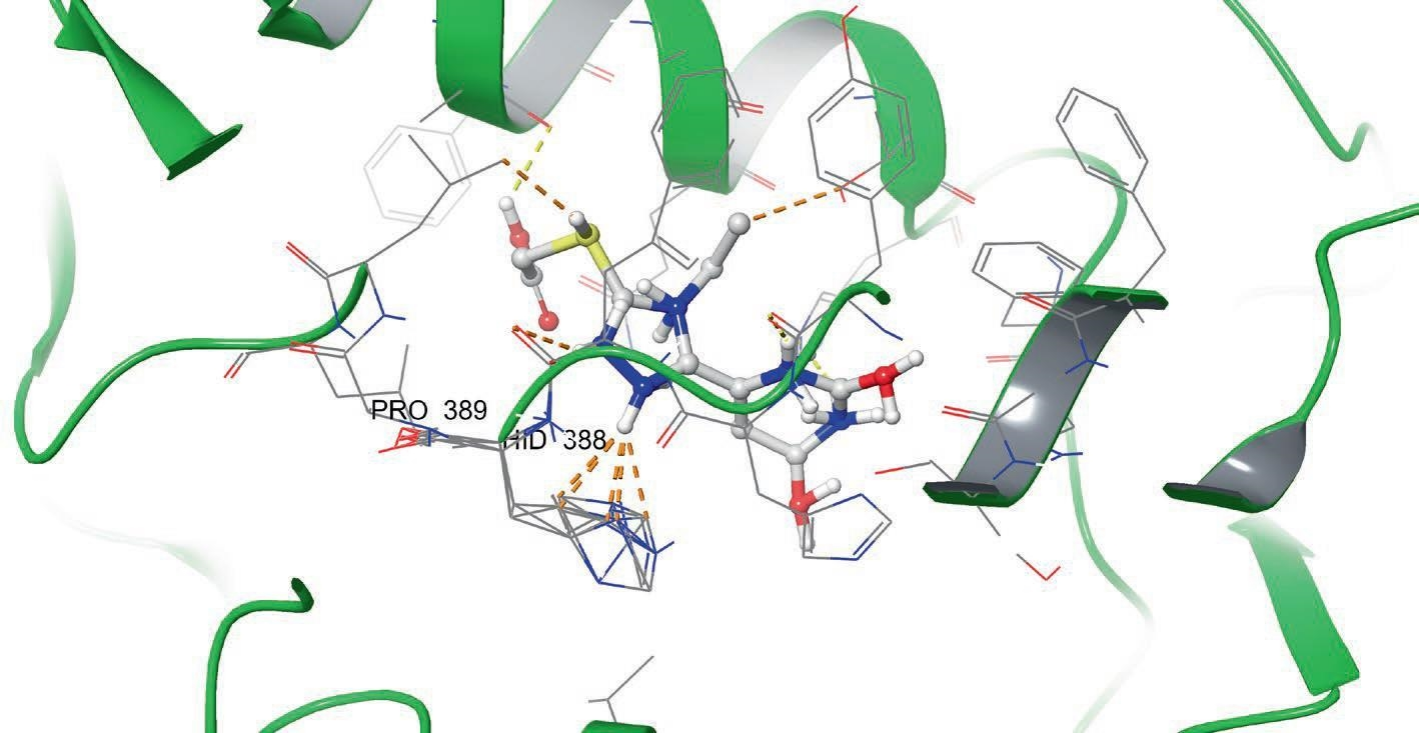
(a)

**Figure:**
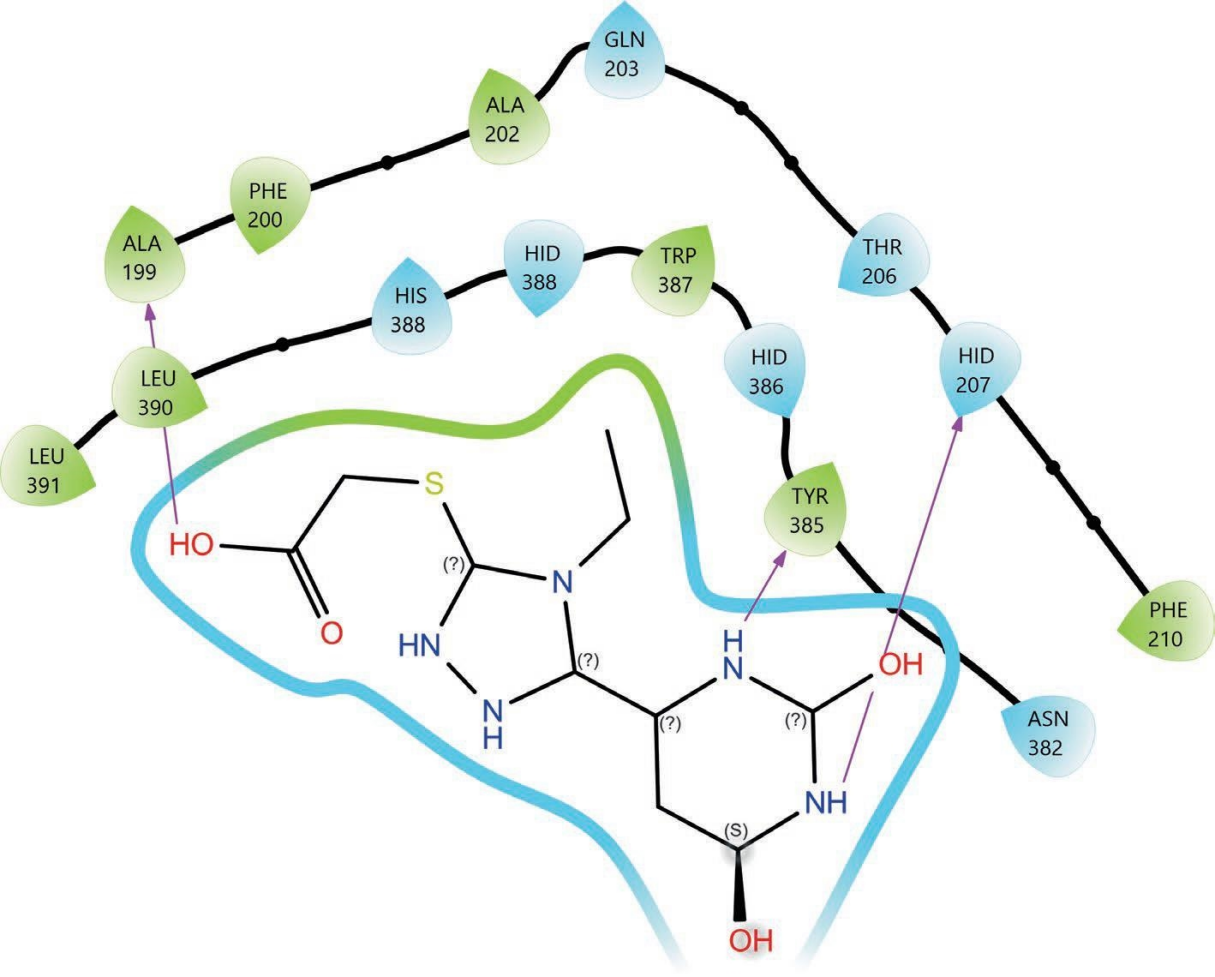
(b)

In the case of protein 5KIR, Compound **19** displays the most extensive network of polar contacts among the complexes examined: PLIP identified 11 hydrogen bonds involving residues HIS39(B), CYS41(B), ARG44(B), GLY45(B), CYS47(B), TYR130(B), GLN461(B), and ARG469(B). These amino acids form an elongated “corridor” along which the ligand is aligned, providing multitopic anchoring of Compound **19** and minimizing its conformational flexibility in the bound state. Hydrophobic interactions in this complex are much less pronounced and were not highlighted by PLIP as a distinct class, indicating that hydrogen bonding and electrostatic contributions dominate the binding mode. The 2D and 3D visualizations (Figure 8) clearly show the elongated binding pose of **19** along the active cleft, enabling simultaneous engagement with several key functional residues, which is fully consistent with the high affinity of this compound for 5KIR.

Figure 8Visualization of molecular docking results showing the interactions between Compound **19** and human cyclooxygenase‐2 (PDB ID: 5KIR) in both (a) 3D and (b) 2D views.

**Figure:**
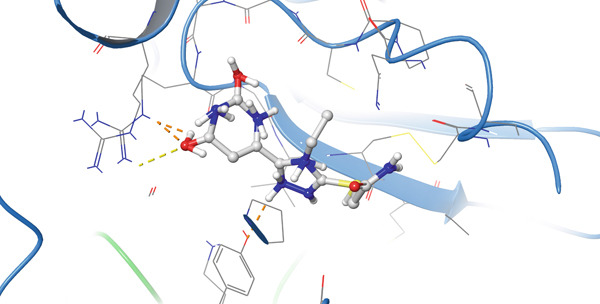
(a)

**Figure:**
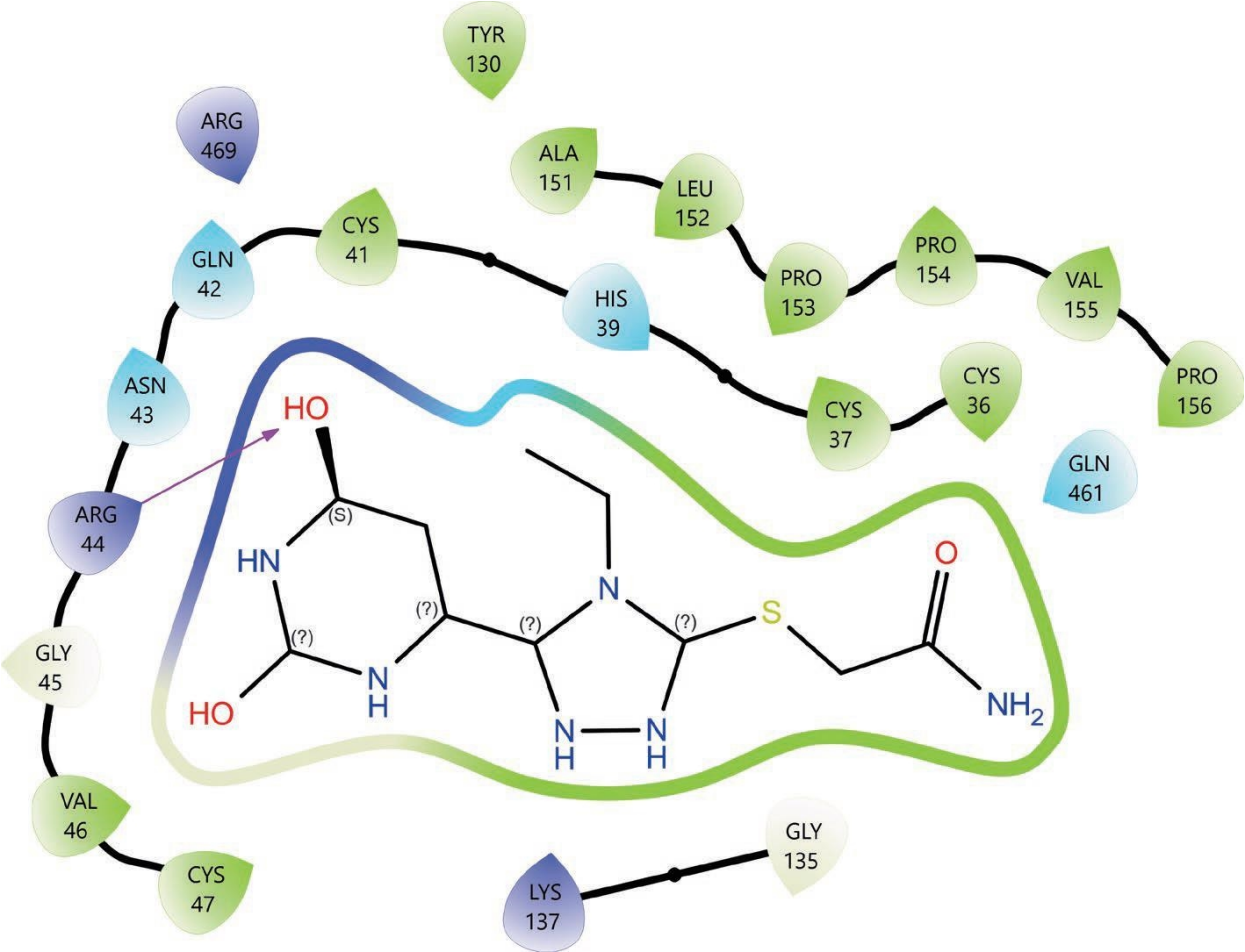
(b)

Importantly, the terminal carboxamide fragment of Compound **19** (S–CH_2_–CONH_2_) is oriented toward the COX‐2‐specific secondary side pocket and contributes to the preferential COX‐2 binding. This side pocket is structurally accessible in COX‐2 (owing to the smaller Val523 residue compared with Ile523 in COX‐1), providing additional volume for polar substituents and enabling extra hydrogen bonding opportunities. Therefore, the extended, hydrogen bond–dominated pose observed for **19** can be rationalized by its ability to project the polar tail into this COX‐2 side region, which is consistent with the experimentally observed COX‐2 preference.

### 3.5. ADME Profiling

In silico ADME properties of the S‐alkyl derivatives of 6‐(5‐mercapto‐4‐ethyl‐4*H*‐1,2,4‐triazol‐3‐yl)pyrimidine‐2,4(1*H*,3*H*)‐dione were evaluated using the SwissADME web tool, which predicts key pharmacokinetic parameters, classical “drug‐likeness” filters, and medicinal chemistry alerts for small molecules.

For the whole set, all molecules comply with the molecular‐weight criterion (239–384 Da) and show moderate consensus log *P* values (from slightly negative to ≈3.7), indicating a generally balanced lipophilicity. The majority of compounds are predicted as “very soluble” or “soluble” according to the ESOL and Silicos‐IT models, which is consistent with the presence of multiple heteroatoms and ionizable centers in the triazole–pyrimidinedione scaffold. At the same time, most of the more polar S‐acyl and S‐carbamoylmethyl derivatives (**13**, **14**, **17**, **19**, and **20**) exhibit TPSA values above 150 Å^2^ and fail the Veber/Egan criteria, which is reflected in the prediction of low GI absorption despite good solubility. In contrast, earlier S‐alkyl analogs with less polar side chains (**3**–**12**) combine TPSA ≈ 121–135 Å^2^ with higher lipophilicity and are predicted to have high GI absorption, illustrating a clear structure–ADME trade‐off between polarity/solubility and passive permeability within the series.

**Figure 9:**
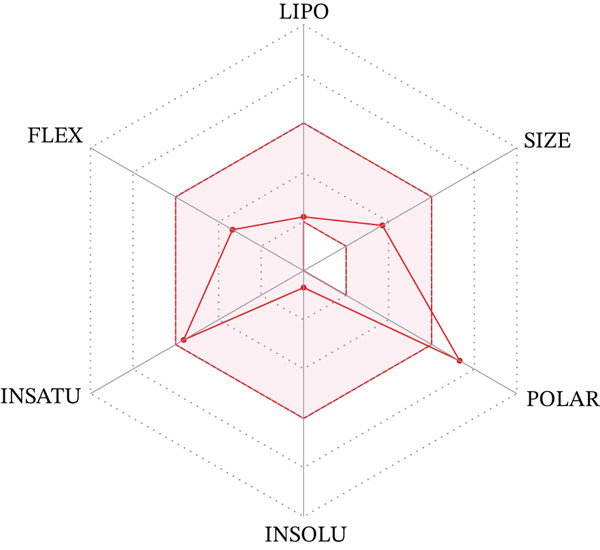
Radar of bioavailability of Compound **19**.

The bioavailability radar of Compound **19** (Figure 9) demonstrates an overall favorable physicochemical profile with a single pronounced deviation in the POLAR dimension. In the SwissADME bioavailability radar, the six petals summarize lipophilicity (LIPO), size (SIZE), polarity (POLAR, largely driven by TPSA), solubility (INSOLU), saturation (INSATU), and flexibility (FLEX), and the optimal oral drug‐like space is shown in pink. Compound **19** bears a terminal carboxamide fragment (S–CH_2_–CONH_2_) attached to the triazole sulfur, which increases the number of hydrogen‐bond donors and acceptors (3 and 5, respectively) and leads to a high TPSA of 164.8 Å^2^; consequently, the POLAR petal extends beyond the optimal range (TPSA typically ≤ 130 Å^2^) that favors passive GI permeation. At the same time, its molecular weight (296.3 Da) and consensus log *P* (−0.38) lie well within the optimal SIZE and LIPO windows, while the number of rotatable bonds (5) and fraction of sp^3^ carbons (0.30) satisfy the FLEX and INSATU criteria. The INSOLU petal remains inside the pink area of the radar, in line with the prediction of very good water solubility (ESOL log *S* = −0.68; “very soluble” by ESOL and “soluble” by Silicos‐IT). Thus, the radar shape indicates that Compound **19** is a highly soluble, moderately flexible molecule with balanced lipophilicity but an elevated polarity that may slightly limit passive oral absorption and can be addressed during further lead optimization.

A more detailed SwissADME readout for **19** supports this interpretation. The molecule shows a moderate bioavailability score (0.55), is classified as having low GI absorption, does not permeate the blood–brain barrier, and is not a P‐glycoprotein substrate. It does not inhibit any of the major CYP isoforms (CYP1A2, 2C19, 2C9, 2D6, and 3A4), which suggests a low risk of metabolic drug–drug interactions, and exhibits poor predicted skin permeation (log Kp = −9.32 cm/s), consistent with its polar, hydrophilic character. Compound **19** fully complies with Lipinski’s rule of five and shows no PAINS or Brenk structural alerts, while only single violations of the Ghose, Veber, Egan, and Muegge filters are observed, all related to its high polarity rather than to problematic substructures. The synthetic accessibility score (2.92) indicates that the scaffold remains synthetically tractable and suitable for further optimization.

Taken together, ADME profiling highlights Compound **19** as a lead that combines excellent aqueous solubility and a clean medicinal chemistry profile with somewhat limited predicted passive oral permeability due to its high TPSA. Within the series, increasing the polarity of the S‐alkyl fragment (amide, carbamate, or ester) improves antioxidant and COX‐2 inhibitory activity but gradually pushes the POLAR vector outside the ideal bioavailability window, as seen most clearly for Compounds **14**, **19**, and **20**. In the SAR part of this section, these structure–property trends are considered in more detail, and potential modifications of the S‐alkyl substituent (e.g., reduction of hydrogen‐bond donors/acceptors or prodrug approaches) are proposed to retain the favorable activity profile of Compound **19** while improving its predicted oral absorption.

**Figure 10:**
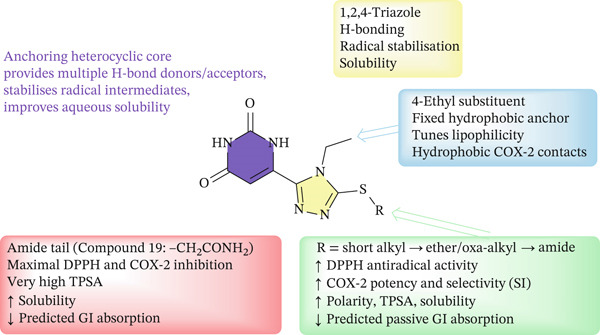
General structure of S‐alkyl 6‐(5‐mercapto‐4‐R^1^‐4H‐1,2,4‐triazol‐3‐yl)pyrimidine‐2,4‐dione derivatives and main fragment–property relationships.

The set of 20 S‐alkyl derivatives of 6‐(5‐mercapto‐4‐ethyl‐4*H*‐1,2,4‐triazol‐3‐yl)pyrimidine‐2,4(1*H*,3*H*)‐dione displays a consistent structure–activity pattern (Figure 10).

Extension of the S‐alkyl substituent and an increase in its electron‐donating character, especially through the introduction of heteroatom‐containing terminal fragments, lead to a gradual enhancement of antioxidant potency in the DPPH assay, with the activity trend **17** < ascorbic acid < **14** < **20** < **13** < **19**. This indicates that the S‐alkyl chain acts as a fine‐tuning element, where additional hydrogen‐bond donors/acceptors and moderate lipophilicity facilitate more efficient radical scavenging by stabilizing the corresponding thiyl and nitrogen‐centered radicals on the triazole–pyrimidinedione core.

A similar tendency is observed for COX‐2 inhibition. Compounds bearing polar amide or related heteroatom‐rich termini on the S‐alkyl linker (notably **13**, **19**, and **20**) show the lowest IC_50_ values and improved COX‐2/COX‐1 selectivity, whereas analogs with short or purely hydrophobic S‐alkyl fragments remain less potent and poorly selective. Among them, Compound **19** combines the most favorable balance of parameters, exhibiting the lowest IC_50_(COX‐2) and the highest SI (SI ≈ 8.2), which suggests that an optimal combination of hydrogen bonding centers and chain length in the S‐substituent is critical for productive interactions within the COX‐2 active site.

From the physicochemical point of view, the most active molecules share a common profile: molecular weights in the range 280–320 Da, consensus log *P* values close to 0–2, and high polar surface areas (≈150–165 Å^2^). According to SwissADME, this combination leads to excellent aqueous solubility and multiple hydrogen bonding opportunities, which is advantageous both for DPPH radical trapping and for target engagement with COX‐2 but is accompanied by a predicted decrease in passive GI absorption. Thus, within this chemotype, increasing the polarity and donor/acceptor density of the S‐alkyl fragment improves antioxidant and COX‐2 inhibitory activity at the expense of oral drug‐like permeability, making Compound **19** a polar, highly active lead suitable for further optimization (e.g., through prodrug or bioisosteric strategies) to balance efficacy and pharmacokinetic properties.

#### 3.5.1. Future Development Strategies

The present series provides a modular platform for further optimization toward orally bioavailable, COX‐2 selective anti‐inflammatory agents with antioxidant benefit. First, polarity can be fine‐tuned by replacing the terminal carboxamide of Compound **19** with less polar isosteres (e.g., nitrile, heteroaryl, or carbamate) or by exploring prodrug approaches, while maintaining the key H‐bonding pattern suggested by docking. Second, systematic expansion of the S‐alkyl space (branched alkyl, benzyl/heteroaryl methyl, and substituted acetamides) will enable clearer SAR trends and may improve selectivity by better occupancy of the COX‐2 secondary pocket. Third, the most active members should be prioritized for extended profiling: COX‐1/COX‐2 testing across the full set, celecoxib benchmarking, microsomal stability and plasma stability, and early in vivo anti‐inflammatory models. Finally, integrating experimental antioxidant assays with DFT descriptors (BDE, IP, PA, and ETE) across solvents will support mechanism‐guided optimization of radical scavenging performance.

## 4. Conclusion

Taken together, the results demonstrate that the 20 new S‐alkyl derivatives of 6‐(5‐mercapto‐4‐ethyl‐4*H*‐1,2,4‐triazol‐3‐yl)pyrimidine‐2,4(1*H*,3*H*)‐dione exhibit clear structure–property relationships: elongation and the electron‐donating nature of the S‐alkyl substituent, as well as an optimal arrangement of donor sites within the heterocyclic fragment, correlate with an increase in radical scavenging activity in the order **17** < ascorbic acid < **14** < **20** < **13** < **19** (DPPH assay, up to 72.34% inhibition for Compound **19**). In vitro, Compound **19** combines the lowest IC_50_(COX − 2) = 24.5 ± 0.9 *μ*M with a high SI (SI ≈ 8.2), whereas Compounds **13** and **20** display a moderately COX‐2 selective profile (SI ≈ 1.3–1.2), and Compounds **14** and **17** behave as COX‐1‐biased analogs, resembling classical NSAIDs. Quantum chemical calculations support the predominance of the SPLET mechanism for most systems and highlight **19** (as well as **15** and **20**) as compounds with the lowest *Δ* *G* for the first step, in agreement with their experimental antioxidant activity, while molecular docking further predicts high affinity of **14**, **19**, and **20** for COX‐2 and a set of related targets (1EQG, 3LN1, and 5F19) driven by a stable network of hydrogen bonding and hydrophobic contacts. The combined DPPH, COX, docking, and quantum chemical data thus identify Compound **19** as the primary lead structure, combining potent antioxidant properties with pronounced, selective COX‐2 inhibition, whereas Compounds **13** and **20** can be considered secondary leads with moderate COX‐2 selectivity and good radical scavenging potential, suitable for further structure‐guided optimization.

## Funding

This work was supported by the Grant of the President of Ukraine for the support of scientific research and development by young scientists (Grant No. 2025.05/0022), project “Hybrid 1,2,4‐triazole and orotic acid compounds with radioprotective, wound‐healing, and regenerative properties for the needs of the civilian population and the Armed Forces of Ukraine.”

## Disclosure

Parts of this study were previously presented as a conference abstract at the 29th International Electronic Conference on Synthetic Organic Chemistry (ECSOC‐29), MDPI Proceedings (https://www.mdpi.com/2673-4583/18/1/46). The present manuscript substantially expands the abstract with full experimental details, ADME profiling, antioxidant evaluation, and molecular docking analysis.

## Conflicts of Interest

The authors declare no conflicts of interest.

## Supporting information


**Supporting Information** Additional supporting information can be found online in the Supporting Information section. The complete ^1^H and ^13^C NMR spectra, LC‐MS data, and elemental analysis data for all synthesized compounds.

## Data Availability

The data that support the findings of this study are available from the corresponding author upon reasonable request.
