# Supplementary material for: Novel 1,2,4‐Triazole–Thiopyrimidine Hybrids as COX‐2 Inhibitors: Synthesis, ADME Profiling, Antioxidant Activity, and Molecular Docking
Source: Biomed Res Int. 2026 Mar 31;2026:4477854. doi: 10.1155/bmri/4477854 (PMC13140806; doi:10.1155/bmri/4477854)

**Supplementary information**

**Novel 1,2,4-Triazole–Thiopyrimidine Hybrids as COX-2 Inhibitors: Synthesis, ADME Profiling, Antioxidant Activity and Molecular Docking**

Yuriy Karpenko^1^, Volodymyr Parchenko^1^, Olexandr Panasenko^1^, Oleksii Bihdan^2^, Iryna Pukhalska^3^, Oleg Nikiforov^4^, Nataliia Nahorna^5^, Volodymyr Nahornyi^3^, Olena Roik^6^

^1^Department of Toxicological and Inorganic Chemistry, Faculty of Pharmacy, Zaporizhzhia State Medical and Pharmaceutical University, Zaporizhzhia, Ukraine

^2^Department of Clinical Pharmacy, Pharmacotherapy, Pharmacognosy and Pharmaceutical Chemistry, Faculty of Pharmacy, Zaporizhzhia State Medical and Pharmaceutical University, Zaporizhzhia, Ukraine

^3^Department of Drug Technology, Faculty of Pharmacy, Zaporizhzhia State Medical and Pharmaceutical University, Zaporizhzhia, Ukraine

^4^Department of Obstetrics, Gynecology and Reproductive Medicine, Faculty of Medicine, Zaporizhzhia State Medical and Pharmaceutical University, Zaporizhzhia, Ukraine

^5^Department of Physicocolloid and Analytical Chemistry, Faculty of Pharmacy, Zaporizhzhia State Medical and Pharmaceutical University, Zaporizhzhia, Ukraine

^6^Department of Industrial Pharmacy, Kyiv National University of Technology and Design, Kyiv, Ukraine

✉**Corresponding author**

***As. prof., PhD Yuriy KARPENKO**

**Email: karpenko.y.v@gmail.com**

**Tel:** +38 063-973-44-27

https://orcid.org/0000-0002-4390-9949

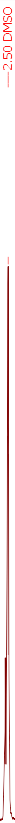


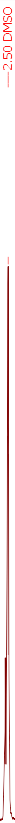

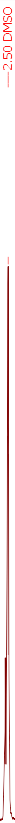

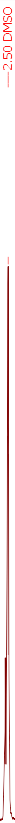


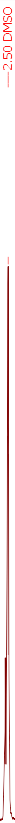


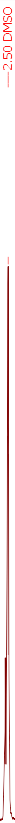


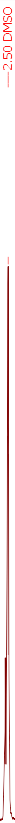


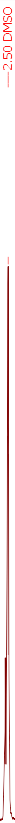


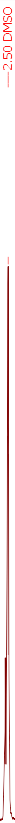


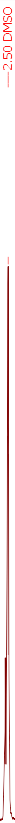


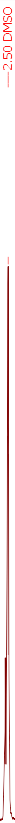

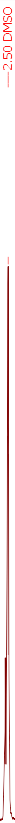


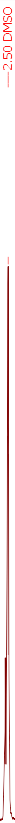


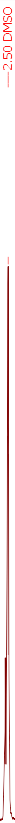


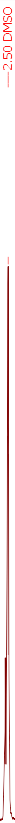


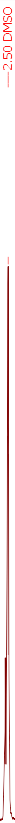

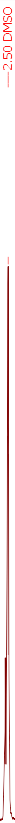


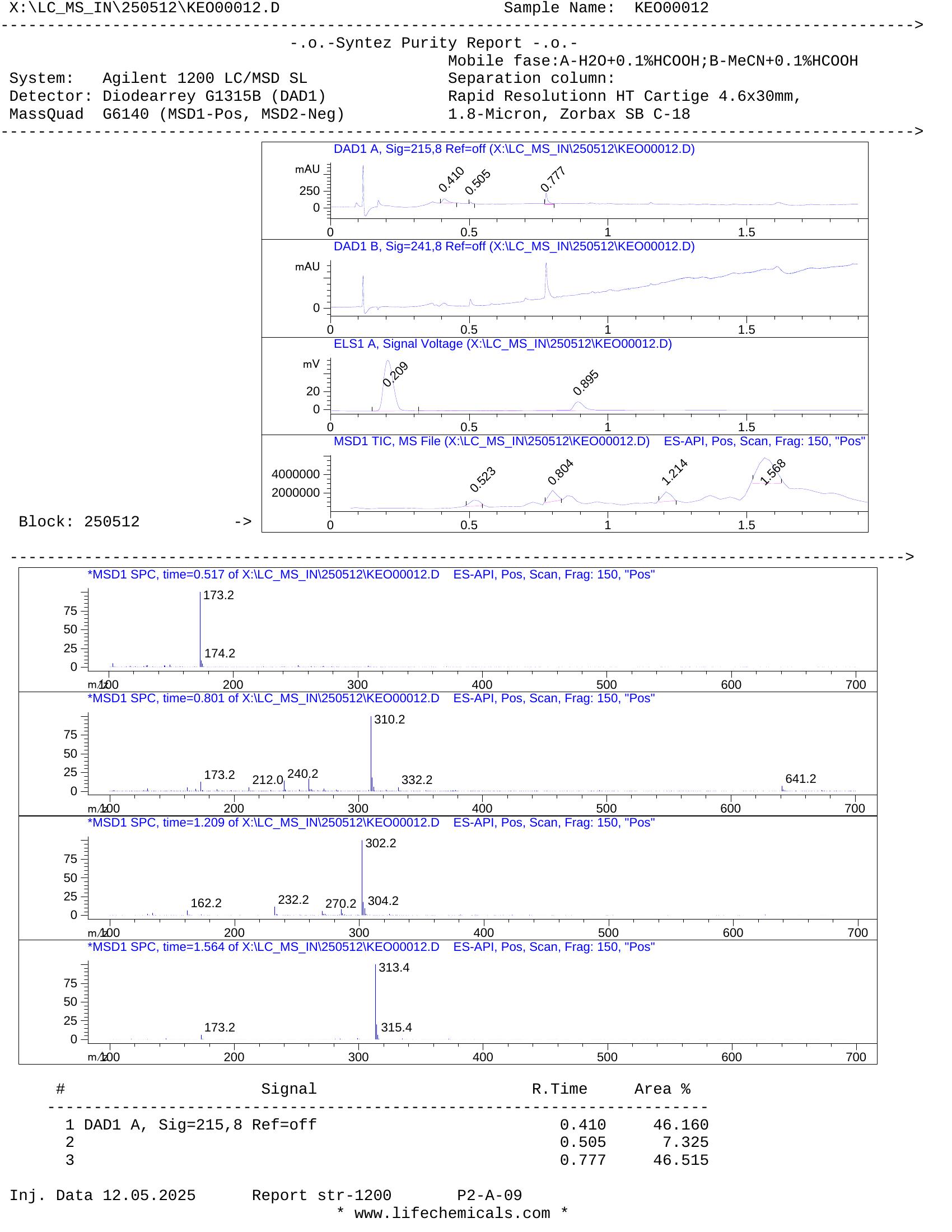


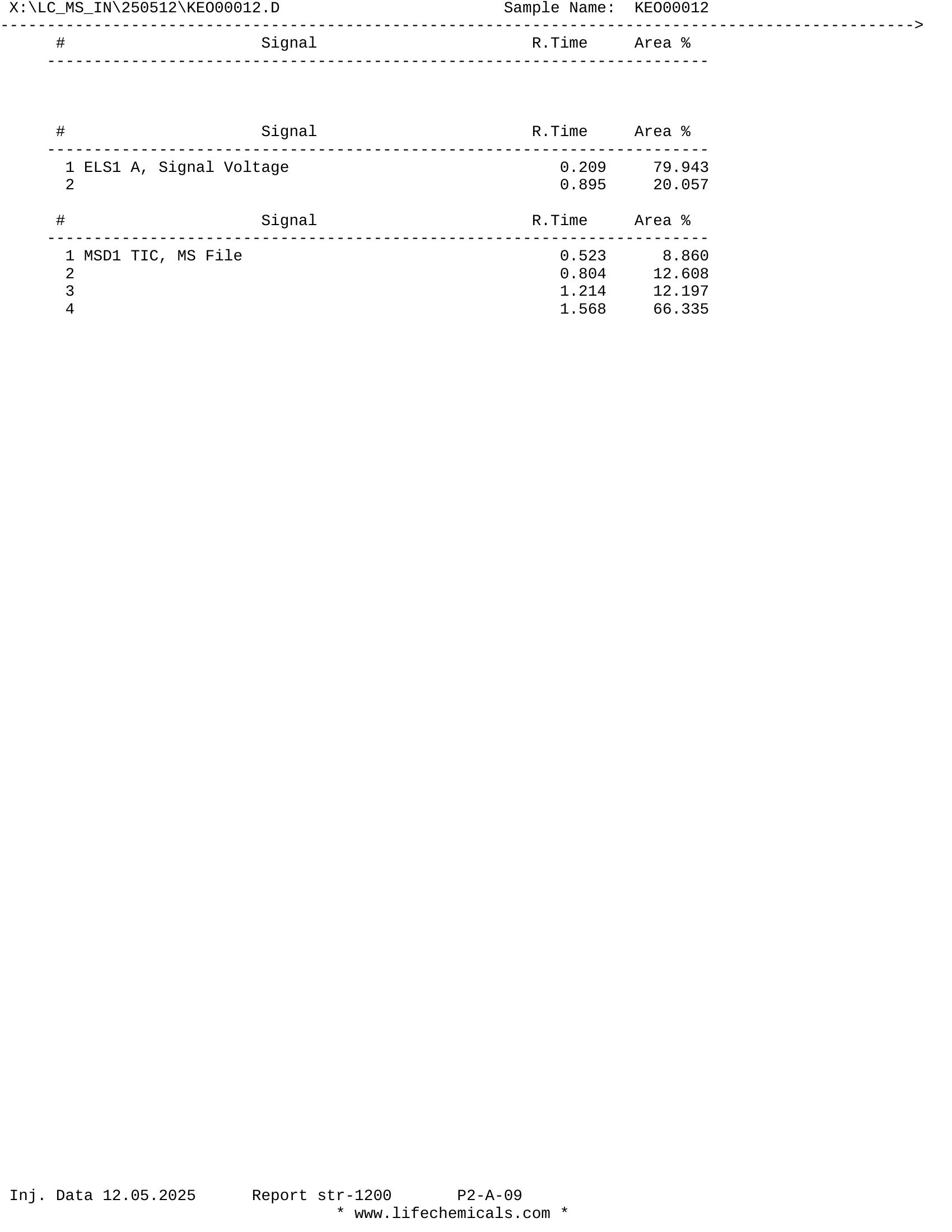


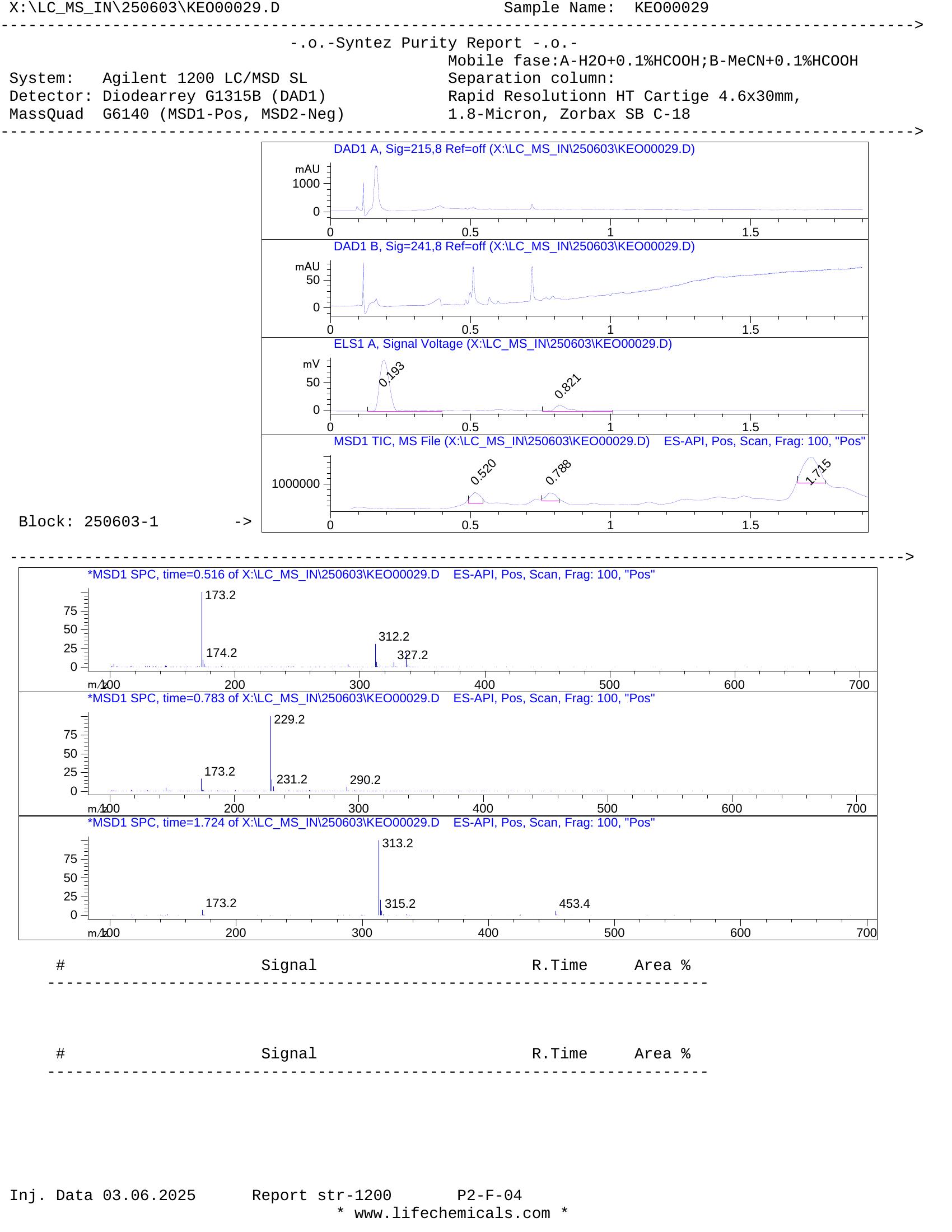


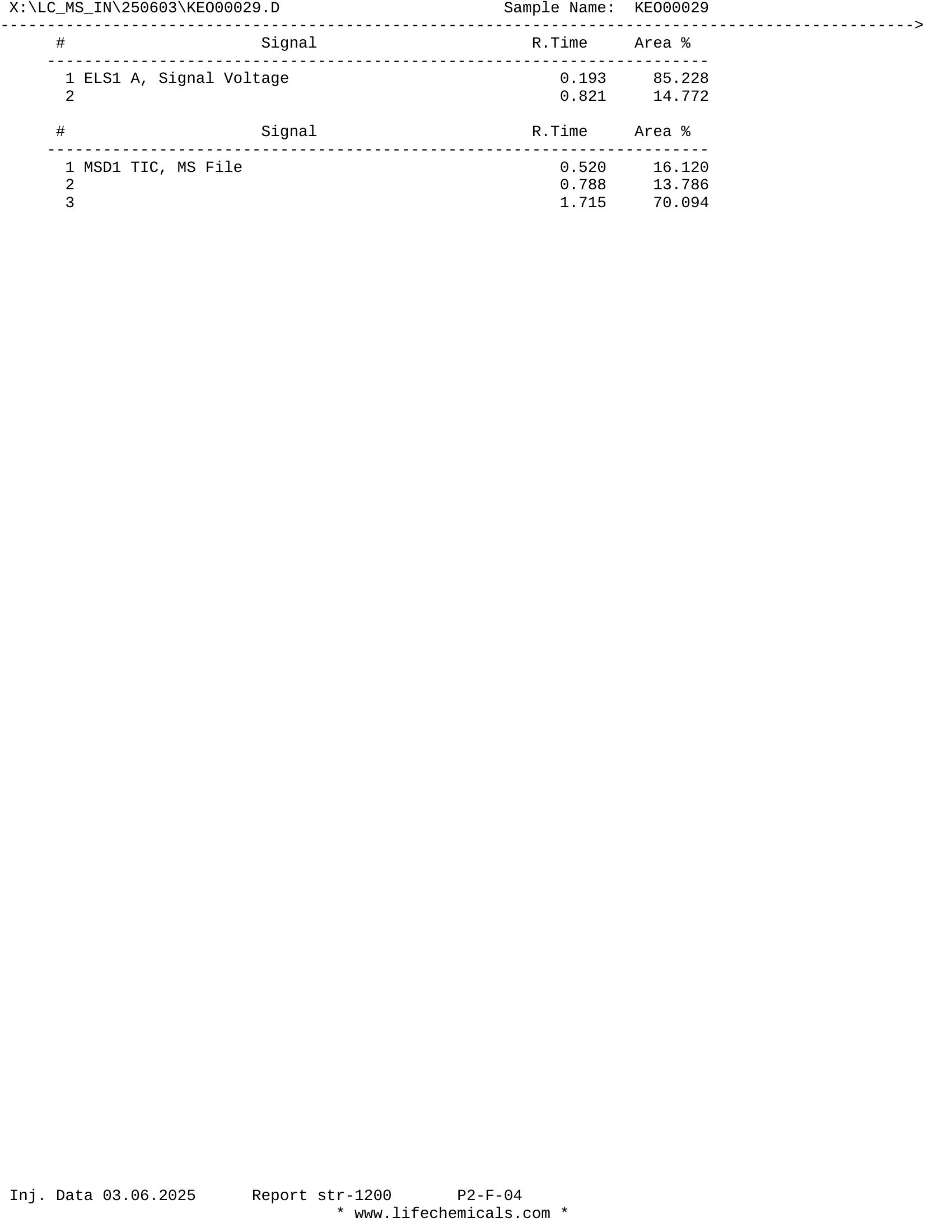


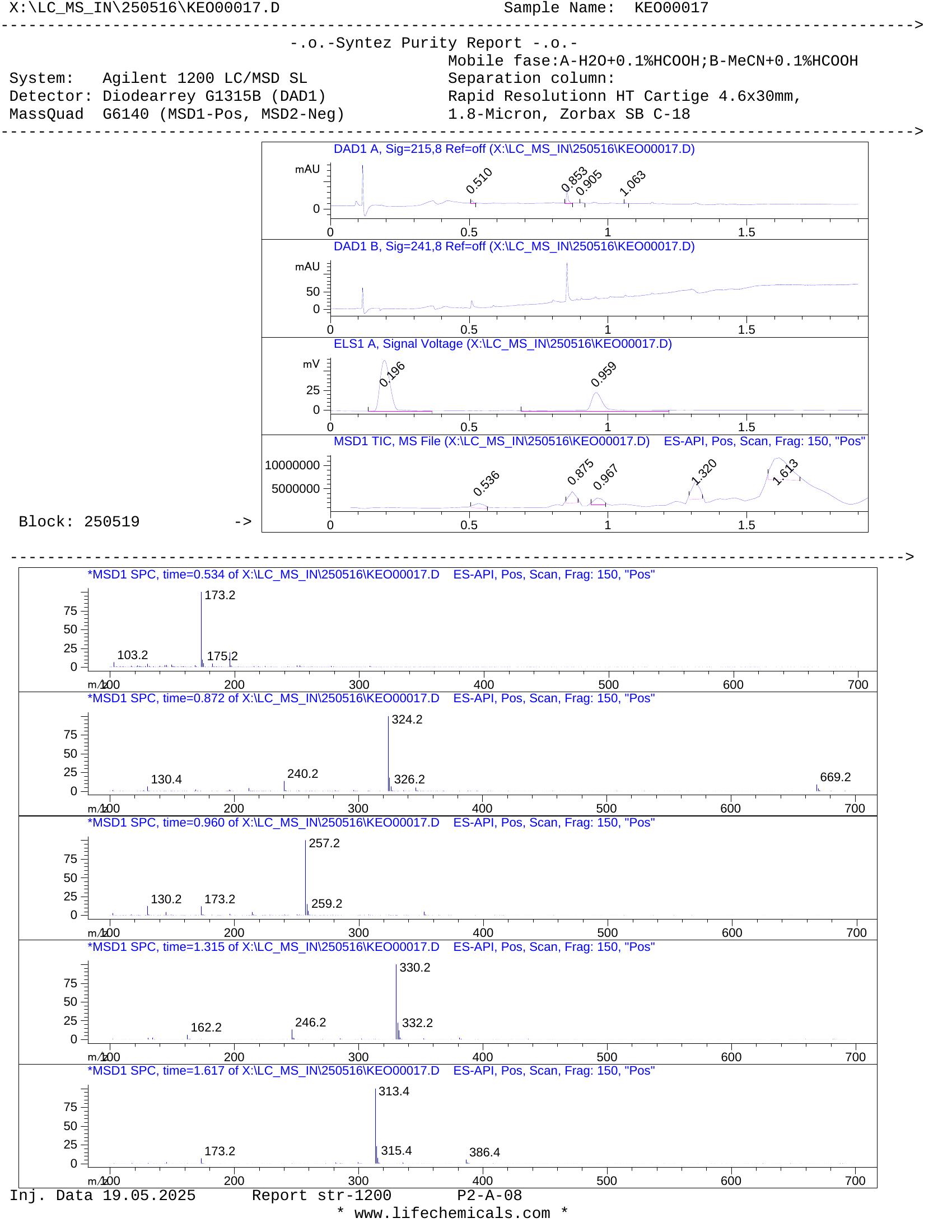


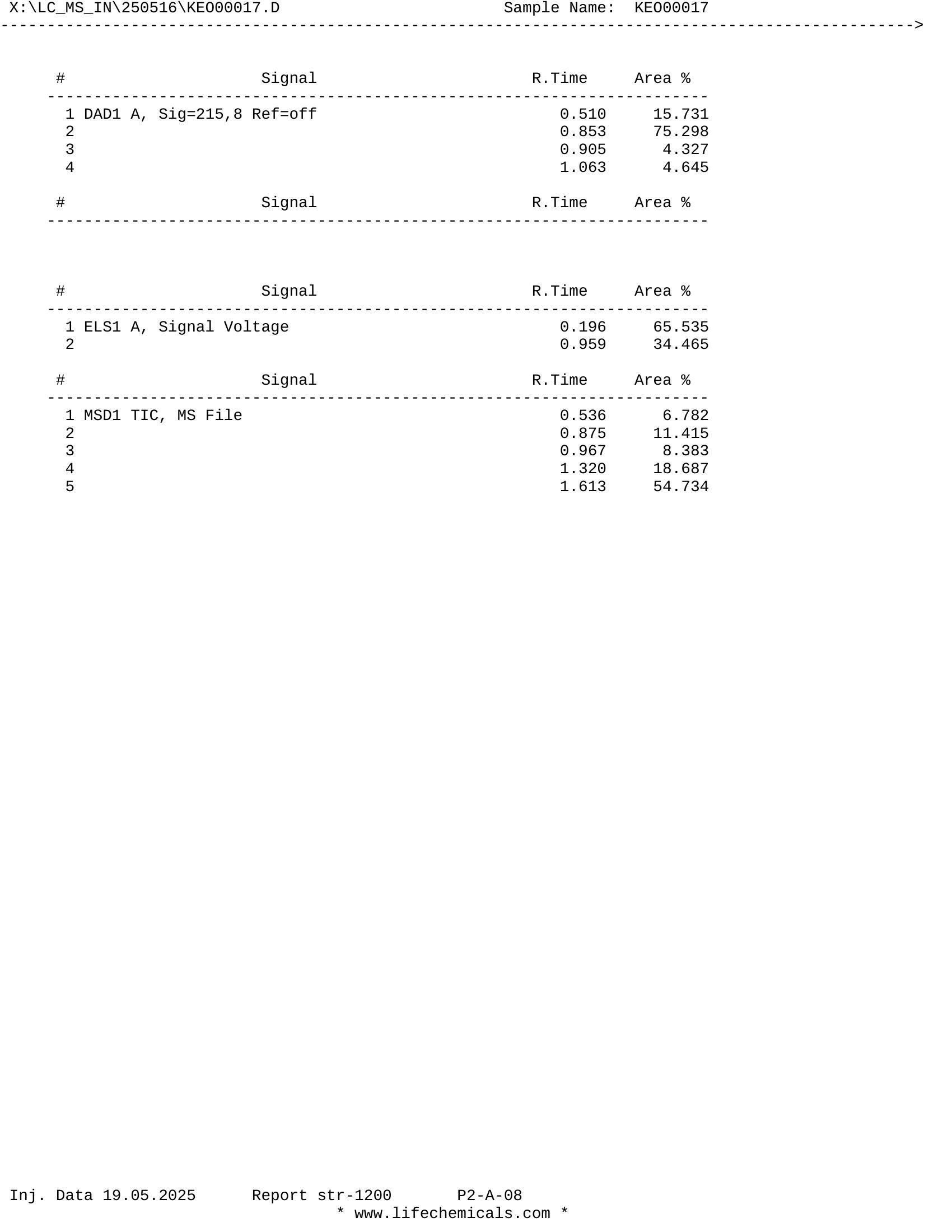


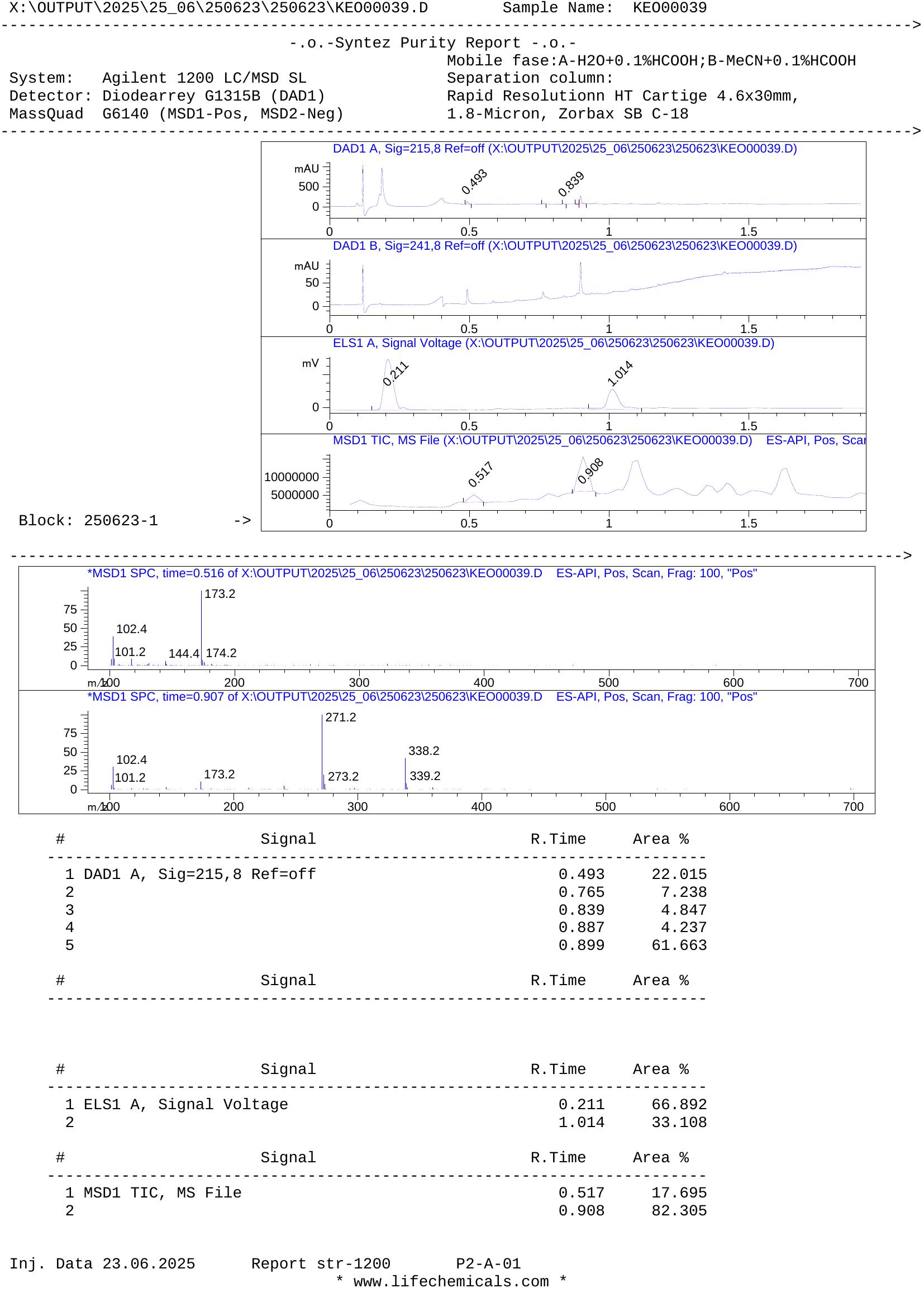


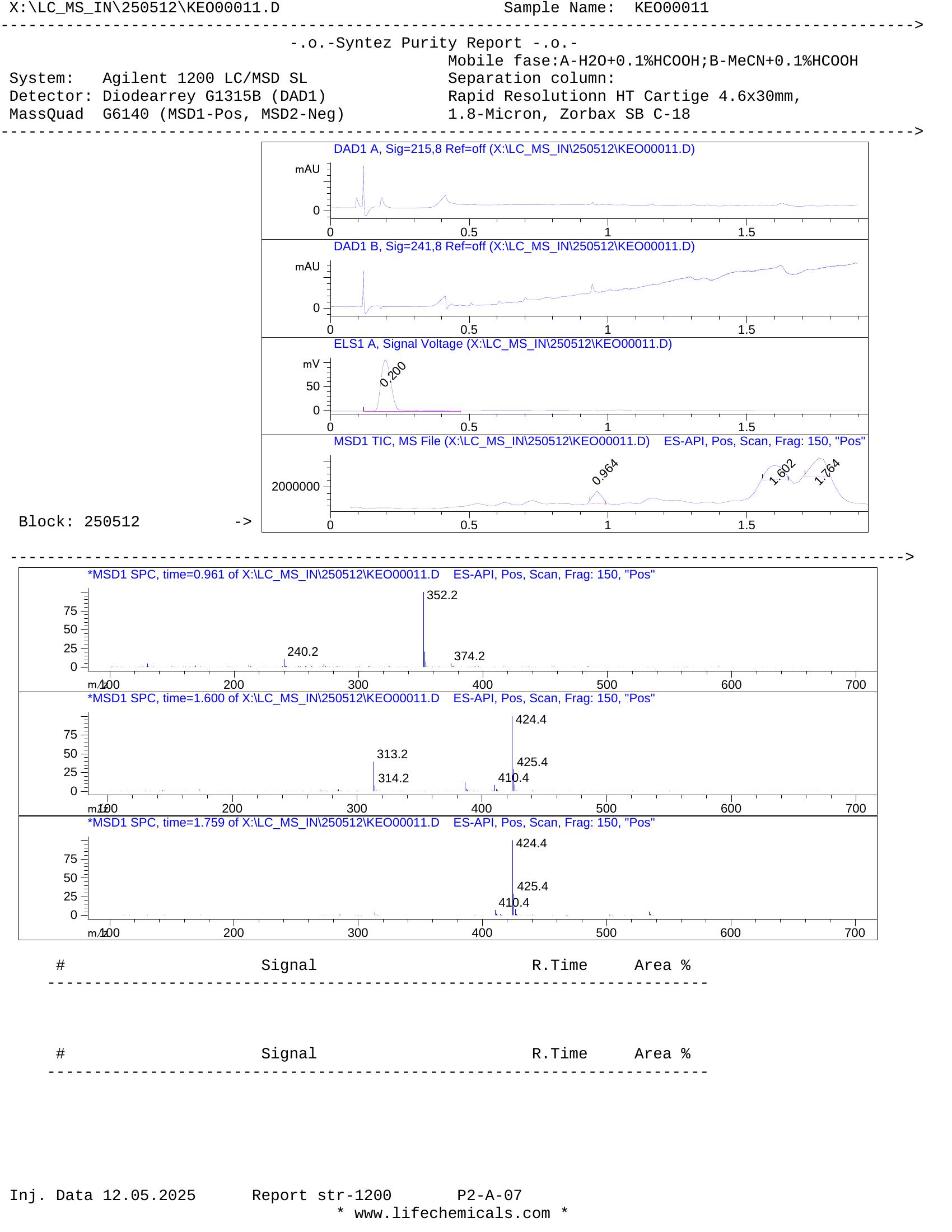


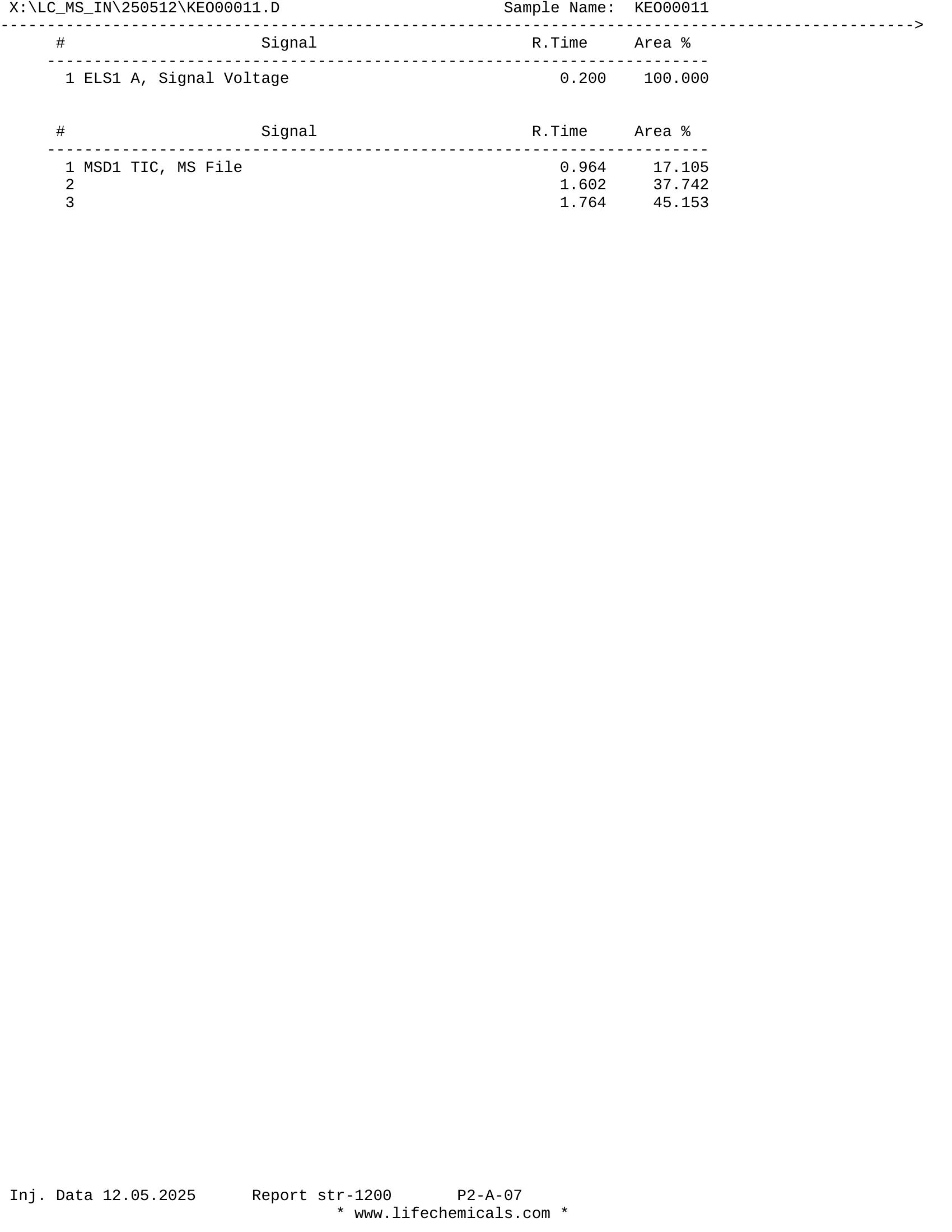

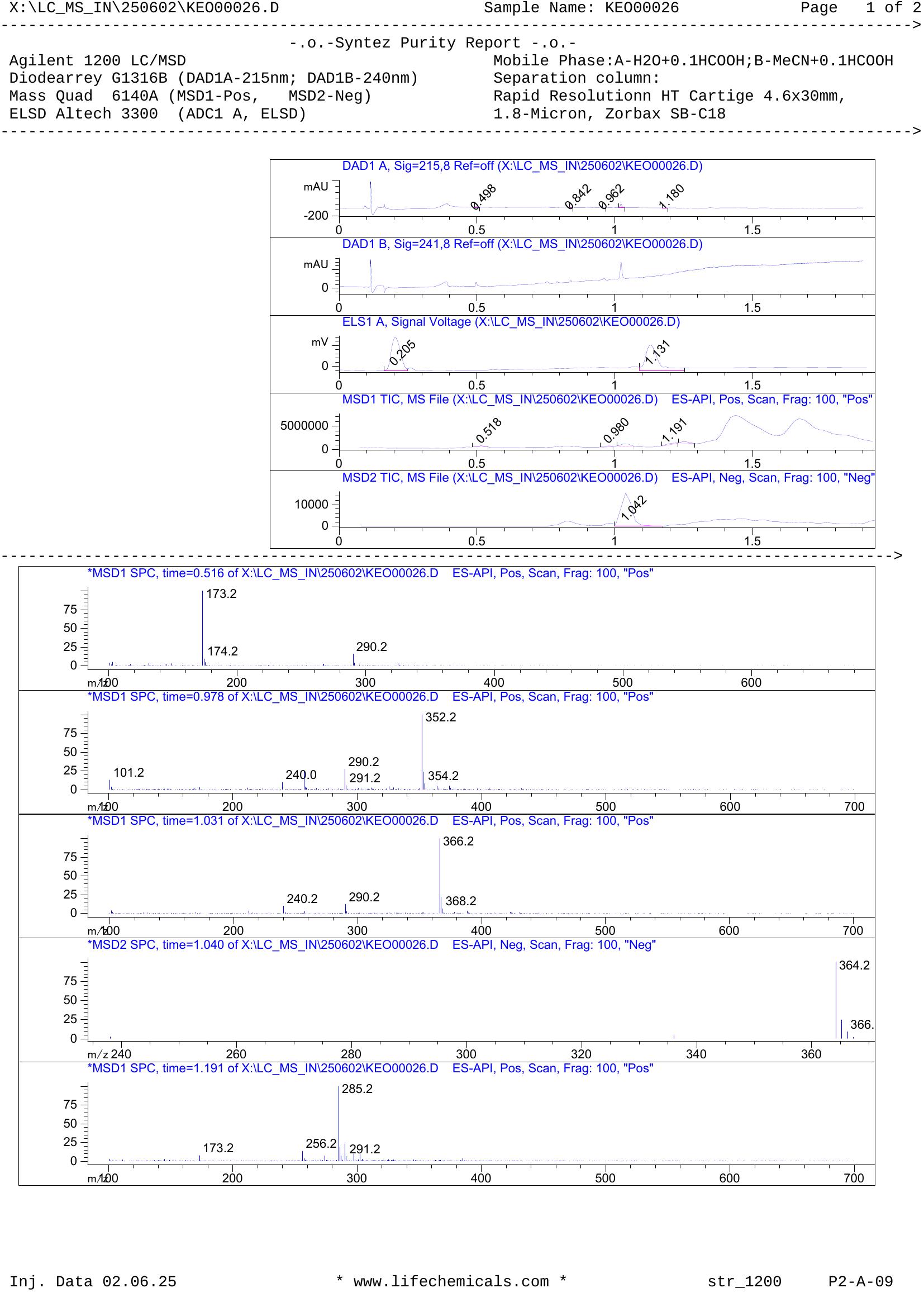


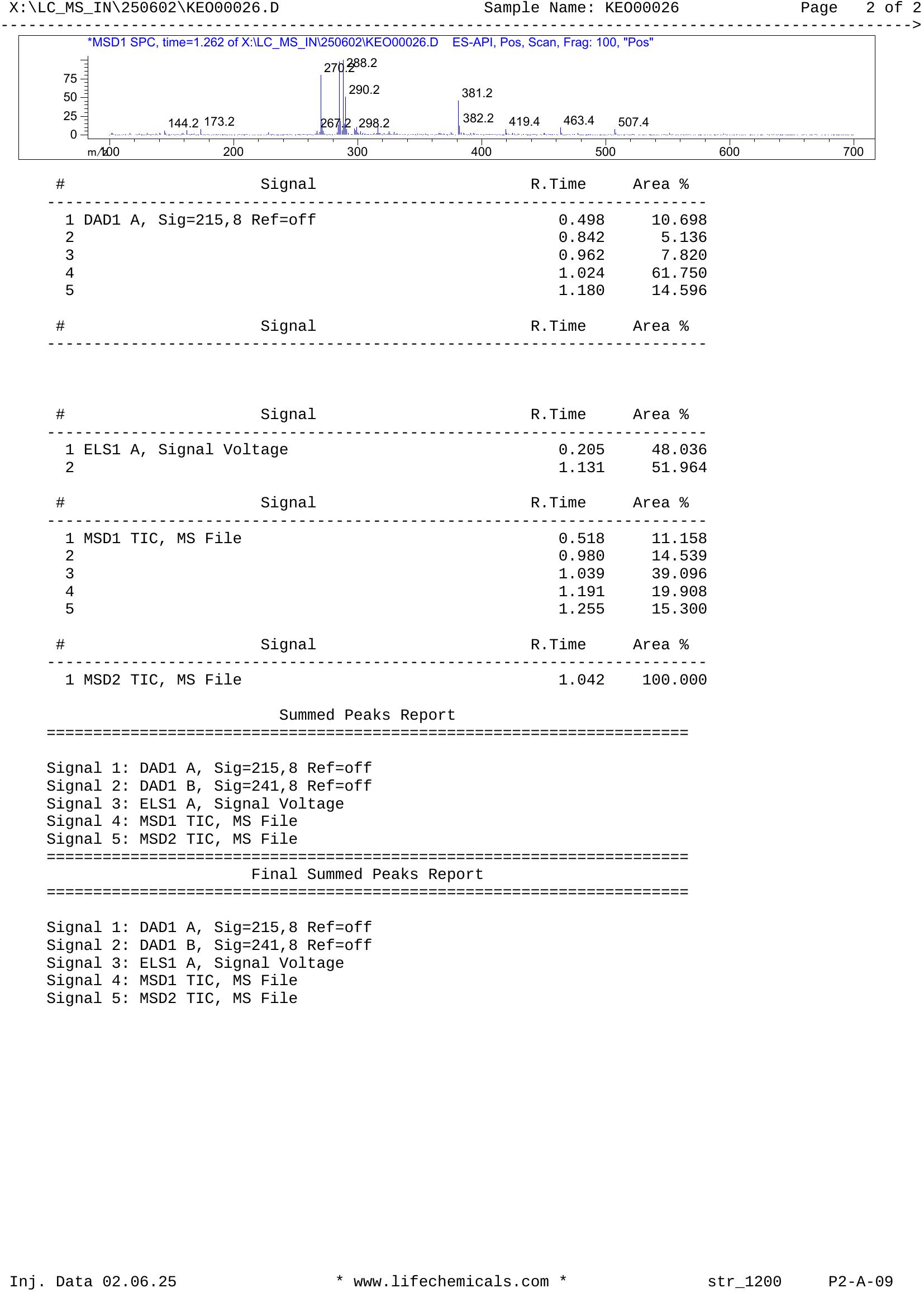


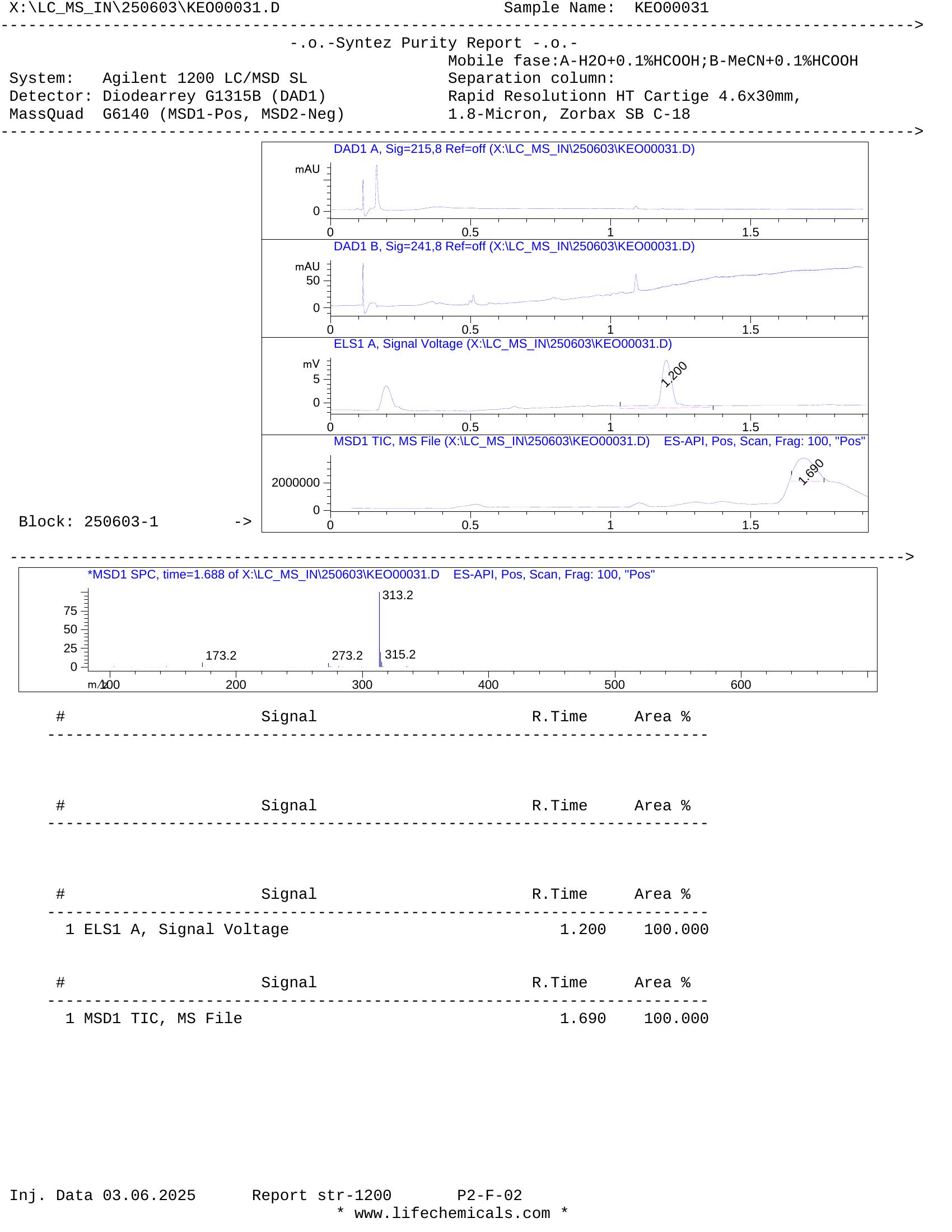


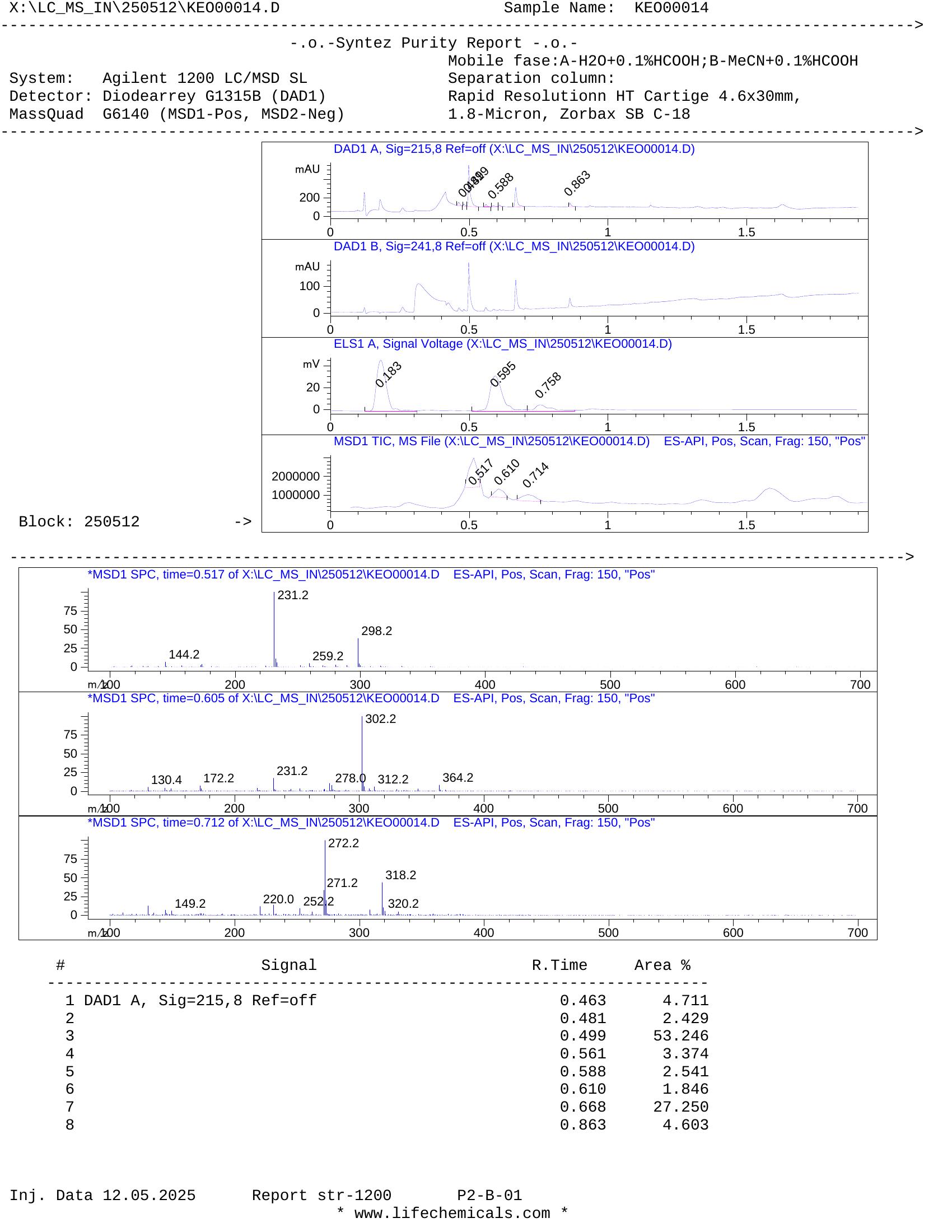


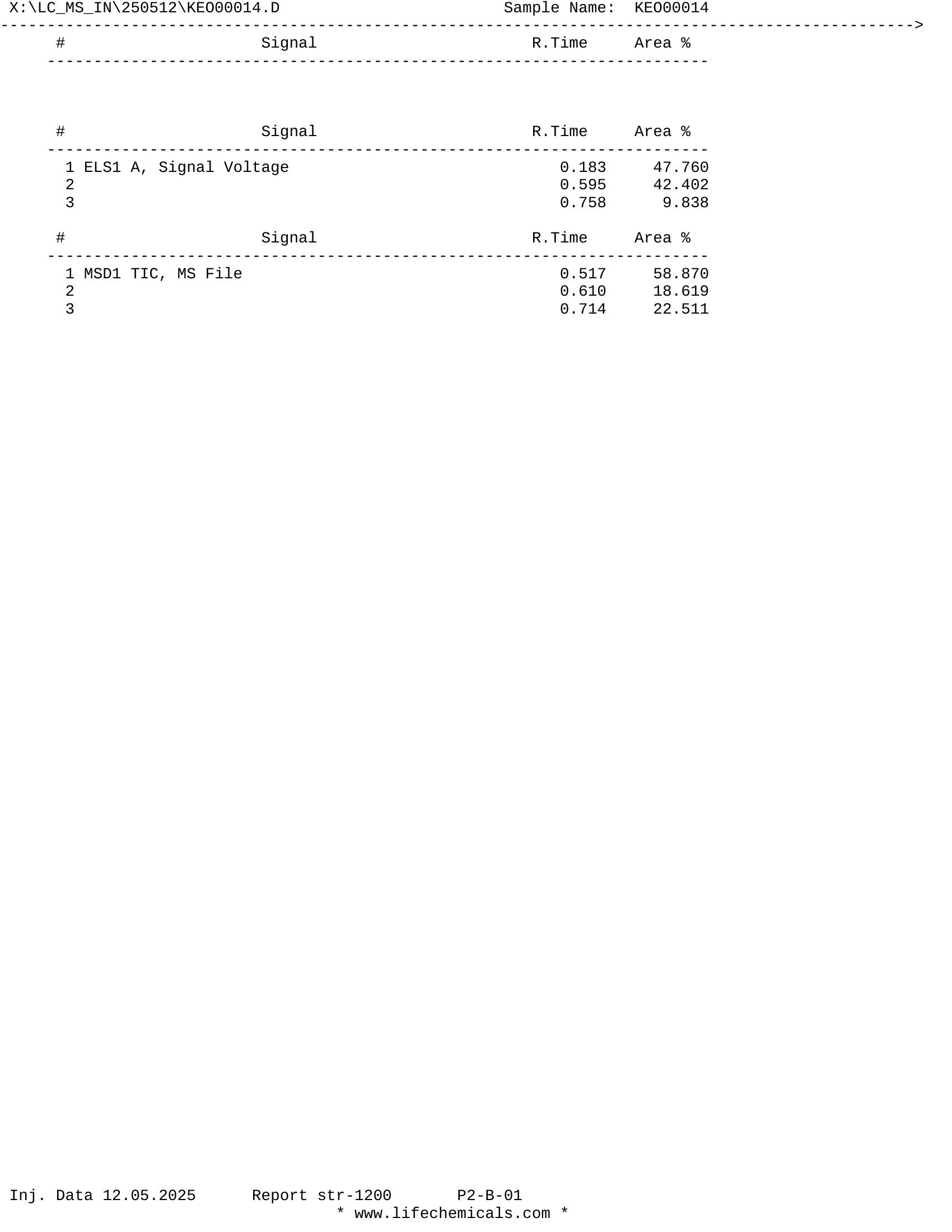


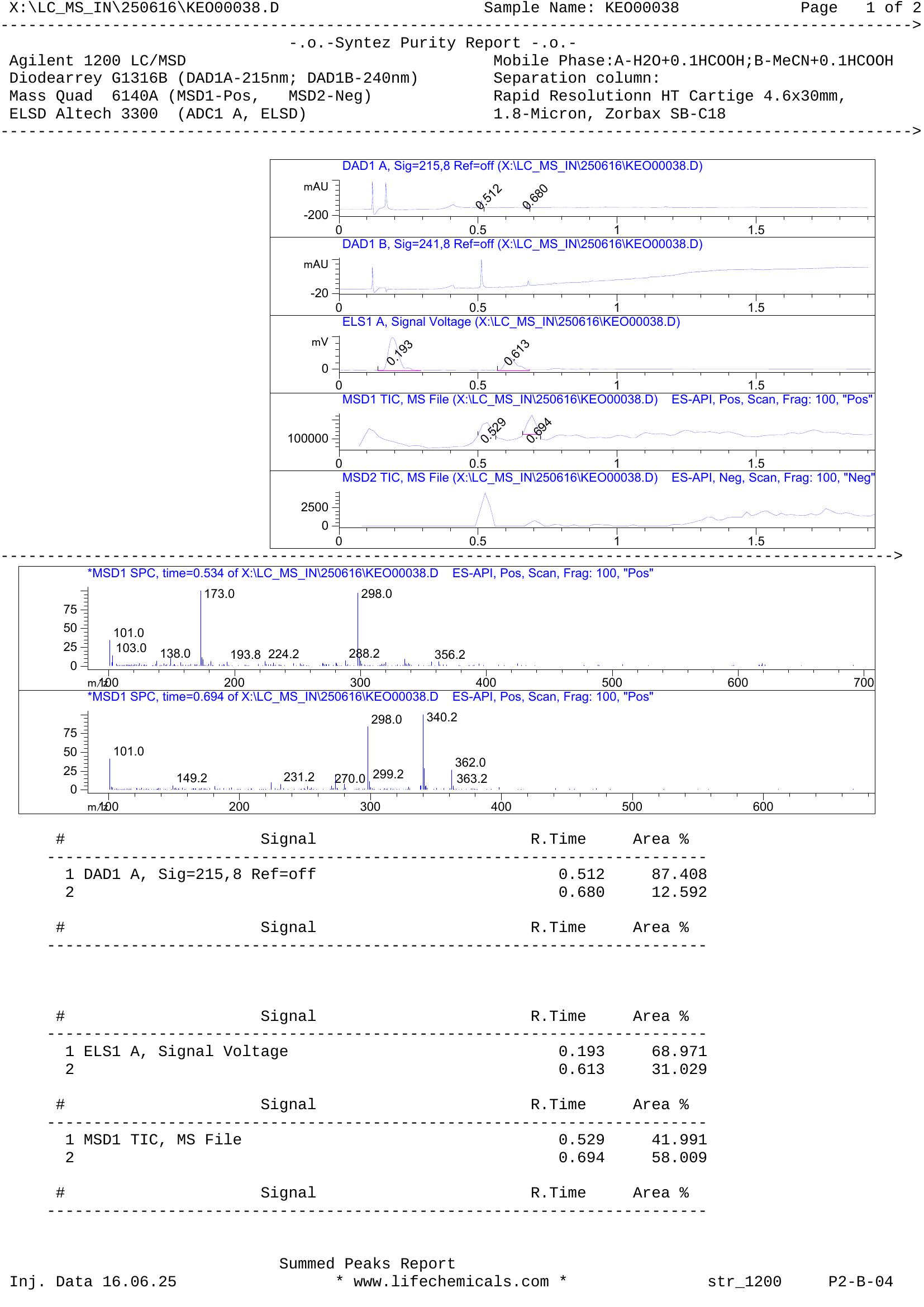


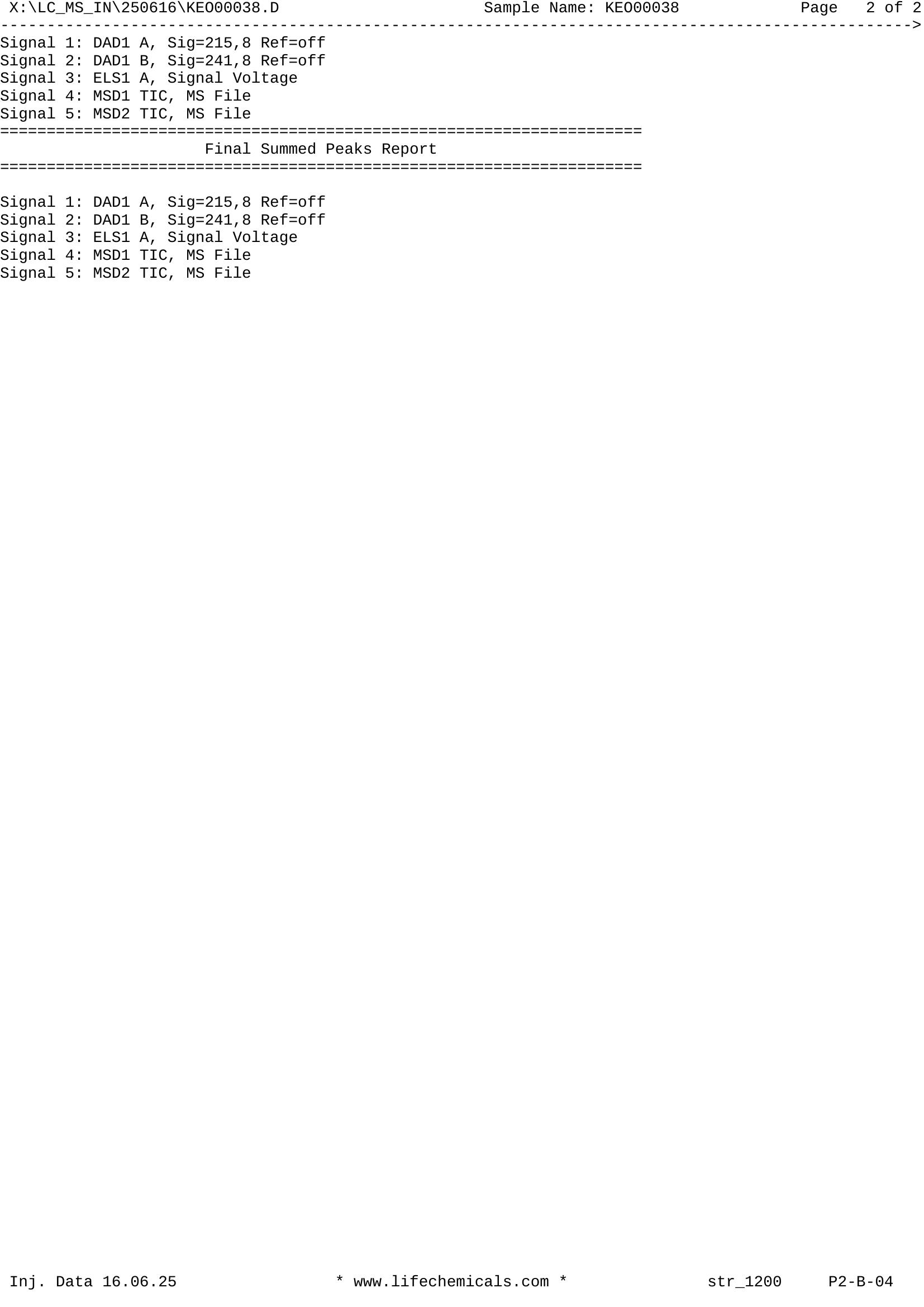


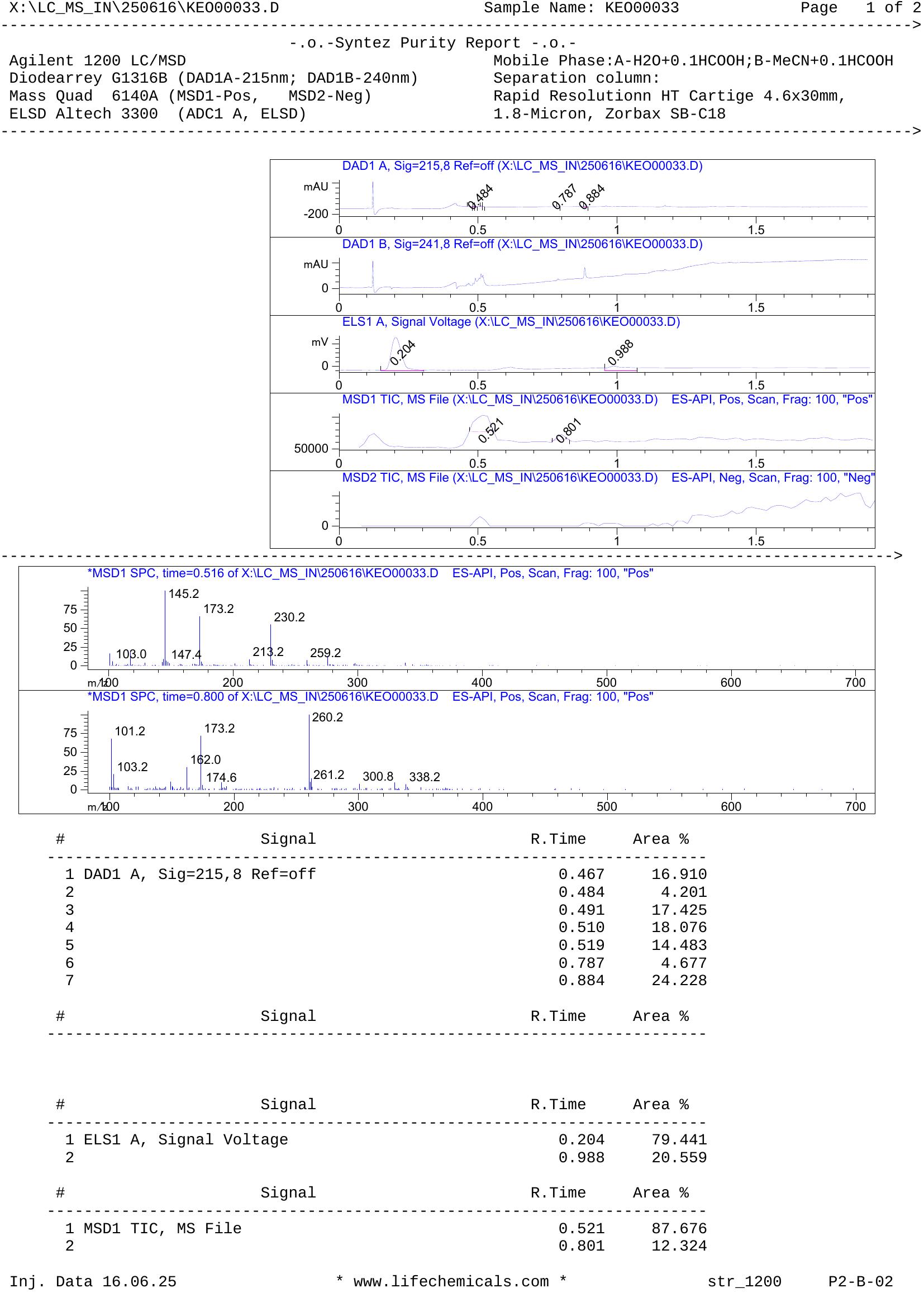


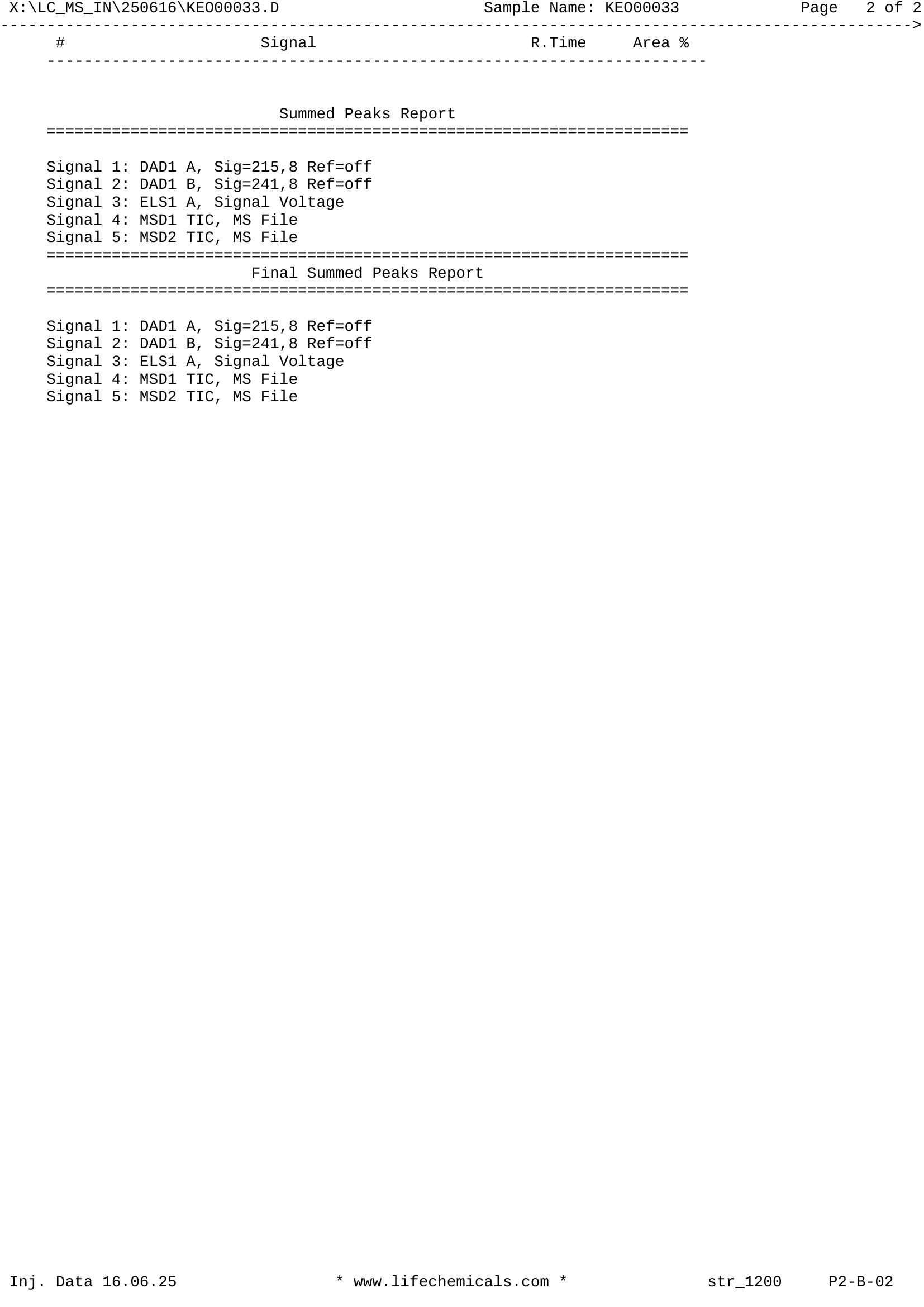


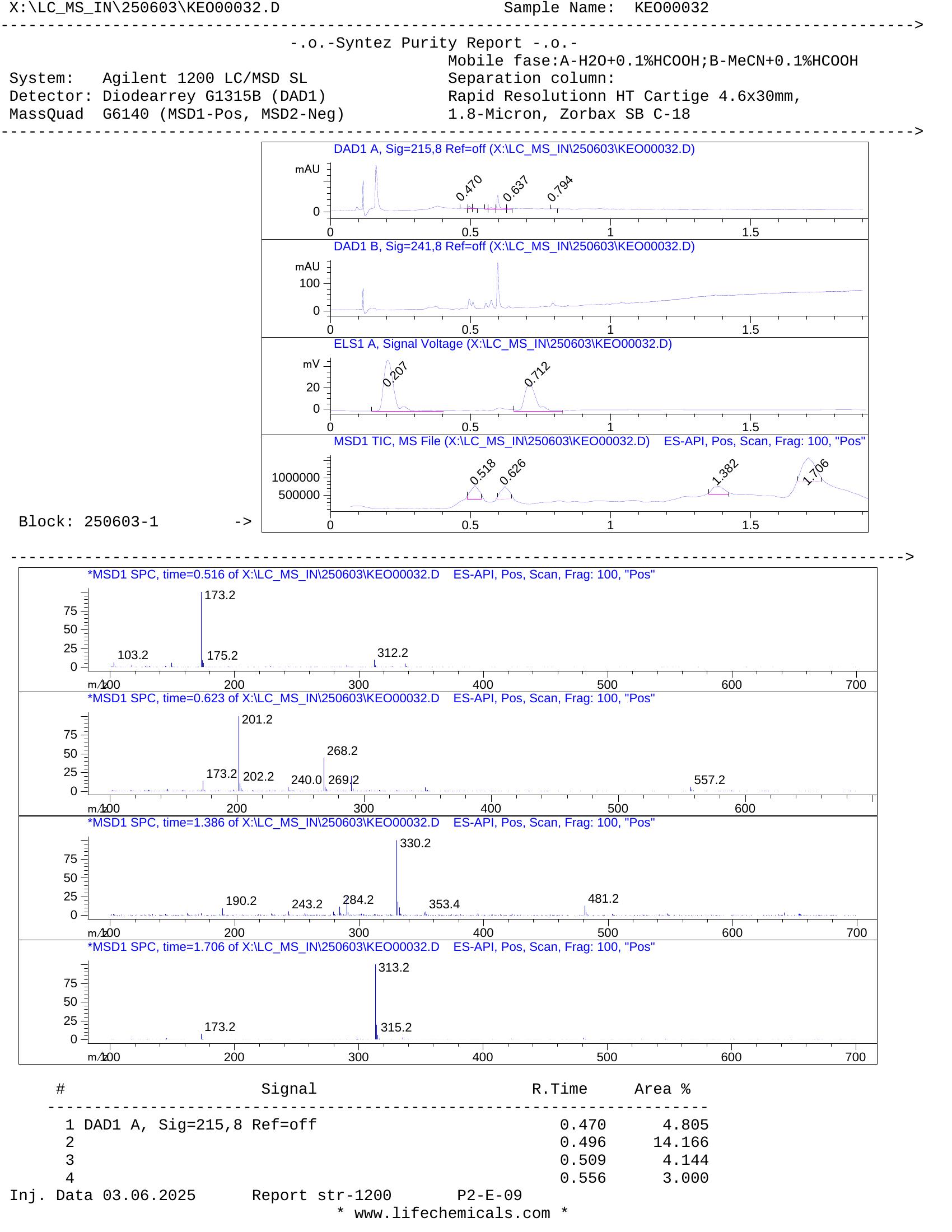


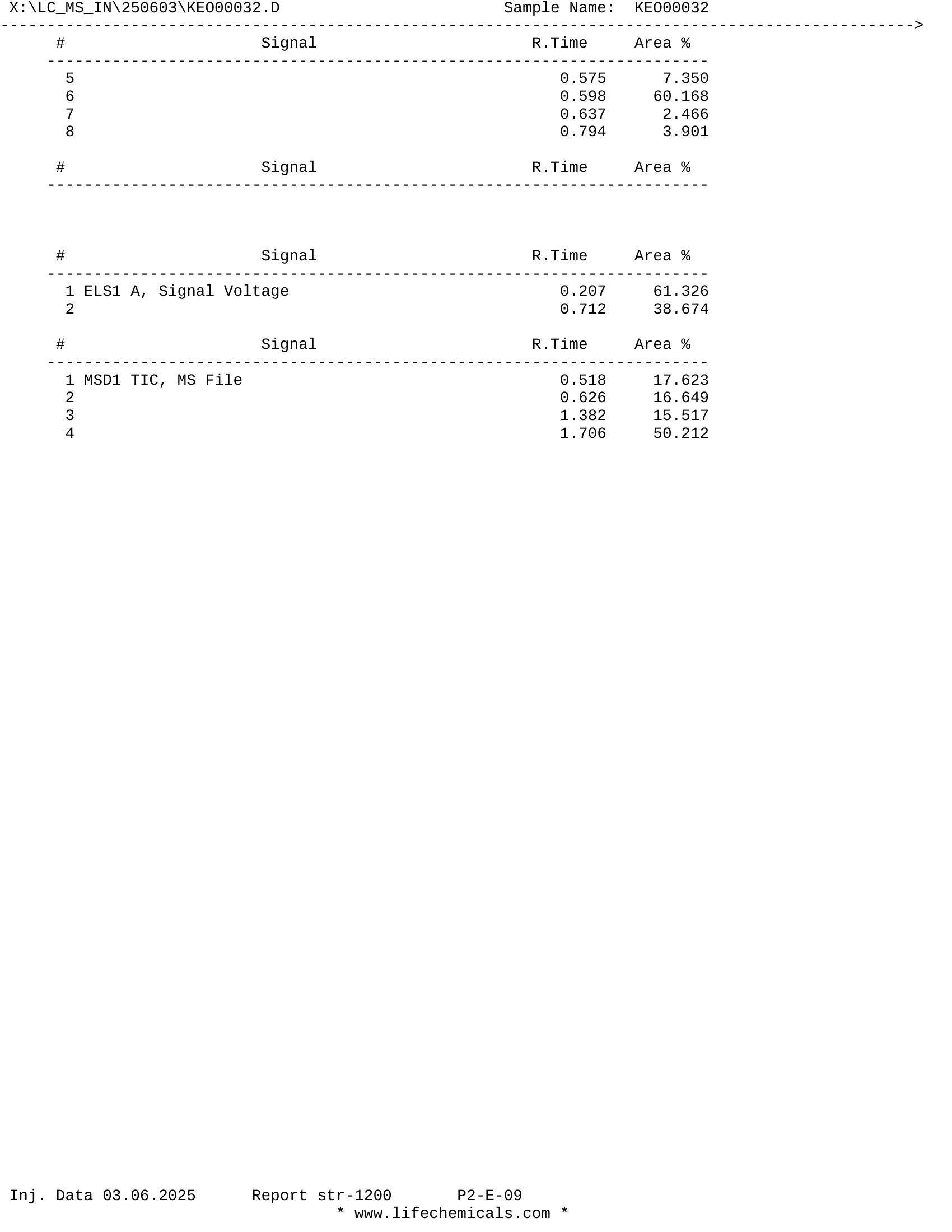


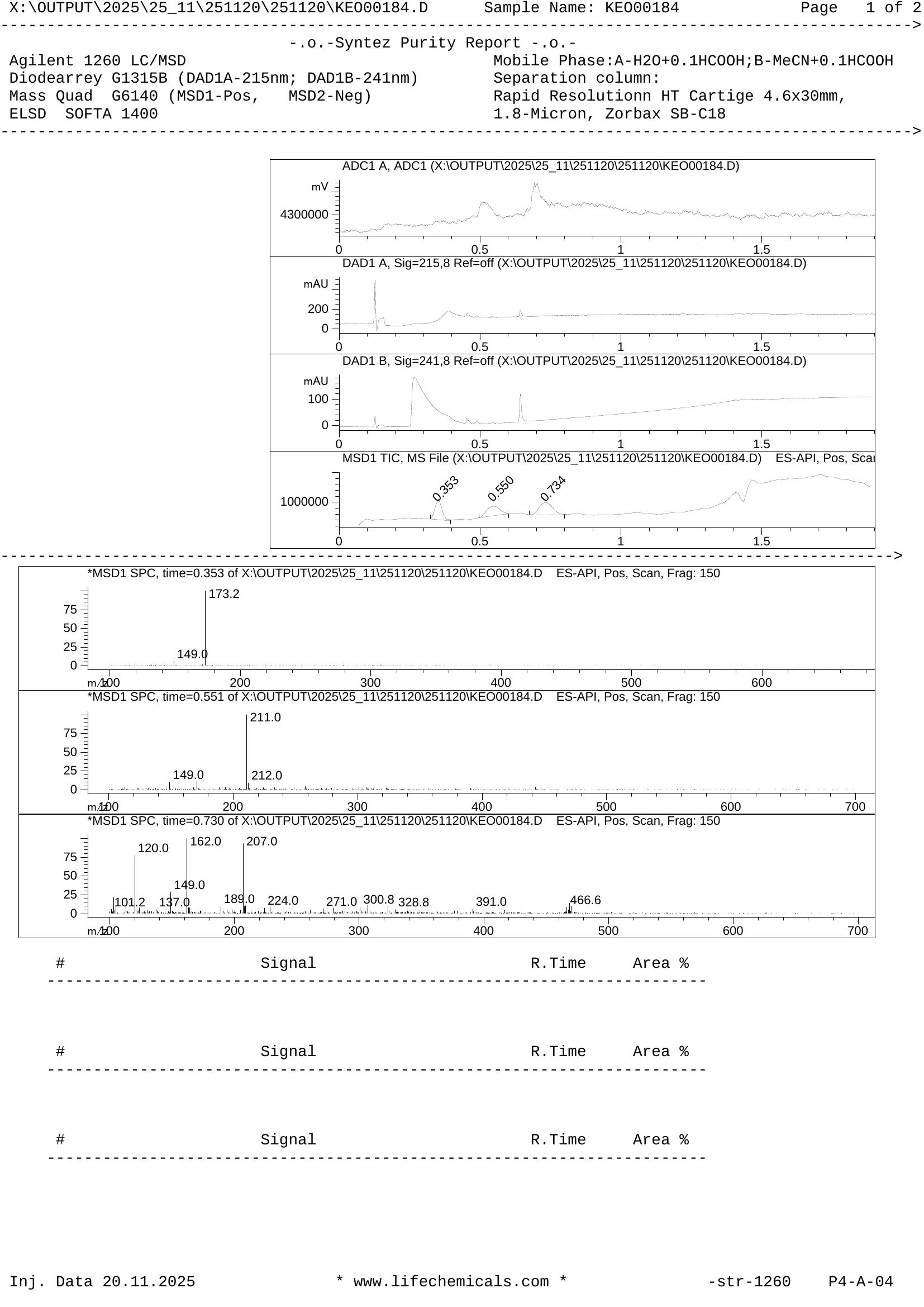


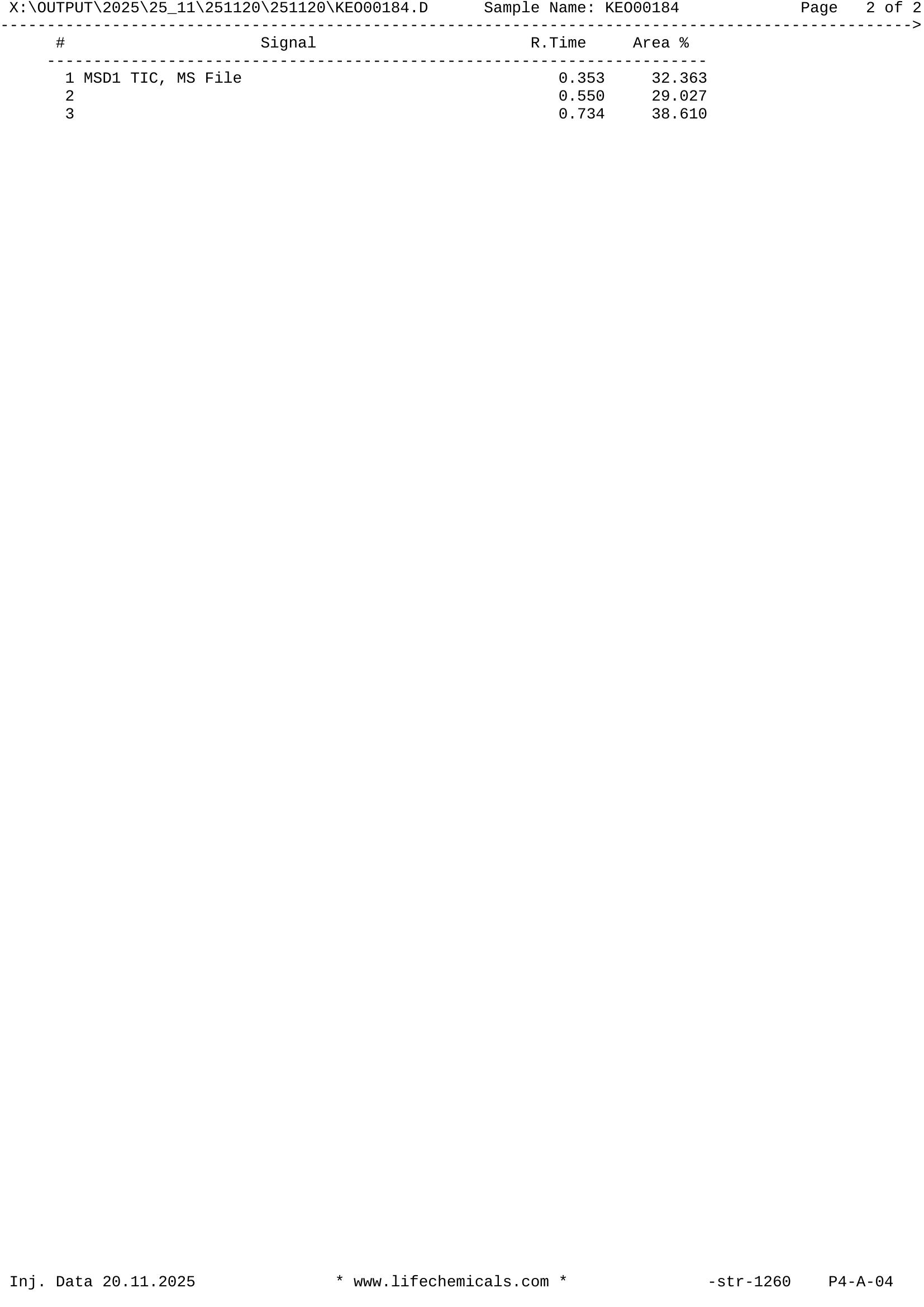


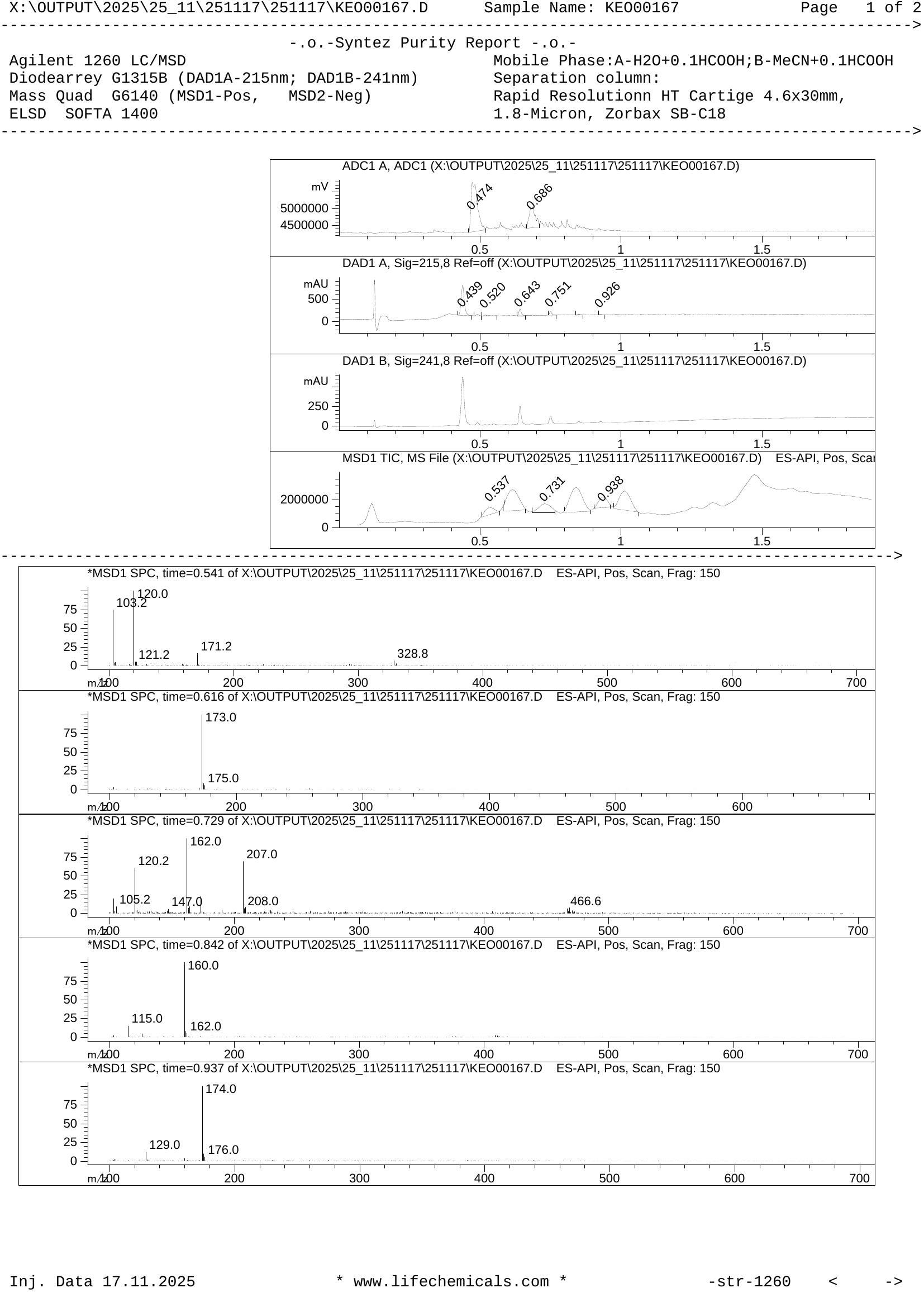


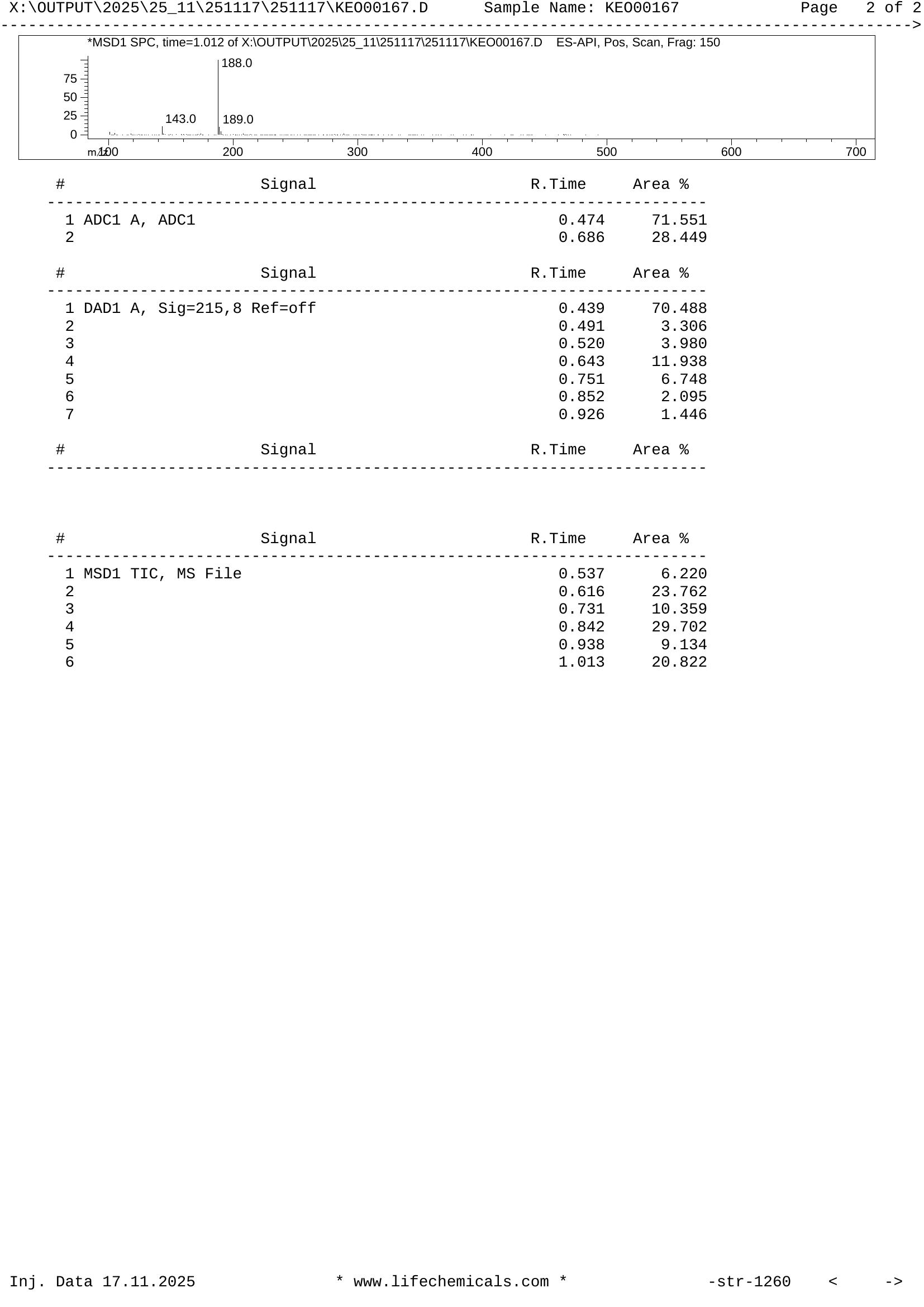


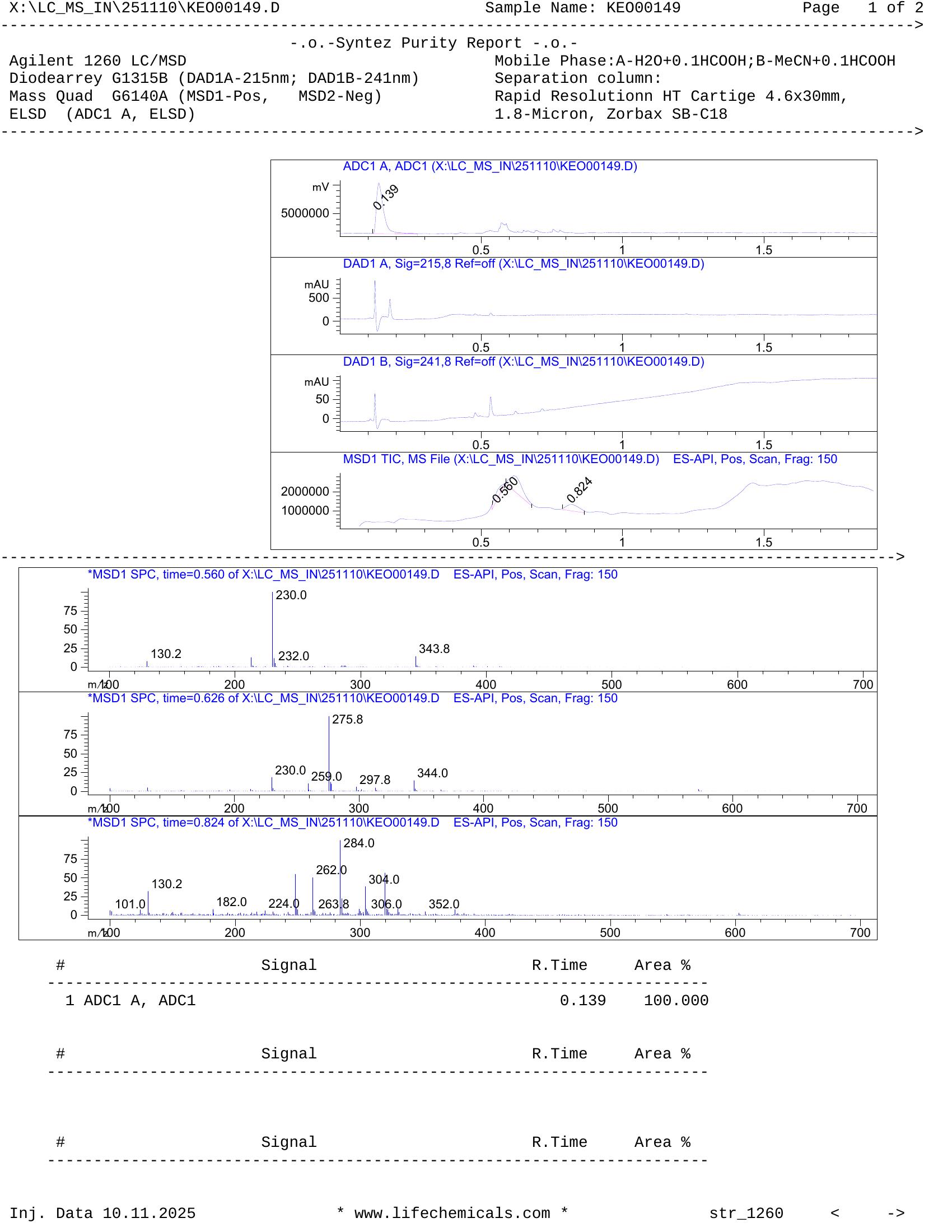


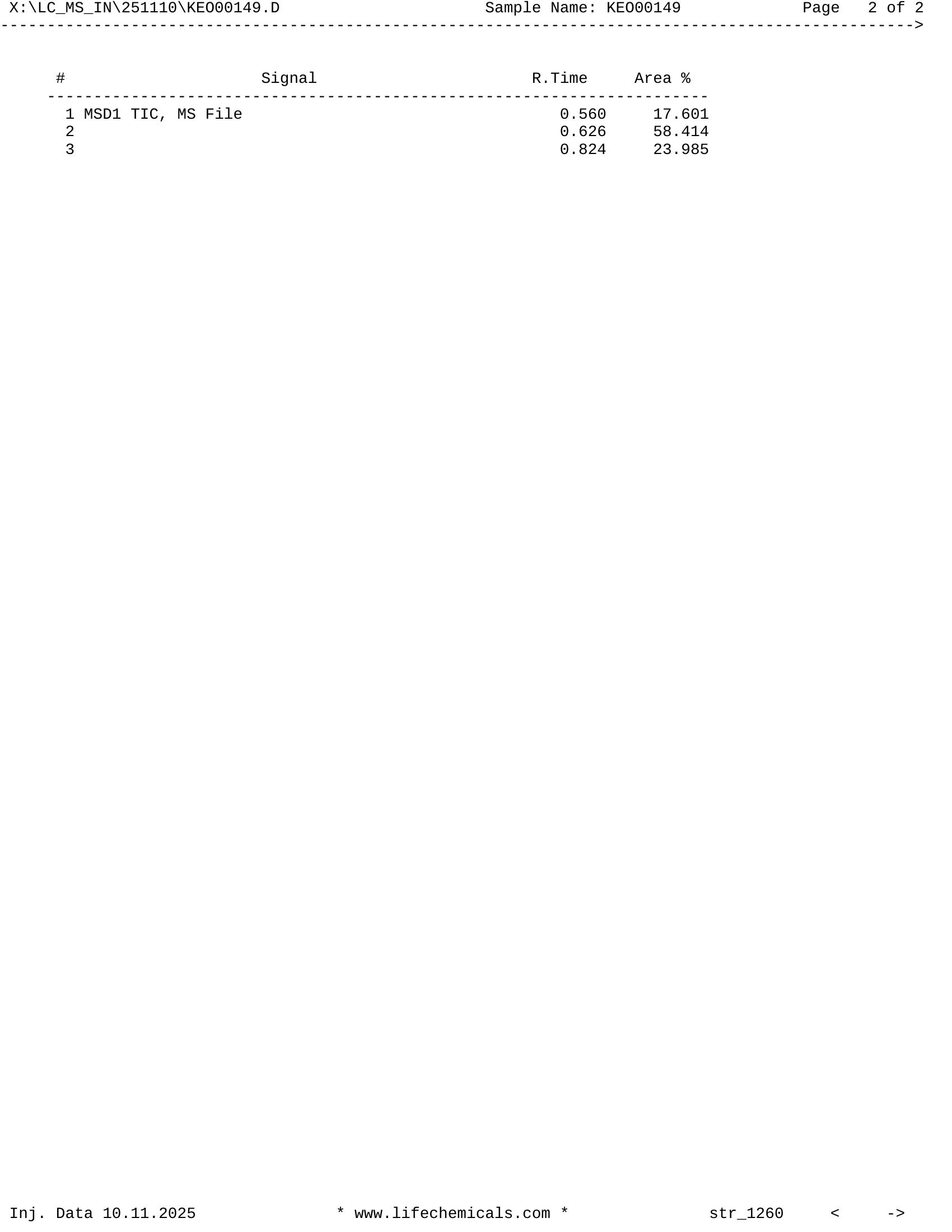


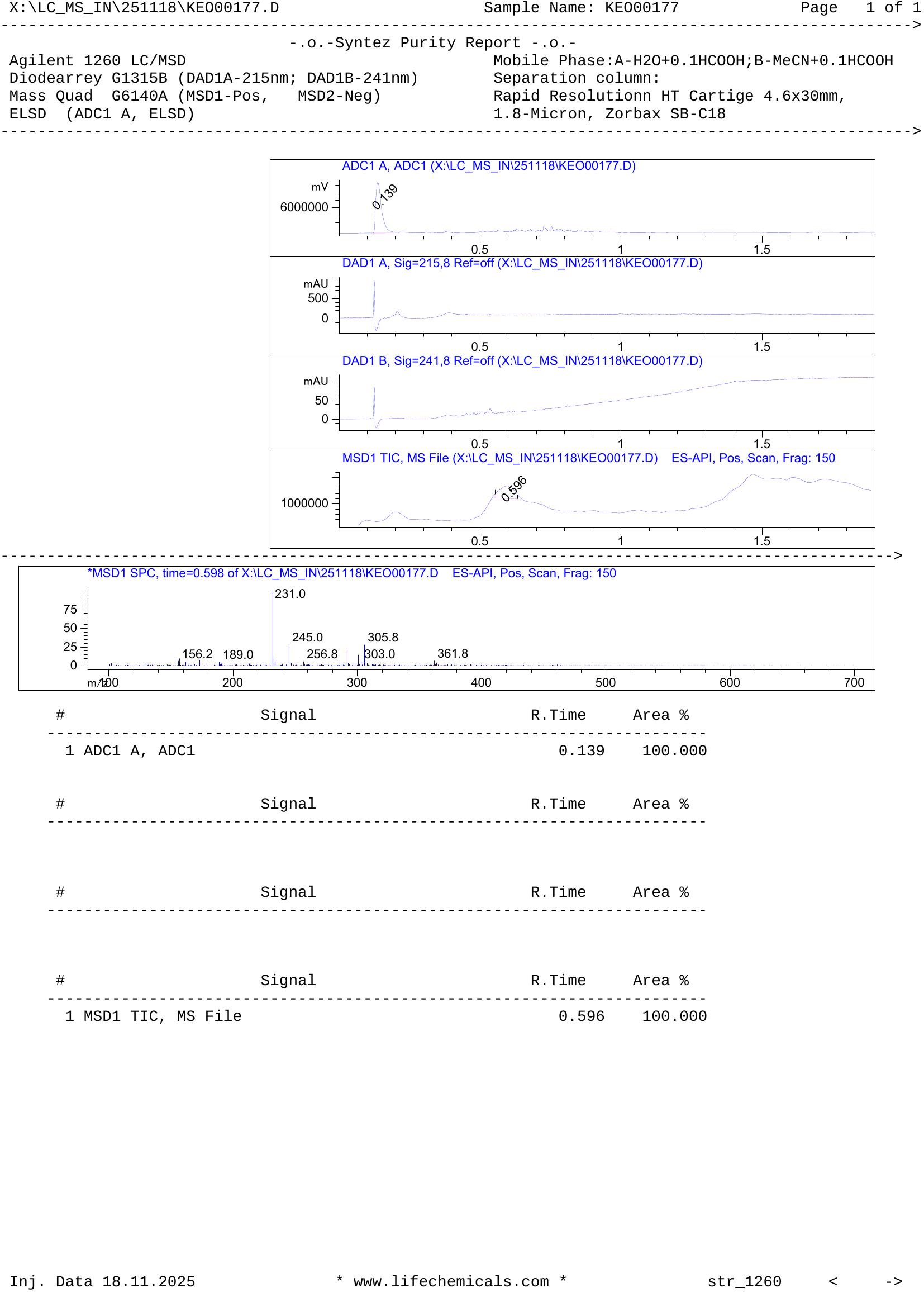

Supplement: Supplementary file 1 — Supporting Information Additional supporting information can be found online in the Supporting Information section. The complete 1H and 13C NMR spectra, LC‐MS data, and elemental analysis data for all synthesized compounds. [file BMRI-2026-4477854-s001.docx]
